# Methods Using Marine Aquatic Photoautotrophs along the Qatari Coastline to Remediate Oil and Gas Industrial Water

**DOI:** 10.3390/toxics12090625

**Published:** 2024-08-24

**Authors:** Roda F. Al-Thani, Bassam T. Yasseen

**Affiliations:** Department of Biological and Environmental Sciences, College of Arts and Sciences, Qatar University, Doha P.O. Box 2713, Qatar; ralthani@qu.edu.qa

**Keywords:** heavy metals, macroalgae, mangrove, microorganisms, phycoremediation, seagrasses, seaweeds

## Abstract

Qatar and other Gulf States have a diverse range of marine vegetation that is adapted to the stressful environmental conditions of seawater. The industrial wastewater produced by oil and gas activities adds further detrimental conditions for marine aquatic photosynthetic organisms on the Qatari coastlines. Thus, these organisms experience severe stress from both seawater and industrial wastewater. This review discusses the biodiversity in seawater around Qatar, as well as remediation methods and metabolic pathways to reduce the negative impacts of heavy metals and petroleum hydrocarbons produced during these activities. The role of microorganisms that are adjacent to or associated with these aquatic marine organisms is discussed. Exudates that are released by plant roots enhance the role of microorganisms to degrade organic pollutants and immobilize heavy metals. Seaweeds may have other roles such as biosorption and nutrient uptake of extra essential elements to avoid or reduce eutrophication in marine environments. Special attention is paid to mangrove forests and their roles in remediating shores polluted by industrial wastewater. Seagrasses (*Halodule uninervis*, *Halophila ovalis*, and *Thalassia hemprichii*) can be used as promising candidates for phytoremediation or bioindicators for pollution status. Some genera among seaweeds that have proven efficient in accumulating the most common heavy metals found in gas activities and biodegradation of petroleum hydrocarbons are discussed.

## 1. Introduction

The State of Qatar is on a peninsula located on the north-eastern coast of the Arabian Peninsula. It extends from the Arabian Desert as an outcrop in the Western Arabian Gulf in an area that is warm and humid, although the land area is arid or semi-arid and highly saline. Hence, this part of the Arabian Gulf and the marine vegetation are influenced by warm waters and the high salinity of the seawater of the Gulf. The common type of landscape comprises rocky desert, depressions, and salt marshes, and in general, it is flat to undulating. Seawater surrounds the Qatari peninsula except at the southern part, which borders the Kingdom of Saudi Arabia. 

The Qatari peninsula and other Gulf States have a diverse range of marine vegetation, and the seawater is rich in many species of marine organisms that are adapted to the unique environmental conditions of the region [1]. In general, the marine aquatic photoautotrophs include higher plants, algae (micro- and macro-algae), fungi, and bacteria. Notably, the prominent marine vegetation found at the coastline of the peninsula includes (1) mangrove plants and associated microorganisms, (2) seagrasses, (3) seaweeds, and (4) phytoplankton. During the last decades, many reports have discussed the mechanisms of bioremediation, phytoremediation, and phycoremediation using various types of bacteria, fungi, algae, and plants in Qatari ecosystems. This review discusses the biodiversity in seawater around Qatar, as well as remediation methods and metabolic pathways to reduce the negative impacts of heavy metals and petroleum hydrocarbons produced during these activities. 

## 2. Metabolic Reactions and Modern Biotechnology

Van Aken et al. [2] examined the uptake of organic compounds that result from anthropogenic and industrial oil and gas activities. These compounds are classified in different ways. For example, Kamath et al. [3] classified petroleum hydrocarbons using two main groups: (1) gasoline range organics (GROs) and (2) diesel range organics (DROs). GROs include mono-aromatic hydrocarbons such as benzene, toluene, ethylbenzene, and xylenes (BTEX), as well as short-chain alkanes (C6-C10), while DROs include longer-chain alkanes (C10-C40) and hydrophobic chemicals such as polycyclic aromatic hydrocarbons (PAHs). Wang et al. [4] classified petroleum hydrocarbons as follows: (1) n-alkanes, (2) iso-alkanes, (3) cycloalkanes, and (4) aromatics. The most biodegradable groups among petroleum hydrocarbons are n-alkanes (saturated hydrocarbons, C_n_H_2n+2_). Other chemical compounds have also been reported as organic pollutants in soils and waters, including 1,1,1-trichloro-2–2-bis-(4′-chlorophenyl) ethane (DDT), benzo[α] pyrene, pesticides, chlorinated solvents, explosives, PAHs, dioxins, and polychlorinated biphenyls (PCBs) [5,6,7,8,9].

Notably, not many plants are able to metabolize these compounds, as their actions on them are limited. Thus, large-scale degradation is not possible unless microorganisms actively contribute to these reactions. Catabolic enzymes that can achieve complete metabolism of these compounds in plants are either inefficient or unavailable. Partial degradation of these compounds could result in some organic components that can be stored in the vacuoles of these plants [4,10]. Reports have described some metabolic reactions of the degradation of petroleum hydrocarbons. The degradation of n-alkanes could lead to some useful metabolites such as acetyl-Co A and fumaric acid, which readily contribute to Krebs-cycle reactions, fatty-acid biosynthesis, and amino-acid interconversions. These reactions are considered as crucial events in plant metabolic pathways because they convert dangerous toxic compounds to useful metabolites. 

Complete degradation of petroleum hydrocarbons is affected by many factors, including environmental conditions, the cooperation between plants and their associated microorganisms, and the expression of genetic materials in the soil–microbe–plant system. During the last decade, numerous studies have discussed the impact of these factors on the degradation of pollutants produced by oil and gas activities. For example, Gkorezis et al. [11] examined plant-associated bacteria systems in detail to phytoremediate petroleum hydrocarbons and restore polluted sites. They attributed the ability of bacteria to degrade petroleum hydrocarbons to genes that control the productions of enzymes that catalyze and enhance the biodegradation processes. The cooperation between bacteria and plants is decisive in achieving complete metabolism of petroleum hydrocarbons as plants provide some exudates, while bacteria synthesize plant hormones while suppressing ethylene production and the mobilization of essential elements.

Many studies have discussed the impact of environmental factors on the degradation of these compounds. The optimum temperatures may vary according to the microbial species and the compound being biodegraded [12], while pH affects the microbial growth and enzyme activity that control the biodegradation of petroleum hydrocarbons. Furthermore, these reactions have different pH optima for growth and degradation [13], and biodegradation rates may decrease under alkaline conditions [14]. 

Nutrient availability is another factor that might affect the growth of bacteria. Essential elements such as nitrogen, phosphorus, and some trace elements are reported to have great impact on the degradation speed and can result in complete or partial degradation [15,16] Oxygen is also a limiting factor, especially in anaerobic environments. As most biodegradation processes are aerobic, oxygen is required for microbial metabolism [17]. Adequate moisture is also necessary for microbial growth and activity, and extreme moisture content can inhibit the process of biodegradation [18]. 

Microbial activity is negatively affected by salinity. Some halophilic bacteria that are recognized in Qatari sabkhas could be biologically active in such habitats. These bacteria perform various roles and activities in supporting native plants to resist salinity and have applications in biotechnology and the pharmaceutical industry, as well as the production of some useful compatible solutes [9,19,20,21,22,23]. Another factor is the composition of petroleum hydrocarbons. High concentrations can cause some toxicity to the microbial activity and could lead to a great reduction in the rate of biodegradation. Moreover, some other organic components are added during oil and gas activities, such as mono-, di-, and tri-ethylene glycol (MEG, DEG, and TEG, respectively) and kinetic hydrate inhibitors (KHIs). These compounds are present in the wastewater and might have negative impacts on the growth of plants when they are irrigated with such wastewater [10,21,24,25,26].

The internal factors in the soil biota might play important roles in the biodegradation of organic pollutants and stabilization of inorganic components such as heavy metals. These factors encompass the cooperation between plants and their associated microorganisms and the genetic features of these living organisms. In this respect, native plants or algae (as autotrophic living organisms) might play roles that are independent of the microorganisms’ actions, but in nature, it is difficult to separate the impact of plants from their associated microorganisms in degrading petroleum hydrocarbons. These microorganisms include rhizospheric, phyllospheric, and endophytic bacteria and fungi that interact with the secretions of plants. 

Many native plants secrete and release substances into the rhizosphere, and these exudates may help in encouraging microorganisms to degrade organic compounds of various types, as well as stabilizing heavy metals in the soil adjacent to the root systems of plants. Some interrelated activities and conditions take place at the rhizosphere. For example, plant roots exude various substances that can stimulate microbial growth and activity, and the microbes can degrade various toxic organic compounds completely or partially [21,27,28]. 

Furthermore, some plants absorb these organic compounds, and inside the plant tissues, these compounds might be further degraded into useful metabolites. Otherwise, toxic compounds are stored in the vacuoles to avoid their detrimental impacts on the plant metabolism [10,21,29]. Plants or microorganisms alone are also less efficient in metabolizing organic compounds than when they work together [30]. Thus, microbes play crucial roles in alleviating the impact of organic contaminants on the plant and human health by degrading toxic organic compounds into less toxic or into useful metabolites that can be absorbed by the plant and contribute to the metabolic pathways [21,31]. 

Plants and microorganisms cannot work alone in bio-transforming complex organic compounds of industrial wastewater into simpler units. Numerous studies have confirmed the active cooperation between plants and their associated and/or adjacent microorganisms, as such a combination could provide some resistance against various environmental conditions, including pollution. Notably, plant roots release various substances such as exudates, secretions, lysates, plant mucilage, and mucigel that might have significant roles in remediation of extreme environmental conditions such as pollutions [22]. Moreover, the work of Alves et al. [32] confirmed the role of the microorganisms associated with plants in immobilization of heavy metals. Some successful studies have elucidated the influence of separation of plants from associated microorganisms using hydroponic systems, sterile plant roots, and inoculating some bacteria to remove organic compounds such as phenol [33].

There are many possibilities of such relationships. Symbiotic relationships with microorganisms such as mycorrhizal fungi and nitrogen-fixing bacteria can support the plant’s ability to transform contaminants [34]. Relationships may also be synergistic. For example, certain microbes can break down complex organic contaminants into simpler compounds that can be absorbed readily and metabolized inside plant tissues [35,36]. Plants and microorganisms also produce enzymes that can degrade petroleum hydrocarbons, but these groups of enzymes are different, and distinguishing between plant-derived enzymes and microbial enzymes is very difficult unless advanced molecular techniques are used [4,37,38,39,40,41,42].

Plants can also enhance microbial activity by adding some nutrients that stimulate indigenous microbes or introduce specific microorganisms. This situation further complicates the attribution of each group of living organisms alone in the degradation of petroleum hydrocarbons. Sterile conditions may be exploited by designing experiments to distinguish the role of plants from microorganisms separately. Using sterile hydroponic systems for plant growth to avoid the interference of associated microorganisms can solve the issue of the difficulty of separating the effects of each group alone. Furthermore, the isolation of a pure culture of a microorganism can be used to test the ability of each microbial species to transform organic petroleum hydrocarbons into simpler compounds [33,43].

## 3. Genetic Factors and Modern Biotechnology

The genetic factors involved in the bioremediation of petroleum hydrocarbons cover a wide range of aspects involving different microbial communities, genetic adaptation, and the expression of genes involved [4,44]. Many microorganisms contain genes that control the degradation of petroleum hydrocarbons [45], and numerous microorganisms (bacteria and fungi) in Qatari soils are activated when irrigated with industrial wastewater and its various components. During the last decade, studies have identified many microorganisms in Qatari lands and seawater of the Arabian Gulf. For example, Al-Sulaiti et al. [25] identified many bacteria and fungi species using modern techniques, the VITEK system (bioMerieux-Vitek, Hazelwood, MO, USA) and the API 20 C AUX identification system (REF 20 210 of bioMerieux SA) for bacteria and fungi (yeast), respectively. Bacteria species included (1) *Staphylococcus* spp. such as *Staphylococcus sciuri* and *Staphylococcus lentus*; (2) *Lactococcus lactis;* (3) *Micrococcus luteus;* (4) *Kocuria kristinae;* (5) *Bacillus megaterium;* (6) *Pseudomonas* spp. such as *Pseudomonas aeruginosa*, *Pseudomonas stutzeri,* and *Pseudomonas putida*; (7) *Stenotrophomonas maltophilia;* (8) *Sphingomonas paucimobilis*; (9) *Burkholderia* spp. such as *Burkholderia cepacia* and *Burkholderia pseudomallei*; and (10) *Enterobacter cloacae*. Soils exposed to various changes in their compositions, abiotic factors, and biotic factors, including petroleum hydrocarbon pollution from oil and gas activities, might activate these species and others, such as *Bacillus* spp., *Pseudomonas geniculata,* and *Achromobacter xylosoxidans* [21,46,47].

Yeast species that were identified in such soils showed changes under the same conditions facing bacteria. The following yeast species were the most common in all soil and sand cultures: (1) *Candida* spp. such as *Candida tropicalis*, *Candida famata,* and *Candida guilliermondii*; (2) *Trichosporon mucoides*; and (3) *Cryptococcus* spp. such as *Cryptococcus humicola* and *Cryptococcus albidus*. These microorganisms could contribute to activities in the soil biota in Qatari lands, especially when polluted with industrial wastewater. Therefore, microorganisms might be promoted to play significant roles in the polluted soil, but the conditions of seawater from the Arabian Gulf are completely different from those of the lands. It is very difficult to distinguish the influence of microorganisms (bacteria and fungi) from that of seaweed, seagrass, and phytoplankton. 

Findings suggest that such activation of microorganisms to degrade spilled petroleum hydrocarbons might occur in seawater as well. Using modern biotechnology and phylogenetic analysis, numerous bacteria species have been identified, including Proteobacteria (*Cobetia marina*, *Halobacillus profundi*, *Pseudoalteromonas agarivorans*, *Pseudoalteromonas piscicida*, *Pseudoalteromonas rubra*, *Pseudoalteromonas prydzensis*, *Ruegeria mobilis*, *Shewanella loihica*, *Virgibacillus dokdonensis*, *Vibrio harveyi*, *Vibrio nereis*, *Vibrio nigripulchritudo*, *Vibrio parahaemolyticus*), Cytophaga–Flavobacterium–Bacteroides (CFB) bacteria (*Tenacibaculum mesophilum*), and Firmicutes (GC) (*Bacillus boroniphilus*) [48]. Infectious *Vibrio* bacteria species have also been recognized and investigated in terms of their roles [22,49,50].

Microorganisms of marine sediments contribute significantly to the functions related to the degradation of organic compounds of anthropogenic and industrial origin [51,52]. Notably, few works and baseline investigations have examined the potential of microorganisms to degrade industrial wastewater pollution in the seawater of the Arabian Gulf since few comparisons have been made between ecosystems, especially regarding the microbial ecology and functions in the marine sediments surrounding the Qatari peninsula. However, microbial communities either decrease or increase in response to pollution with petroleum hydrocarbons from industrial wastewater [53].

Regarding solutions to the environmental issues, three strategies have been suggested and applied during the last four decades to improve the efficiency of living organisms to restore polluted soil and water, including plants, algae, and microorganisms. These strategies are environmental, genetic, and biological approaches. Environmental manipulation has been adopted earlier for the reclamation of saline lands and to remove toxic ions from agricultural lands. However, the environmental approaches have many drawbacks and are not always an applicable option, especially for seawater [54].

Genetic approaches have been suggested as promising options to solve many biotic and abiotic problems [21,55]. Modern biotechnology and genetic engineering programs have been considered as future solutions for many problems related to agriculture, health, economy, and pollution. Modern biotechnological and biological options could be applied together to solve many pollution issues. For example, transgenic plants and microorganisms might be more efficient in the de-toxification of pollutants, remediation of heavy metals, and metabolism of petroleum hydrocarbons [21,54,56,57,58,59,60].

## 4. Mangrove Plants and Associated Microorganisms

**Figure 1:**
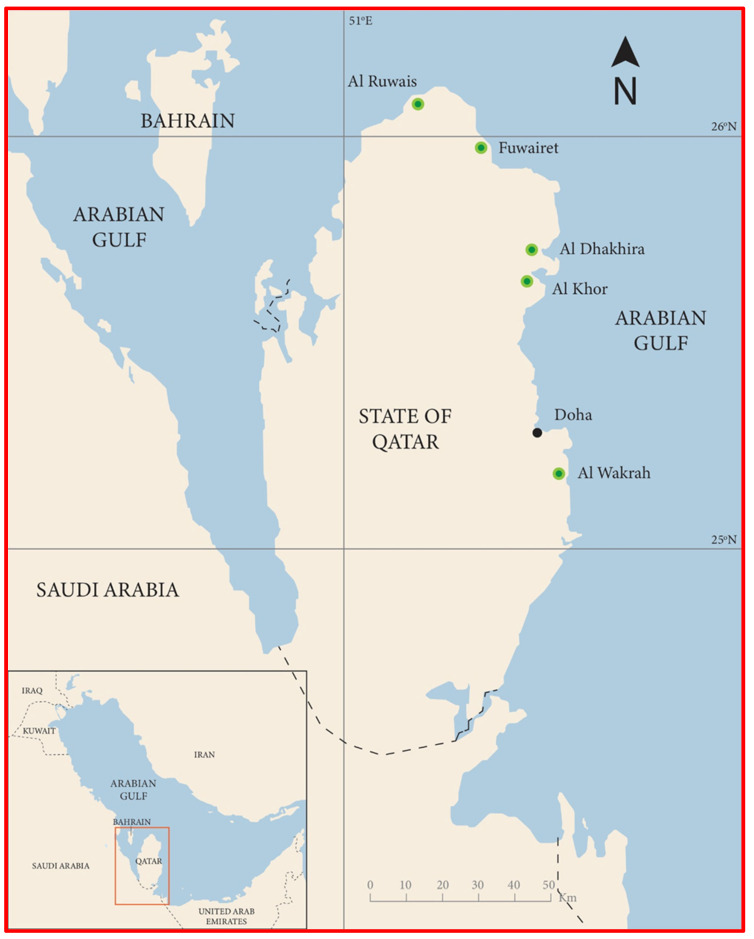
Map of Qatar showing the locations of mangrove forests on the eastern coastline.

**Figure 2:**
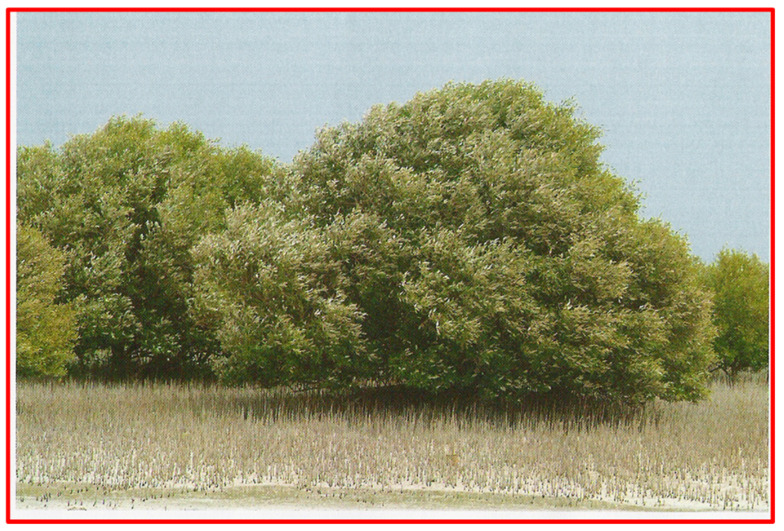
*Avicennia marina* is the only species represented in mangroves in Qatar. A part of a mangrove forest showing trees and propagules.

Mangrove forests are found on the eastern and north-eastern coastlines of Qatar, with large stands in Al-Dhakhira, Fewairet, Al-Reweis, Al-Khor, and Al-Wakra (Figure 1). These forests cover an area of about 981 ha and represent most of the existing stands in Qatar [61]. Only one species, *Avicennia marina,* is represented in the mangrove forests in Qatar. This species is internationally known as grey mangrove and is an evergreen dark green tree or shrub that does not exceed 4 m in height. It also produces propagules (Figure 2) and a cable network of breathing roots called pneumatophores (Figure S1).

Notably, mangrove forests are common at muddy shorelines, especially along the north-central parts of the eastern coastline in the tidal zones. These forests were the main source of wood and camel fodder in the past, but currently, they are semi-protected as a result of extensive camel grazing. Many studies have examined the ecophysiology of the plants in this area and the structural features that enable them to resist the high salinity levels of the seawater.

The mangrove forests have several important roles in the ecosystem, such as reducing erosion on the coastlines. Karimi et al. [62] have discussed the role of *A. marina* in stabilizing the coastline by trapping sediments with its extensive root system, which reduces erosion caused by waves and tidal action. Another role is the conservation of marine and terrestrial species, which include fish and crustaceans, as well as providing nesting sites for birds and feeding numerous organisms [63].

The mangrove forests also support the biodiversity of various plant and animal species by providing shelter, food, and mini-habitats for breeding for various organisms such as fish species [64]. They also improve water quality by acting as natural filters. This is accomplished by many methods, such as trapping of organic and inorganic pollutants and sediment from runoff and allowing them to settle to the bottom of the sea, as well as absorbing nutrients and removing contaminants from the seawater. These two methods help to make the marine environment safer and healthier [65,66].

The mangrove forests also function as carbon sinks, and many studies have shown that they are efficient in sequestering CO_2_ from the atmosphere. This occurs through the storage of large amounts of carbon in biomass and the soil beneath the mangrove trees. Thus, mangroves could help to mitigate climate change [67]. Furthermore, they provide protection against storms, which helps coastal communities. The forests act as a natural barrier that protects inland areas from flooding and property damage during waves of storms, hurricanes, and other extreme weather events [68].

Lastly, *A*. *marina* has economic value and can be used as a medicinal plant. It provides many advantages to the local communities, including fishing, tourism, and protection of shorelines, as discussed above. It has edible seeds for humans with high amounts of vitamins and carbohydrates, and its leaves are edible and preferred for cattle and camels. Some of its organic components, such as betulic acid, taraxerol, and taraxerone [69], as well as other sterol components, could make it beneficial as a medicinal plant for numerous health applications, including smallpox, aphrodisiacs, poultices, and accelerated suppuration of boils and abscesses [70,71].

### 4.1. Phytoremediation

In general, the ability of native plants to remediate polluted soils and waters is limited by different factors, including environmental and genetic factors. However, many reports have shown some success in absorption, translocation, sequestration, and degradation of petroleum hydrocarbons [72]. Numerous elements (including heavy metals such as As, Cd, Co, Cr, Cs, Cu, Fe, Hg, Mn, Ni, Pb, and Zn) have been reported to be remediated by Qatari native plants by different methods [10,21,73,74] (Table S1). However, the remediation of organic components has been a main concern of scientists and research centers, as very little has been accomplished to show the degradation of organic components from oil and gas industrial activities by Qatari native plants (Table S2). Some native flora of Qatar have proven efficient in remediating industrial wastewater produced during anthropogenic and industrial activities, such as *Phragmites australis*, *Typha domingensis*, *Sporobolus* spp., *Medicago* spp., and possibly others [10,25]. More data can be found in more supplementary articles which include tables (Tables S3 and S4). 

Wild mangrove plants could be promising candidates for phytoremediation and phyto-management due to high biomass production and their adaptation to seawater. Particularly, *A. marina* has proven efficient in phytoremediation. Many studies from around the world in the last 20 years has confirmed this, and various related plant species can remove and remediate heavy metals and organic petroleum hydrocarbons. These plants and their associated microorganisms can efficiently remove heavy metals and metabolize organic petroleum hydrocarbons [75,76,77]. Early reports by Abdel-Bari et al. [78] found that *A. marina* from the eastern coast of Qatar (Ras Al-Matbakh) accumulated many heavy metals, such as Co, Cr, Cu, Fe, Ni, and Zn. Moreira et al. [75] found that *Avicennia schaueriana* is more efficient in the phytoremediation of petroleum hydrocarbons than microorganisms in benthal sediments. Such findings show that phytoremediation using mangrove plants is a promising strategy for coastlines impacted by wastewaters from the oil and gas industry.

In addition, the growth of microorganisms in the sediments might be stimulated by secretions produced by these plants, which facilitates degradation of organic components of petroleum hydrocarbons and leads to useful metabolic compounds [10,21,26]. Furthermore, interestingly, Moradi et al. [79] found that the activity of peroxidases such as ascorbate peroxidase and polyphenol oxidase increased in soil contaminated with organic components of oil, while superoxide dismutase decreased. These findings suggest that mangrove plants such as *A*. *marina* are promising candidates for phytoremediation of oil spills and residual oil pollution in coastal marine environments.

Modern technologies can be adopted and applied to improve the ability of these plants to degrade, metabolize, and accumulate petroleum hydrocarbons and heavy metals in plant tissues [80]. Moreover, modern biotechnology and genetic engineering could be used to develop active remediators to deal with various types of pollutants [21,26,60,81]. Worldwide records show that mangrove forests provide numerous supports to the ecosystem, and these reports cover many methods of phytoremediation where microorganisms work together with these plants [59,76,82]. These methods include (1) phytoextraction, (2) phytostabilization, (3) phytodegradation, (4) phytovolatilization, and (5) rhizofiltration [59,74,83,84].

To use such plants in phytoremediation processes, monitoring would be necessary to keep the ecosystem safe. The plant biomass can be recycled or used in industry to extract heavy metals [10,21]. The research performed thus far could be a source of information and experimental material for further research to increase the efficiency of these plants in remediating polluted soils and waters in the Arabian Gulf region.

Recent reports have discussed the related metabolic pathways [4,10,21] of native plants and crops that can absorb harmful petroleum hydrocarbons from contaminated soils and aquatic habitats [26]. Notably, numerous plants and their associated bacteria (including endophytic, phyllospheric, and rhizospheric bacteria) can metabolize petroleum hydrocarbons [11] and produce useful metabolites that might contribute to the metabolic pathways in plants [10]. Varjani [85] reviewed bioremediation for petroleum hydrocarbon pollutants and explanations of their metabolism in microorganisms. Some plants can absorb and transport petroleum hydrocarbons, including heavy metals, which can be sequestered in the root tissues or transported into the shoots and leaves, where they can be stored in vacuoles or volatilized into the atmosphere [11,59,86]. At least three heavy metals that are normally found in the industrial wastewater from gas activities (As, Hg, and Se) can be volatilized using native plants. These plants include swamp lily (*Crinum americanum*), water hyacinth (*Eichhornia crassipes*), hydrilla (*Hydrilla verticillata*, Royle), duckweed (*Lemna minor*, *Lemna obscura*), water lettuce (*Pistia stratiotes*), water moss (*Salvinia natans*), cattail (*Typha domingensis*), and possibly others [87,88,89].

Recent evidence has shown that aquatic plants in Qatar, such as *Phragmites australis*, *Typha domingensis*, and perhaps others, are efficient in remediating industrial wastewater from oil and gas activities [10]. Moreover, the growth of *P*. *australis* was improved by industrial wastewater, which can be explained by the activities of rhizospheric bacteria in providing some minerals and metabolites to the plant during the phytoremediation processes of petroleum hydrocarbons [21,26]. Autotrophs can utilize mineral nutrients and some metabolites, while some toxic compounds that result from partial degradation of organic compounds are sequestered in the vacuoles of living aquatic organisms such as algae, mangroves, and seagrasses [10,74].

### 4.2. Perspectives of Cultivation of Mangroves in Qatar

The peninsula of Qatar has a coastal length of about 563 km, and its coastline could be suitable for the cultivation of mangrove plants to achieve the benefits discussed thus far. This would involve a major governmental project that should consider all obstacles that might be faced and would require comprehensive scientific investigations to address all the challenges. Almahasheer [90] discussed the spatial coverage and distribution of mangroves, including extreme geographic and ecological factors in the Arabian Gulf region. They confirmed that mangroves in Qatar and other countries in the Arabian Gulf region have remained stable with a slight increase. Five steps that should be considered before conducting any real practical project have been suggested: (1) obtaining information, (2) recognizing problems, (3) setting plans, (4) finding solutions, and (5) maintaining sustainable monitoring [91].

Some studies from the last five years have indicated that compensatory planting of mangroves in new areas may be a successful long-term strategy for supporting benthic biodiversity. Furthermore, it could contribute to the mitigation of climate change by increasing long-term carbon storage [92]. Other recent studies on the benthic biodiversity in mangrove forests in Qatar showed that this plant could affect the biodiversity of some marine fauna and, possibly, all communities driven by environmental factors such as salinity and temperature [93]. Nevertheless, more studies are needed to determine the complete impact of mangrove forests on the biodiversity in these areas.

Abdel-Bari et al. [78] studied the ecophysiology of *A. marina* among other halophytes in Qatar. This plant accumulates the main ions found normally in the seawater, such as Na, Cl, Ca, and Mg. Using an exclusion mechanism, these ions are filtered by the root system to prevent the build-up of salts in the conducting system that leads to the green parts at the top of the plant. Notably, many ions are still transported to the leaves, where some of them accumulate, while many of them are excreted by salt glands [94] (Figure S2). This might attract camels and cattle to feed on the green leaves of the plant, which could endanger mangrove forests and possibly result in their disappearance. Therefore, this plant has developed major mechanisms to avoid high levels of salinity from seawater [74] (Figure S3).

Some chemical methods of soil and plants and modern techniques have been adopted for protection, management, and conservation of such the mangrove ecosystem in Qatar [95]. Mangroves have good nitrogen availability and high amounts of photosynthetic pigments. Remote sensing techniques have proven efficient in chlorophyll prediction and estimation. One major method of salt tolerance is osmoregulation, and some compatible osmolytes such as proline and glycinebetaine are accumulated in the cytoplasm of plant cells to balance the absorbed toxic ions that accumulate in the vacuoles [94,96].

## 5. Seagrasses

Seagrasses are flowering plants found in shallow coastal waters, where they are anchored to the seabed by roots that can tolerate saline seawater. Seagrasses are the only true marine plants that can live completely submerged under water, and the depth at which they are found is limited by water clarity, which determines the amount of light reaching the plant. Notably, seagrasses are a critical component of coastal components and provide various ecological and environmental benefits. One benefit is that they provide an essential habitat for various types of marine organisms, such as fish, invertebrates, and various species of algae [97].

Furthermore, seagrass improves water quality by trapping sediments and filtering out pollutants. Lee et al. [98] found that the seagrass *Zostera marina* is efficient in phyto-remediating heavy-metal-contaminated coastal sediments. They found that this plant accumulated substantial amounts of heavy metals, such as As, Cd, Co, Cu, Fe, Hg, Pb, and Zn. Many of these heavy metals are the main components of industrial waste water from gas activities.

Seagrass also protects the coastline and reduces coastal erosion by stabilizing sediments and attenuating wave energy [99], and it helps combat climate change by storing significant amounts of carbon in its biomass and sediments. Removing carbon dioxide from the atmosphere by photosynthesis helps to mitigate the negative impact of continuous increases in the carbon around the globe [100]. Seagrasses also support marine ecosystems by producing oxygen via photosynthesis [101]. Three main species of seagrass are found in the Arabian Gulf, which are monocots: *Halodule uninervis*, *Halophila ovalis*, and *Thalassia hemprichii*.

### 5.1. Halodule uninervis (Forssk.), Syn. Zostera uninervis Forssk

*H. uninervis* is an indigenous perennial seagrass with long rhizomes and linear leaves at the nodes. This widespread seagrass lives at shallow depths and forms dense meadows. This plant provides an important habitat and feed for marine organisms, and as such, its roles are being monitored in some Arab countries in the Arabian Gulf, such as the United Arab Emirates, Oman, and Qatar. Yasseen and Al-Thani reviewed the research on its role in the desalination of seawater [74].

Remediation of seawater includes the removal of Na^+^, Cl^−^, K^+^, and some trace elements such as Cu, Fe, Ni, and Pb [102]. When such trace metals accumulate in the plants, they end up in the food chain and cause contamination of the ecosystem. Some organic compounds might be remediated by this plant as well [103,104,105]. Recent studies have shown that this plant might have an active role in removing carcinogenic polycyclic aromatic hydrocarbons, and dead leaves from this plant were used to adsorb petroleum hydrocarbons such as acenaphthylene, phenanthrene, and fluoranthene from seawater [106].

### 5.2. Halophila ovalis (R.Br.) Hook.f., Syn. Caulinia ovalis R. Br. (1810)

*H. ovalis* is a dioecious perennial seagrass that occurs at shallow depths with opposite ovate leaves. It has been extracted from waters in the areas of Ras Al Noof near Alkhore, Eastern Qatar. It is also commonly known as paddle weed, spoon grass, or dugong grass and belongs to the family Hydrocharitaceae. It is a small, herbaceous plant that occurs in sea beds and other saltwater environments. It is often found in meadows that dominate sand banks or other patches of the sea floor.

Early reports showed that this seagrass can be used as a bioindicator of pollution by various petrochemical compounds and heavy metals, such as Cu, Cd, Pb, and Zn, which is achieved by testing the chlorophyll fluorescence [107,108,109]. Ralph [110] used this seagrass to examine herbicide toxicity by adopting the same techniques. Runcie et al. [111] reviewed the toxic effects of petrochemicals on this seagrass, as well as morphological, structural, and physiological aspects. They discussed the resulting reduction in their resistance to other stress factors, reduction in growth rate, and toxic appearance on leaves and flowers that might lead to the death of the plant. Petrochemicals might break down the waxy cuticle, which leads to more penetration of toxic compounds and phytotoxicity. Furthermore, disturbances in the ultrastructure of cell organelles such as thylakoids of the chloroplasts can occur.

Thus, *H. ovalis* is not typically considered a viable candidate for the remediation of organic compounds and heavy metals from industrial wastewater from oil and gas activities, but is considered as a bioindicator. However, this plant might play limited biological and phytoremediation roles in seawater and sediments. This plant is sensitive to contamination with petroleum hydrocarbons, as high levels of pollutants could have a negative impact on it. The pollutants can damage the tissues and reduce the photosynthetic growth apparatus.

Furthermore, the plant thrives in clear, shallow marine water where light easily penetrates to boost autotroph growth. This plant lacks the physiological and structural capabilities to remediate pollutants and their degraded components by metabolizing or sequestering them in the vacuoles. Lastly, petroleum hydrocarbons could mainly be degraded by microorganisms, as the role of this seagrass in secreting substances to encourage and boost microorganism activities is limited [109].

### 5.3. Thalassia Hemprichii

*T. hemprichii* is also called Pacific turtlegrass. This widespread species is native to the shores of the Indian Ocean, Red Sea, Western Pacific Ocean, and the Arabian Gulf region. This plant has strap-like or curved sickle-shaped leaves that are 0.5–1 cm wide and 7–40 cm long (usually less than 25 cm). The tips are usually rounded and smooth. The leaves may appear speckled due to tannin cells that appear red, purple, or dark brown. The growth rate of this plant increases with the enrichment of CO_2_, and it can tolerate lower light conditions caused by algal blooms.

Some studies have shown that this seagrass can accumulate high amounts of heavy metals such as Cd and Pb in the root system [112], while its ability to transport them to the shoot system is limited. This species adopts mechanisms similar to those described for *A. marina* [74]. While it is not a food source for humans or livestock, it has ecological roles as a source for marine herbivores such as crustaceans, fish, dugongs (marine mammals), and turtles [113].

The salts and other elements that are transported to the leaves and other parts of the plant are sequestered in the vacuoles, which is balanced by the accumulation of some compatible organic and inorganic solutes in the cytoplasm. Various research and review articles provide more details [9,114,115]. Phytoremediation of petroleum hydrocarbons using *T. hemprichii* has not been studied, but there are some indications that it might enhance microbial activities that stabilize and degrade these compounds. The degraded organic compounds might then be absorbed and metabolized inside the plant tissues [116].

## 6. Seaweeds 

Seaweeds are multicellular and macroscopic algae autotrophs that grow in coastal areas in many water bodies such as oceans, seas, rivers, and lakes. Taxonomically, they are categorized into three groups (Divisions) based on their photosynthetic pigments: Chlorophyta (green algae), Rhodophyta (red algae), and Phaeophyta (brown algae). These algae are non-vascular photosynthetic organisms that lack true roots, stems, and leaves and absorb nutrients and water directly from the surrounding water. They have different shapes and sizes and play crucial ecological roles in marine ecosystems. These roles cover various functioning aspects in these environments, including climate change, health, and economy.

One of their roles is the production of significant amounts of organic components, such as sugars and oxygen. Thus, these organisms provide a significant source of food and energy. They also create habitats and shelter for a wide range of marine life, such as fish, invertebrates, and microorganisms. This role includes protection from predators and providing places to attach and grow for sessile organisms.

Many fish species also use seaweed beds as a safe environment for feeding and hiding, which increases their survival rate. Furthermore, seaweeds are good source of food and dietary components for various marine herbivores, such as urchins, sea slugs, and fish, and nutrient cycling is another role these seaweeds can play. They absorb and store nutrients from around seawater, but after their decay, the nutrients are released and support the ecosystem.

Seaweeds also absorb CO_2_ and produce O_2_ during photosynthesis, which helps to stabilize the ecosystem and mitigate the great negative effect of climate change around the globe. Moreover, they support marine life through the respiration process. Seaweeds help stabilize the shoreline by reducing the impact of waves and currents. Their holdfasts (root-like structures) anchor them to the substrate and act as a natural barrier against coastal erosion [117,118]. Seaweeds can also be used for various medical and industrial uses, including pharmaceutical, cosmetic, and food applications, as they contain bioactive compounds that are used for their medicinal and nutritional properties.

Biodiversity support is another role that seaweeds play by providing diverse microhabitats for a wide range of organisms. This role might increase species richness and ecological interactions within the marine ecosystem. Phytoremediation (or phycoremediation) plays a crucial role in the filtration of polluted water, which can help improve water quality in marine environments. Many macroalgae have proven efficient in removing organic and inorganic pollutants [59,119,120]. Furthermore, these macroalgae play an ecological role as primary producers in seawater and sustain several benthic animal communities that contribute to the food chain. They can also act as bioindicators of water quality for bioremediation [21,22,26,121,122].

### 6.1. Chemical Constituents and Uses

Chemical components of seaweeds have various uses and advantages. These constituents include proteins; amino acids; minerals including heavy metals; vitamins (water and lipid-soluble vitamins); lipids; dietary fibers; antioxidant compounds; and antibacterial, antifungal, and antiviral compounds. Notably, some components could be useful as food for animals and humans and as medicines or pharmaceuticals to treat many diseases. 

All these components and their secondary metabolites, such as terpenoids, phenolic compounds, and compatible solutes, might be affected by environmental conditions and pollution levels in the Arabian Gulf. This is due to the continuous spills during transport, military exercises, and wars. It is not the objective of this article to discuss the roles of these components in seawater, which need further detailed follow-up to look into the impacts of these constituents on marine and human life in the Arabian Gulf. The potential for remediation of industrial wastewater by many seaweeds is discussed.

Rizk et al. prepared a list of seaweeds in the Arabian Gulf around the Qatari peninsula [123]. Table 1, Table 2 and Table 3 contain the species of seaweeds, their families, the main chemical constituents, and possible roles that they may play in the marine ecosystem. Their potential for remediation has been addressed in many studies. Further studies should examine their phycoremediation processes to avoid and reduce pollution in the Arabian Gulf region.

### 6.2. Phycoremediation

Phycoremediation is a promising method to utilize seaweeds such as green, brown, and red algae (macroalgae) to degrade or remove various pollutants from seawater. Macroalgae are efficient in these methods because their growth rate is significantly high, which leads to the production of substantial biomass, and they can absorb and accumulate many heavy metals, including those involved in oil and gas activities. They can also metabolize organic compounds produced during oil and gas extraction, processing, and transportation [124,125]. Seaweeds use at least four methods to remediate these pollutants: (a) biosorption, (b) bioaccumulation, (c) biodegradation, and (d) nutrient uptake [126,127].

**Table 1:** List of Chlorophyta (green algae) species and the possible constituents and roles.

Species	Family	Main Constituents	Possible Roles	References
*Acetabularia caliculus*	Polyphysaceae	Proteins (4.5%), lipids (4.2%), carbohydrates (33.4%,), and ash including minerals (57.3%), others (0.6%) as secondary metabolites such as phenolic compounds and terpenoids	Phycoremediation of: Cd, Cr, Cu, Hg, Ni, Pb, and Zn, organic components, and nutrients such as nitrogen and phosphorus, antioxidants	[123,128,129]
*Avrainvillea amadelpha*	Dichotomosiphonaceae	Rawsonol ^a^, isorawsonol-steroids, bromophenols, Sulfono-glycolipid	Antioxidants, anticancer, H_2_O_2_ scavenging activity, hemagglutination, antibacterial, heavy metal phycoremediation: (Cd, Cu, Pb) *	[123,130,131,132]
*Boodlea composita*	Boodleaceae	β-sitosterol, loliolide ^b^ and 13 ^2^ -hydroxy-(13 ^2^ -S)-phaeophytin-a, fatty acids, sterols, sulphated polysaccharide, agglutinins, glycinebetaine, prolinebetaine	Possible remediation role in polluted saline waters	[123,124,133,134,135,136]
*Bryopsis implexa*	Bryopsidaceae	Xylan, carotenoids, free amino and fatty acids, sterols, bryopsin, kahalalide F	Possible role in remediating polluted and saline waters, anticancer action	[119,123,130,137]
*Caulerpa mexicana*	Caulerpaceae	Siphonaxanthin ^c^, siphonein ^d^, various polysaccharides, fatty acids, amino acids	Degradation of petroleum hydrocarbons, possible removal of heavy metals, nutritional uses, medical uses: antiviral, antibacterial, etc.	[123,138,139,140]
*Chaetomorpha* spp., (5 species): *C. aerea*, *C*. *indica*, *C*. *linum*, *C*. *koeiei*, *C*. *patentarama*	Cladophoraceae	Sulphated polysaccharides; containing arabinose, and galactose, and other sugars such as glucose, xylose, and fucose, hemolytic saponin	Anticoagulant activities (antithrombin type), possibly toxic, remediation of IWW	[123,141,142,143,144,145]
*Cladophora* spp., (3 species): *C*. *koeie*, *C*. *patentirama*, *C*. *sericoides*	Cladophoraceae	Pigments such as β-carotene, xanthophyll, xanthophyll-epoxide, violaxanthin, and other related pigments, water-soluble sulphated polysaccharides, other related compounds, various types of amino acids	Phytoremediation of petroleum hydrocarbons; antibacterial and antiviral activities; antimitotic and cytotoxic activities; monitoring heavy metals such as Cd, Co, Cr, Cu, Fe, Hg, Mn, Ni, Pb, and Zn	[123,146,147,148,149,150]
*Cladophoropsis sundanensis*	Boodleaceae	Xanthophyll, loroxanthin siphonaxanthin	Little is known about role in phytoremediation; needs more investigation	[123,151,152]
*Dictyosphaeria cavernosa*	Siphonocladaceae	Alkylxanthate, bicyclic lipid, dictyosphaerin, some heavy metals	Possible phycoremediation of heavy metals, anti-mosquito larvae	[123,128,153,154,155]
*Enteromorpha* spp., (2 species): *E*. *kylinii*, *E*. *ramulosa*	Ulvaceae	Water-soluble polysaccharides, fatty acids and sterol, essential amino acids	Bioactivity such as hypocholesterolemic effect, antibacterial and diuretic activities, mutagenic activity, indicator of pollution	[123,156,157,158,159]
*Rhizoclonium kochianum*	Cladophoraceae	Scanty information, crystalline cellulose	Antibacterial, beta-blocker, 5-hydroxytryptamine blocker, folk medicine for burns, vermifuge, possible phycoremediation of heavy metals and organic compounds, nutritional value	[123,160]
*Ulva purtusa*	Ulvaceae	Polysaccharides, fatty acids, non-acidic glycolipid fractions, monogalactosyl, diglyceride, isofucosterol, amino acids, ascorbic acid (vitamin C), heavy metals such as Fe, Mn, Ti, Ni, Cu, Pb, and others	Bioindicator of seawater pollution, remediation of petroleum hydrocarbons and heavy metals	[123,158,161,162,163,164]

^a^ Rawsonol and Isorawsonol: polybromophenols (a type of terpenoid, an inhibitor), ^b^ β-sitosterol, loliolide is one of several phytosterols of plant sterols, ^c^ carotenoid, ^d^ C19 acylated siphonaxanthin. * Definition of heavy metal: the definition of heavy metals can come from three criteria. These are the density, atomic weight, and the behavior of a metal beyond a certain limit [165,166].

**Table 2:** List of Phaeophyta (brown algae) species and the possible constituents and roles.

Species	Family	Main Constituents	Possible Roles	References
*Colpomenia sinuosa*	Scytosiphonaceae	Cytotoxic fractions with complex mixture of saturated and unsaturated fatty acids, carotenoid fucoxanthin, some amino acids	Possible role of heavy metal remediation, its presence is a sign of pollution	[123,167,168,169]
*Cystophyllum muricatum*	Ceratophyllaceae	Little information available, presence of some fatty acids	Possible remediation of heavy metals and organic components	[123,170]
*Cystoseira* spp., (2 species): *C*. *myrica*, *C*. *trinodis*	Sargassaceae	A sulphated polysaccharide containing some soluble sugars, fucoidan, glycinebetaine and related compounds, alginic acid, uronic acid, laminaran, mannitol, amino acids, palmitic acid, lipid components, diterpenoids, etc.	Remediation of heavy metals in seawater	[119,123,163,171,172,173]
*Dictyota cervicornis*	Dictyotaceae	Fucoidan, diterpenes, diterpenoids, sterols such as fucosterol, phloroglucinol as toxic compound	Cytotoxic effects, many deterred feedings by some sea animals such as fish and sea urchins, etc., possible phytoremediation of heavy metals and petroleum hydrocarbons	[123,174]
*Ectocarpus mitchellae*	Ectocarpaceae	Mannitol, ectocarpene, fucoidan, alginin, ectocarpene	Sexual pheromone, hemagglutinin activity, possible remediation of heavy metals and petroleum hydrocarbons	[123,158,175]
*Giffordia mitchellae*	Acinetosporaceae	Giffordene, stereoisomers	Hemagglutinin activity, no reports about remediation, needs to be tested for phycoremediation	[123]
*Hormophysa cuneiformis*	Sargassaceae	Carbohydrates (59%), proteins (9%), lipids (7%), and ash (25%); sterols; fatty acids; amino acids; some heavy metals are found such as Fe, Zn, Co, Pb, Cu, Mn, and Al	Bioindicators for heavy metal pollution, anticancer and possible antimicrobial potential	[119,123,176,177]
*Padina australis*	Dictyotaceae	Sulphated heteropolysaccharides; fucan contained monosaccharides; neutral sugars such as arabinose, fucose, galactose, glucose, mannose, rhamnose, and xylose; other sugar component complexes are found such as uronic acid and fucosterol; fatty and amino acids such as glutamic acid, arginine, and proline	Anticoagulation activity, human HL-60 leukemia cell-line, bioactive primary and secondary metabolites with antibacterial activity against Bacillus spp and Staphylococcus spp., monitoring heavy metals, high capacity of the polyphenols for the chelating of heavy metals, possible heavy metal phycoremediation	[123,178,179,180,181]
*Sargassum* spp., (2 species): *S*. *aquifolium*, *S*. *boveanum*	Sargassaceae	Polysaccharides, sargassan: many monosaccharides in this compound are found; amino acids are found in the peptide portion; high fucoidan content containing some complex polysaccharides; high percentage of alginate and mannitol; fatty acids of various types are found; glycerides and many other complex compounds, etc.	High nutritional values, trace elements are found such as Ag, Al, As, Au, Ba, Ce, Co, Cr, Sr, Cs, Fe, Mn, Sb, Sc, Te, V, U, and Zn; remediation of trace elements is very likely; biological activities; antitumor activity; interferon-activity; immunosuppressive effects; anticoagulant activity; hypo-cholesterolemic activity; other medical uses have been reported	[123,163,182,183,184]
*Turbinaria conoides*	Sargassaceae	Alginic acid, alginate, laminaran, fucan complex contains monosaccharides, D-mannitol, fucosterol, antibiotic sarganin, antifungal activity, turbinaric acid	Antibacterial and antifungal activities, cytotoxic activity, herbivorous activity, possible phycoremediation activity of some heavy metals such as thulium, role in biosynthesis of nanoparticles	[123,185,186,187]

**Table 3:** List of Rhodophyta (red algae) species and the possible constituents and roles.

Species	Family	Main Constituents	Possible Roles	References
*Amphiroa fragilissima*	Corallinaceae	Cholesterol, non-protein amino acids; low-molecular-weight carbohydrates; floridoside, mannoglyceric acid; bioactive compounds such as ellagic acid, gallic acid, and phenolic compounds; major polyamines are found; trace elements are found such as Fe, Zn, Co, Pb, Mn, Cu, Al, etc.	Possible role of remediation in polluted water; ellagic acid may help prevent cancer cells from growing; gallic acid contains antioxidant, anti-inflammatory, and antineoplastic properties; phenolic compounds may have more roles: antitumoral, anticoagulant, antiviral, and hypocholesterolemic	[123,188,189]
*Centroceras calvulatum*	Ceramiaceae	Rich in protein, non-protein amino acids, fatty acids, cholesterol, rich in vitamin C	Might be non-conventional food and feed, possible remediation role in polluted sea water	[123,190]
*Ceramium luetzelbergii*	Ceramiaceae	Agar, some complex compounds containing monosaccharides are found, carotenoids, cholesterols, bromoperoxidase containing vanadium (V), trimethylamine, nitrate, choline, crystalline sulfur, Hg is found in some species of Ceramium	Possible indicator of Hg, possible universal monitor for heavy metals, antibacterial activity, antimitotic activity, agglutinin activity, folk medicine used for chest diseases	[123,191,192,193]
*Chondria armata*	Rhodomelaceae	Polysaccharides composed of mannose and galactose; xylogalactan sulphate; chondriol: a halogenated acetylene; volatile acids: sarganin and chonalgin; amino acids with some new amino acids; chondriamides; hemmagglutinins; cyclic polysuphides; some trace elements are found such as: Fe, Zn, Co, Pb, Mn, Cu, and Al	Possible role in remediation of heavy metals, antibiotic action, cytotoxic activity, activity against animal erythrocytes; other medical activities were recorded such as antitumor, antimicrobial, and antiviral effects	[60,123,194]
*Digenea simplex*	Rhodomelaceae	Agar contains: galactose, glucose, xylose, etc., agarose, sulphate ester; pectin analysis showed the presence of galactose, fructose, and arabonic acid, floridoside, digenic acid (kainic acid)	Some constituents can be used in medicine, food, and cosmetic industries; digenic acid is effective in expelling ascaris, possibly remediates heavy metals and organic compounds	[123,195,196]
*Hypnea* spp., (2 species): *H*. *cervicornis*, *H*. *valentiae*	Cystocloniaceae	Sulphated galactans; carotenoids such as α-carotene, β-carotene, lutein, and possibly others; peptidic agglutanins; phycolloid-containing ƙ-carrageenan; various forms of sterols and fatty acids; contains some elements such as Ca, Mg, K, Al, Fe, Mn, Cr, Ni, Cd, and Co	Food and animal feed, some agglutanins have agglutinating activity towards a variety of biological cells, including tumors, against human blood groups A, B, and O and animal erythrocytes; sulphated polysaccharides might have a role in supporting bones and may be used as anti-inflammatory agents and for other medical uses; pharmacological constituents could play various roles such as muscle relaxant, hypothermic activity, and phytoremediation of heavy metals such as Cd	[120,123,187,197,198]
*Jania* spp. (2 species were recorded): *J*. *adhaerens*, *J*. *ungulata*	Corallinaceae	Various carotenoids such as β-carotene, zeaxanthin, fucoxanthin, 9^~^-cis-fucoxanthin, fucoxanthinol, 9^~^-cis-fucoxanthinol, and epimeric mutatoxanthins; other organic compounds might be found; some heavy metals might be found	Phytoremediation of heavy metals is possible; not much information is available for some species; ameliorative effect on the toxicity of heavy some heavy metals for some animals and possibly humans	[123,199,200]
*Laurencia* spp. (6 species were recorded): *L*. *elata*, *L*. *glandulifera*, *L*. *intermedia*, *L*. *paniculata*, *L*. *papillosa*, *L*. *perforata*	Rhodymeniaceae	Various types of polysaccharides, sesquiterpenoides, diterpenoids, triterpenoids, other compounds such as C_15_-acetogensis, secondary metabolites such as sterols, fatty acids, amino acids, mineral elements: K, Na, Ca, Mg, Fe, Zn, Pb, Co, Cu, Mn, Al, possibly others: Cr, Ni, Cd, etc.	Various roles played by this macro-alga as food, medicine, numerous ecological roles *, refuge for marine organisms, hosts of various microorganisms and parasitic algae (such as *Janczewskia*); they are fed on by some grazers such as crabs, queen conch, and sea hares; possible roles in phycoremediation	[123,151,201]
*Polysiphonia* spp. (4 species were recorded): *P*. *brodiei*, *P*. *crassicolis*, *P*. *ferulacea*, *P*. *kampsaxii*	Rhodomelaceae	Sulphated galactans; polysaccharides belong to the agar class and agarose, other related residues such as mannitol and trehalose, etc., bromophenols, fatty acids, phospholipids, polar lipids, some structural components	Antibiotic (antibacterial and antifungal) and antioxidant activities; other roles such as antimitotic activity, increase survival of vorticellids; serum lipolytic activity; agglutinin; heavy metals are found such as As, Cd, Cr, Cu, Fe, Hg, Mn, Mo, Ni, Pb, Ti, V, and Zn; possible phycoremediation of petroleum hydrocarbons	[123,163,202,203]
*Spyridia filamentosa*	Callithamniaceae	Sterols such as cholesterol, fatty acids, and agglutinin are found in some species; main elements found are Al, Ca, Co, Cu, Fe, K, Mg, Mn, Na, Pb, and Zn	Antifungal activity of aqueous extracts, biosynthesis of silver nanoparticles, removing heavy metals from industrial wastewater	[123,204,205]
*Wurdemannia miniatat*	Solieriaceae	Little information is known about the chemical constituents (needs to be investigated)	No reported roles of this species	[123]

Tissues of some seaweeds accumulate heavy metals, particularly those found in the industrial wastewater of gas activities, such as As and Hg. Examples include *Acetabularia caliculus*, *Cladophora* spp. (green algae), *Sargassum* spp. (brown algae), and *Polysiphonia* spp. (red algae) (see Table 1, Table 2 and Table 3). Some other heavy metals found in industrial wastewater from oil and gas activities are found in many seaweeds as well, including Al, As, Ba, Cd, Co, Cr, Cu, Fe, Hg, Mn, Mo, Ni, Pb, V, and Zn [21,25,59,206].

Al-Thani and Yasseen [26] recently showed that heavy metals from industrial wastewater might accumulate in the tissue of marine animals or bind to the cell walls of algae, which could lead to negative impacts or disturbances in the food chain and human health. Therefore, monitoring the ecosystem in the Arabian Gulf could help to avoid or reduce the detrimental impacts of these trace elements on marine life. Some reported seaweeds in the Arabian Gulf have the ability to efficiently remediate heavy metals, including *Acetabularia caliculus*, *Avrainvillea amadelpha*, *Cladophora* spp., and *Ulva purtusa* (green algae); *Hormophysa cuneiformis* and *Sargassum* spp. (brown algae); and *Amphiroa fragilissima*, *Ceramium luetzelbergii*, *Chondria armata*, *Hypnea* spp., *Jania* spp., *Laurencia* spp., *Polysiphonia* spp., and *Spyridia filamentosa* (red algae). Organic components could be absorbed by seaweed biomass, biodegraded into smaller less toxic units, or metabolized [21,163,207].

Notably, microorganisms in seawater may be freely floating or are otherwise associated with or harbored by living marine organisms, including seaweeds. They might play significant roles in the degradation of organic compounds by converting them into smaller, less toxic units. Degradation of organic industrial wastewater can be achieved completely or partially (to less-toxic components) by microorganisms, particularly bacteria, that are sequestered in the vacuoles of seaweed tissues or involved in the metabolic pathways. Recent reports [21,22,26] have discussed the most common bacteria species found in the Arabian Gulf around the peninsula of Qatar that might play crucial roles in biodegradation and bio-stabilization of organic and inorganic components of industrial wastewater [163,208,209,210].

Seaweeds might also absorb extra essential elements such as nitrogen and phosphorus to avoid eutrophication in marine environments [211], which would lead to a loss of aquatic biodiversity, as well as a reduction in ecosystem services related to various important aspects of human life, such as fisheries, aquaculture, and recreation. Moreover, toxins released from some harmful algal blooms could have detrimental impacts on marine life and great negative consequences on human life.

Practical methods of phycoremediation using seaweeds include encouraging the cultivation of seaweeds that have proven efficient in removing toxic trace elements and metabolizing organic components of industrial wastewater (Table 1, Table 2 and Table 3). These seaweeds can be cultivated alongside fish and shellfish, which might help to improve the water quality and lead to healthier environments for aquaculture species. Construction of wetlands that involve seaweeds could improve the water quality by removing or stabilizing pollutants, and polluted seawater can be remediated under controlled conditions using seaweeds. They can be considered as bioreactors where contaminated water is treated and allow for the optimization of conditions for maximum pollutant removal [21,60,126,212].

Despite the success of practical methods of phycoremediation, some challenges and considerations should be considered. One of the most important issues is the monitoring the success of ecological restoration and maintaining a healthy environment [86]. Furthermore, the biomass of plants and seaweeds should be involved in various industrial and agro-biotechnological activities [213], such as manufacturing furniture, paper, domestic products, and food. They could also be used in composting, fertilizers, and transformation into less toxic forms and non-degradable complex molecules, as well as incineration for the chemical and electronics industries.

All the components need to be monitored and recycled safely to keep the toxic metals and any other dangerous components away from the food chain and to maintain the safety of ecosystems [21]. It is also important to spread awareness about the effects of environmental factors on remediation methods, such as temperature, salinity, light, and the presence of other pollutants [214]. Lastly, the introduction of edible plants and seaweeds to any remediation programs must be avoided to prevent any deterioration of the ecosystem, and monitoring in this regard should be involved in every step of the remediation [10].

## 7. Conclusions

The biological approach has emerged recently to solve pollution problems of industrial wastewater. It is an environmentally friendly solution for many issues facing the ecosystem and human life at various sectors of health, agriculture, and the economy. Recognizing the marine autotrophic organisms that have proven efficient in remediating polluted seawater is the first step in the right direction to speed up the process of removing or avoiding toxic components that result from oil and gas activities. Several plants, seaweeds, and microorganisms are promising candidates for remediation of the polluted seawater around the Arabian Gulf. Joint works are desperately needed to make the water of the Gulf suitable for marine life and humans. Such efforts are important in the context of increasing demands for energy components and with the great impacts of wars, conflicts, competition, and economic activities. Future research on sustainable ecological restoration in the Arabian Gulf should concentrate on the following aspects: (a) encouraging cultivation of autotropic living organisms reported in this article, (b) recognizing more autotrophs to boost the efforts for more active remediation of industrial wastewater, (c) establishing a continuous monitoring system of the seawater and the biomass of these marine organisms to set up a database and determine the possible threat of pollution at the ecosystem, (d) engaging the biomass of these living organisms with agro-biotechnology and recycling their components in various industrial activities, and (e) conducting modern bio-technological research like genetic engineering and tissue culture to develop transgenic marine autotrophs to boost the efforts of remediation methods.

## Supplementary Materials

The following supporting information can be downloaded at: https://www.mdpi.com/article/10.3390/toxics12090625/s1, Figure S1: A cable network of breathing roots (pneumatophores) that support the growth of plants in this stressful habitat; Figure S2: Salt glands are the main features of *Avicennia marina* that play vital role in ion regulation and homeostasis [94]; Figure S3: SEM of the adaxial surface (upper side) of leaves of *Avicennia marina*, scattered salt crystals are found (with magnification of X1200); Table S1: Analysis of heavy metals in industrial and anthropogenic wastewater. These compounds were determined during a joint project between Qatar University and Exxonmobil company (2012–2014), and a project sponsored by ESC (QU); Table S2: Analysis of petroleum hydrocarbons in industrial wastewater (mean of four replicates ± standard deviation); Table S3: Concentration of selected pollutants with elevated levels in relation to coastal and marine pollution [130]; Table S4: Mean values of physical and chemical features and heavy metals in the studied locations of the Arabian Gulf shore sediments and water [215].
