# Supplementary material for: Methods Using Marine Aquatic Photoautotrophs along the Qatari Coastline to Remediate Oil and Gas Industrial Water
Source: Toxics. 2024 Aug 24;12(9):625. doi: 10.3390/toxics12090625 (PMC11435476; doi:10.3390/toxics12090625)
Supplement: Supplementary file 1 [file toxics-12-00625-s001.zip › Figure S1.pdf]

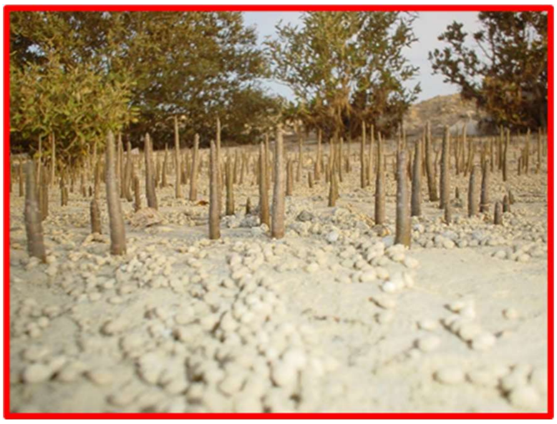

Supplementary Figure 1. A cable network of breathing roots (pneumatophores) that support the growth of plants in this stressful habitat.
