# Supplementary material for: Methods Using Marine Aquatic Photoautotrophs along the Qatari Coastline to Remediate Oil and Gas Industrial Water
Source: Toxics. 2024 Aug 24;12(9):625. doi: 10.3390/toxics12090625 (PMC11435476; doi:10.3390/toxics12090625)
Supplement: Supplementary file 1 [file toxics-12-00625-s001.zip › Figure S2.pdf]

J Coast Conserv (2008) 12:35–42
DOI 10.1007/s11852-008-0021-z

Ecophysiology of *Limonium axillare* and *Avicennia marina* from the coastline of Arabian Gulf-Qatar

B. T. Yasseen · M. A. Abu-Al-Basal

Received: 15 July 2008 / Revised: 27 August 2008 / Accepted: 27 August 2008 / Published online: 23 September 2008
© Springer Science + Business Media B.V. 2008

Abstract Attention is being focused on the coastline from Doha to Ras Laffan in Qatar since higher activities in the development of land and establishment of roads, highways and new buildings and houses is not coupled by serious studies on habitat destruction, fragmentation or disturbances. Ecophysiological study was carried out to investigate the adaptation of two halophytes (*Limonium axillare* and *Avicennia marina*) in this area, with special emphasis on the ultrastructure of salt glands found in the leaves. Soils in these locations accumulated much Na^+ and Cl^- as compared to other cations like K^+, Ca^{2+} and Mg^{2+}. Both plants accumulated higher concentrations of Na^+ , Cl^-, and Ca^{2+} and lower concentrations of K^+ and Mg^{2+}. Organic compounds found in leaves of these plants under their natural habitats including proline, soluble sugars and nitrogen, and photosynthetic pigments were determined. Scanning electron micrographs of the surface of leaves showed that salt glands of these plants are well developed. It is urgently required that exact vegetation maps, and monitoring exercises will be conducted, in order to document exactly the state of the vegetation in Qatar. Only this will allow the environment authorities to bring forward suggestions for vegetation and ecosystem management to the decision makers.

B. T. Yasseen (✉)
Department of Biological Sciences, Faculty of Science,
Qatar University,
Doha, Qatar
e-mail: bassam_tahaa@yahoo.co.uk

B. T. Yasseen · M. A. Abu-Al-Basal
Department of Biological Sciences, Faculty of Science,
Al-al-Bayt University,
Almafraq, Jordan

M. A. Abu-Al-Basal
e-mail: m_basal@yahoo.com

Keywords *Avicennia marina* · Ecophysiology · Halophytes · *Limonium axillare* · Salt glands

Introduction

The coastline from Doha to Ras Laffan is not only a repository for natural gases but also has a wealth of wild plants. Gas companies have pledged to the Supreme Council for Environmental Nature Reserve (SCENR) to implement Environmental Impact Assessment (EIA) for any plan to change the natural habitat (Abdel-Bari and Yasseen 2004; Yasseen and Al-Thani 2007). The land wildlife in this area is facing serious threat if higher activities in land development and establishment of new industrial constructions are not coupled by any studies on habitat destruction, fragmentation or disturbances. There is fear that before knowledge is obtained on the flora and fauna and their ecophysiological aspects, natural habitats will be lost. Studies on the flora and vegetation of the State of Qatar did not cover aspects of the ecophysiology of plants living in the different local habitats (Abulfatih et al. 2001; Böer and Al-Hajiri 2002).

Halophyte plants are normally living near the coastal line and through inland sabkhas; have two main mechanisms to cope with saline environment; these are avoidance mechanisms and tolerance mechanisms. Avoidance by exclusion mechanisms involves structural with physiological adaptations to minimize salt concentrations in the cells, or physiological exclusion by root membranes. This is an effective mean in removing the majority of salt from seawater, and red mangrove is an example of a salt-excluding species by this method (Adam 1993). Also, salt exclusion may occur at the cellular levels, and salt resistance can be attributed to the maintenance of ions homeostasis in the cytoplasm (Glenn 1997). Salt extrusion is another avoidance mechanism, and is

an active process to excrete extra salts from leaves of many halophytes, and some specialized structures called salt glands are found in leaves of these plants to regulate extra salts inside the plant body (Orcutt and Nilsen 2000). Some halophytes dilute the accumulated ions in plant tissues to keep the cytoplasmic salinity below toxic levels (Dilution mechanism). These plants are considered as salt includers (Adam 1993). Tolerance mechanisms of these plants include mainly osmoregulation (Levitt 1980; Orcutt and Nilsen 2000; Larcher 2003).

The present study focuses on the ecophysiological aspects and the adaptation of two halophytes (*Limonium axillari* and *Avicennia marina*) in the coastline of Arabian Gulf-Qatar, with special emphasis on the ultrastructure of salt glands found in the leaves.

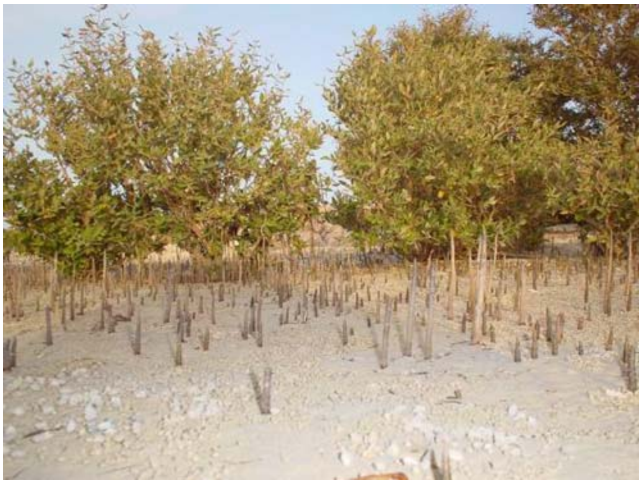

Fig. 2 Mangrove (*Avicennia marina*) with respiratory roots

The experimental works

Description of plants

Limonium is a member of the family Plumbaginaceae. It is a halophyte undershrub plant. In the flora of Qatar it is represented by one species (*Limonium axillare*). It is widespread on sabkhas and coastline and usually associated with some other halophytes like *Halopeplis*. The main feature of the soil occupied by *Limonium* is salt flat with compact sandy soil, sabkhas and undulating sandy mounds. It is a low woody shrub with fleshy leaves. Leaves are oblanceolate, crassulate, large with distinct salt glands, usually basal. Inflorescences handsome much branched; branches of the inflorescences in a tiered-pagoda pattern (Fig. 1). Flowers minute appearing rose, pink, deep magenta and white at maturity. *Limonium* flowers from December to May.

Avicennia belongs to the family Avicenniaceae (Verbenaceae) which is a family of mangroves common in warm waters such as the Arabian Gulf and the Red Sea. The family is locally represented by one genus (*Avicennia*) to which *A. marina* (Forssk.) Vierh., the only mangrove species in Qatari waters belongs. *A. marina* is internationally known as the Grey Mangrove. Evergreen dark green mangrove trees or shrubs not exceeding 4 m high producing a cable network of breathing roots (pneumatophores), each root 40–60 cm long, corky and covered with lenticels (Fig. 2). Leaves opposite, shiny green, glabrous above, grey and hairy beneath, ovate to ovate-lanceolate. Inflorescences are terminal and axillary clusters of small yellow flowers. Fruit 1–1.5 cm across; seeds viviparous germinating in the fruit on the mother plant and exposing 2 very large fleshy cotyledons. Mangrove forests are common on the muddy shorelines of the eastern coast of Qatar (Abdel-Bari et al. 2007)

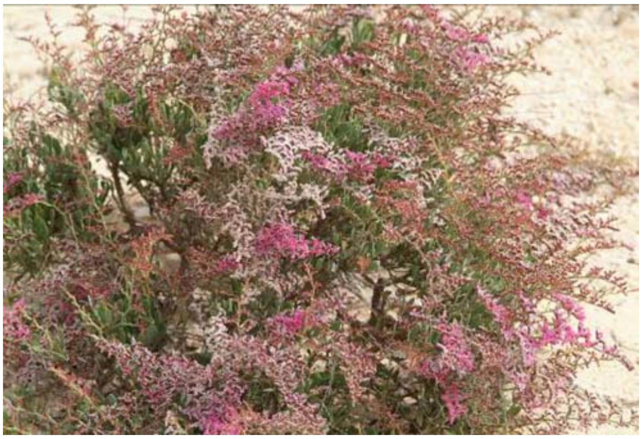

Fig. 1 Branches of the inflorescences in *Limonium axillare*

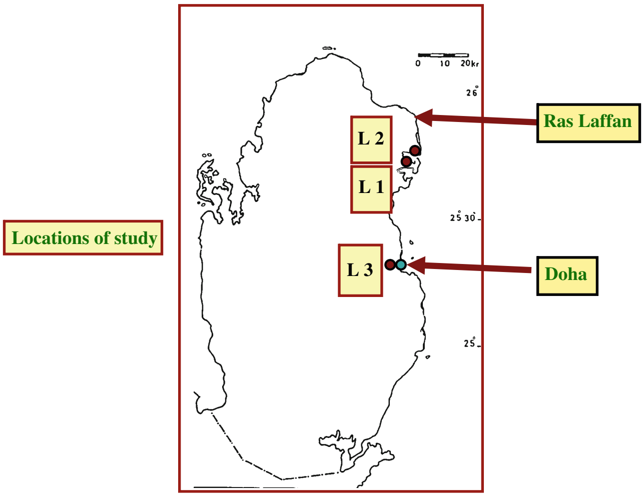

Fig. 3 A map of Qatar showing the study locations

Methods

The study locations

Three main locations around Doha and at the eastern coast of the State of Qatar were studied as shown in Fig. 3. These locations were designated as L1 (Ras-Almatbakh), L2 (Al-Dhakhira), and L3 (East of the Doha Golf Club). Samples of the plant and soil were collected from these locations for different types of analysis needed. The field surveys commenced on September 2001 and continued throughout the growth season till May 2002.

Chemical composition of soils and plants

Soil samples were collected from the root zone of the selected plants and transported to the laboratory. The physical and chemical properties of soil including water content, soil texture, pH of soil extract, ECe, mineral content and the field capacity were studied (EC; dSm^{-1}) using a Conductivity Meter, Jenway, 4200; pH using pH Meter, Jenway, 3305; field capacity as, %).

The concentration of major elements (Potassium, K^+; Calcium, Ca^{2+}; and Magnesium, Mg^{2+}) and Sodium, Na^+, in the water soil extracts was determined using an Atomic Absorption Spectrophotometer (Model Analyst 700, Perkin Elmer). Chloride, Cl^- was determined according to the method described elsewhere (Chapman and Pratt 1961).

Measurements of some of the chemical composition were done on shoots or leaves of both plants. These measurements include the following:

(1) Ionic composition: wet digestion of the ground oven dry samples with concentrated nitric acid was used to prepare solutions for the determination of Na^+, K^+, Ca^{2+}, Mg^{2+}. Atomic absorption spectrophotometer (Model analyst 700, Perkin Elmer) was used to determine those cations. Cl^- was determined in plant materials according to the method mentioned above.
(2) Proline: proline was determined according to the method described previously (Bates et al. 1973), using fresh materials.
(3) Soluble sugars: 100 mg of oven dry plant material was extracted with 5 ml of borate buffer, and left for 24 h, then centrifuged and filtered. The filtrate was used for the determination of direct reducing value (DRV) which includes all free monosaccharides, and total reducing value (TRV) which include all soluble sugars (Alhadi et al. 1999).
(4) Total soluble nitrogen (TSN): 1 ml of borate extract was digested using 50% sulphuric acid followed by 2–3 drops or more of 35% perchloric acid for complete oxidation. The mixture was made up to a 5 ml volume. Then total soluble nitrogen was determined in terms of ammonia (Alhadi et al. 1999).
(5) Photosynthetic pigments: chlorophylls a and b as well as carotenoids were determined according to the procedure described and used by many authors (Metzner et al. 1965; Abdel-Bari et al. 2007).

Scanning electron microscopy

Samples were prepared by the Electron Microscopy Unit at Qatar University, using Philips XL-30 Scanning Electron Microscope". The plant sample was dehydrated in a series of graded alcohol (50% alcohol, 70% alcohol, 90% alcohol and two changes in absolute alcohol). Critical point drying was done in a "Poloron Jumbo CPD". The samples were mounted on aluminum stub with a double sided carbon tape and then sputter coated with gold in an "Edwards Sputter Coater S150B" machine for 1 min to cover the specimen with a 40 Angstrom units thick gold layer. The specimens on the mounting stubs were inserted in the chamber of Philips XL-30 Scanning Electron Microscope and viewed at 20–30 Kv acceleration voltage. Photomicrographs were captured using a Polaroid film camera attached to the microscope.

Results and discussion

Ion regulation in plants under salt stress is well demonstrated in halophytes. These plants are able to grow normally under saline environment. In the present study, *Limonium axillare* occupies saline soils of a wide range of salinity; of ECe between 12 to above 200 dSm^{-1} (Table 1). *Avicennia marina*, on the other hand, prefers saturated saline soils, but they can grow in less salty environment (Downton 1982). Some species have been kept in pots where they had grown and flowered regularly when given

Table 1 Physical properties of the soil samples collected from different sites of the studied locations

The studied location	Soil texture	Absolute water content (%)	pH (soil extract)	ECe (dSm^{-1})
L1	Sandy-silty loam	2.5–27.8	6.5–7.7	12–49
L2	Sandy loam	12.9–26.1	8.2–8.4	>200
L3	Sandy-sandy loam	7.5–22.0	6.5–8.2	57–185

Table 2 The concentration of soluble elements in the water extracts of the soil samples collected from different locations of the studied soils

The studied location	mg/l of soil extract				
	Na^+	K^+	Ca^{2+}	Mg^{2+}	Cl^{-a}
L1	202–392	88–118	59–80	44–87	6–62
L2	511–814	96–97	81–97	40–83	575–714
L3	383–480	86–115	74–97	53–87	68–437

[a] g/l of soil extracts

only fresh water (Tomlinson 1986; Hogarth 1999). This species is the local mangrove species and is widespread on the Arabian Gulf coastline, on the coastline of some Gulf islands, and in Red sea coastline (Abdel-Bari et al. 2007; Yasseen and Al-Thani 2007). Plants such as mangroves living in sea waters tend to develop aerial roots with lenticels for gaseous exchange. Soils in these locations accumulated much Na^+ and Cl^- as compared to other cations like K^+, Ca^{2+}, and Mg^{2+} (Table 2).

Adaptation of plants to salinity may be associated with different mechanisms of uptake, translocation, accumulation and utilization of ions present in high concentrations in the root environment. Table 3 showed clearly that both plants accumulated higher concentrations of Na^+, Cl^-, and Ca^{2+} and lower concentrations of K^+ and Mg^{2+}. It seems that these plants had absorbed the most abundant ions in their growth medium (Table 2), those ions being involved in various roles in the physiology and biochemistry of plants under saline environment. However, some previous reports on halophytes have stressed that Na^+, Cl^-, and K^+ accumulate to achieve osmotic adjustment by lowering solute potential and water potential in plant tissues (Downton 1982; Flowers and Yeo 1986).

The accumulated Ca^{2+} in the plants studied could play various functions under saline environment like: (1) maintaining the internal membrane structure exposed to high concentrations of Na^+ and Cl^-, (2) regulating ion homeostasis by affecting ion channels and antiporters. Ca^{2+} blocks the uptake of K^+ into guard cells leading to stomatal closure as an adaptation mechanism under salt stress. It is quite possible that such mechanism might be operating in both plants, (3) increasing the plasticity of tissues, and (4) controlling cell membrane permeability (Nilsen and Orcutt 1996; Orcutt and Nilsen 2000; Taiz and Zeiger 2006). On the other hand, Mg^{2+} concentration in those plants was lower than that recommended by previous reports for normal growth (Chapman and Pratt 1961). Mg^{2+} is an important element occupying a central position in the chlorophyll molecule, thereby affecting the concentration of chlorophylls a and b in the green plant tissues (Yasseen and Al-Thani 2007). In fact, the results of the present study revealed that the photosynthetic pigments contents were less than those reported for many glycophytes (Bengtson et al. 1987; Alhadi et al. 1999; Yasseen 2001). Na^+ and Cl^- may interfere with the accumulation of macronutrients, by inhibiting H^+-ATPase of plasma membranes and consequently inhibit the protein transporters leading to nutrient imbalance (Fukuda et al. 1998). Moreover, these ions may be sequestered in vacuoles leaving relatively low ion content in the cytoplasm. Organic solutes like proline, soluble sugars and glycinebetaine could accumulate in the cytoplasm to contribute in the osmotic equilibrium across the tonoplast (Greenway and Munns 1980; Flowers 1985; Hasegawa et al. 2000).

Studies for the last five decades have suggested that the accumulation of proline in tissues confers some salinity tolerance in different plant species including halophytes (Orcutt and Nilsen 2000; Kavi Kishor et al. 2005). However, the plants under investigation, from different locations, exhibited different abilities to accumulate proline, and the content of proline showed wide variations (Youssef

Table 3 Shoot chemical composition of *Avicennia marina* and *Limonium axillare*

Variables	*Avicennia marina*	*Limonium axillare*
Na^+ (mgg^{-1} dry wt.)	18–51[a]	61–149
K^+ (mgg^{-1} dry wt.)	2.5–4.4	5.2–12.5
Ca^{+2}(mgg^{-1} dry wt.)	45–52	19–60
Mg^{+2} (mgg^{-1} dry wt.)	0.43–0.55	0.25–4.23
Cl^- (mgg^{-1} dry wt.)	42–55	16–35
Proline (μgg^{-1}fresh wt.)	16–43	120–920
Total soluble sugars (mgg^{-1}DW)	2.2–6.0	4.7–8.0
Total soluble nitrogen (μgg^{-1}DW)	40–63	67–169
Chlorophyl A (μgg^{-1}fresh wt.)	390–508 (441)[b]	150–202 (172)
Chlorophyl B (μgg^{-1}fresh wt.)	190–256 (226)	75–95 (81)
Carotenoids (μgg^{-1}fresh wt.)	125–158 (142)	55–75 (64)
Total Photosynthetic pigments (μgg^{-1}fresh wt.)	809	317

[a] The number of observations varied from 6 to 12 from different locations

[b] Figures between parentheses are means of the observations

et al. 2003); much proline was found in *Limonium axillare*, while very low proline concentrations were found in the shoot of *Avicennia marina*. Such variation can be attributed to various factors such as: (a) different metabolic responses of these plants due to the environmental factors facing them in their habitats, (b) the energy constraints that may result in a substantial amount of energy required for active transport and the extrusion of ions (Yeo 1983). In fact the debate about the actual role of proline in the metabolism of plants under stress has been a subject of many studies during the last three decades with no decisive conclusions (Chen and Murata 2002; Abdel-Bari et al. 2007). However, many reports have indicated that glycinebetaine could be a main compatible osmolyte accumulates in high concentrations to conifer halophytes the adaptability to harsh saline environments (Paleg and Aspinall 1981; Rhodes and Hanson 1993; Youssef et al. 2003). Such role is being evaluated in many plants using new approaches (Ashraf and Foolad 2007). Soluble nitrogen contents were considerably lower than those for soluble sugars in the shoot system of these plants. Qatari soils have been reported to be poor in nitrates particularly sabkha soils (Ashore 1991). The low nitrogen content in sandy soils of the locations investigated might explain the reduction of available soluble nitrogen in plant tissues. Moreover, it has been shown that the carbon skeletons of soluble nitrogen compounds may be converted to organic acids by a series of biochemical reactions leading to soluble sugars such as sucrose (Alhadi et al. 1997; Taiz and Zeiger 2006).

Both plants differ in their content in the photosynthetic pigments (Table 3), and were less than those reported for many glycophytes (Alhadi et al. 1999). The reduction in photosynthetic pigments was attributed to the inhibition of the biosynthesis of pigments under saline environments (Levitt 1980).

Salt glands

Both plants developed excretory system in the shoot systems to rid the plant of excess salts. These structures are called salt glands; these are common in salt tolerant plants like *Avicennia* and *Limonium*. Visible salts crystals can be seen easily on the leaves of *Limmonium* and at the gland itself (Figs. 4 and 5).

Figures 6, 7, and 8 showed scanning electron micrographs of the surface of leaves of *Limonium axillare*. The stomata and salt glands appeared well organized. Moreover, *Limonium* seemed to have some kind of succulence which could play a role in dilution of salts absorbed by this plant (Adam 1993).

Avicennia marina plants, on the other hand, have leaves grey and hairy at the lower surface where most secretion occurs (Fig. 9), while the above surface is shiny green, glabrous with some salt glands (Figs. 10, 11, and 12).

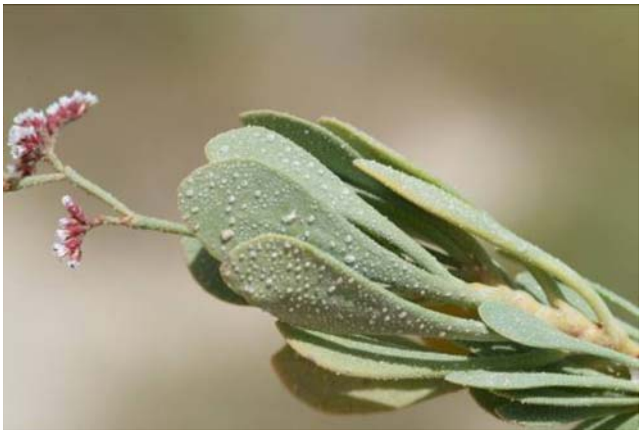

Fig. 4 Salt crystals on the lower surface of leaves of *Limonium axillare*

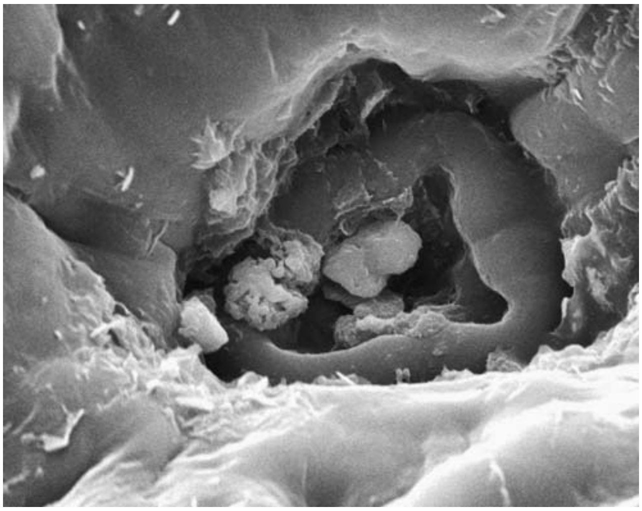

Fig. 5 Salt crystals appear in the mouth of a salt gland of *Limonium axillare*. Magnification ×1,450

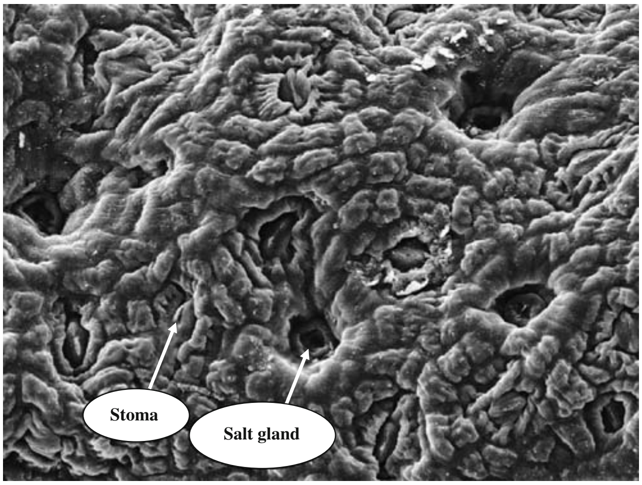

Fig. 6 The lower leaf surface of *Limonium axillare* showing stomata and salt glands. Magnification ×250

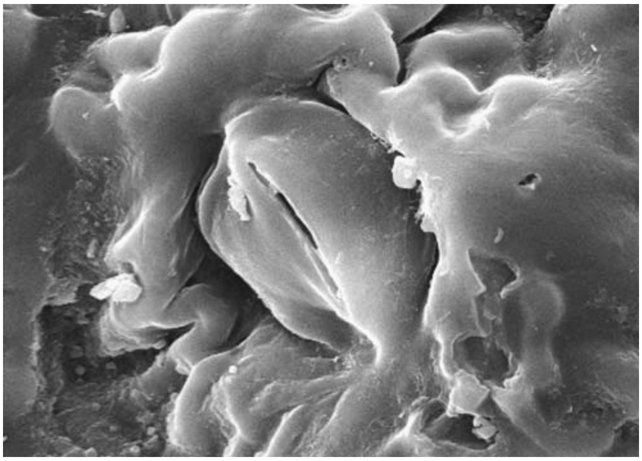

Fig. 7 A half open stoma from a leaf of *Limonium axillare*. Magnification, ×1,250

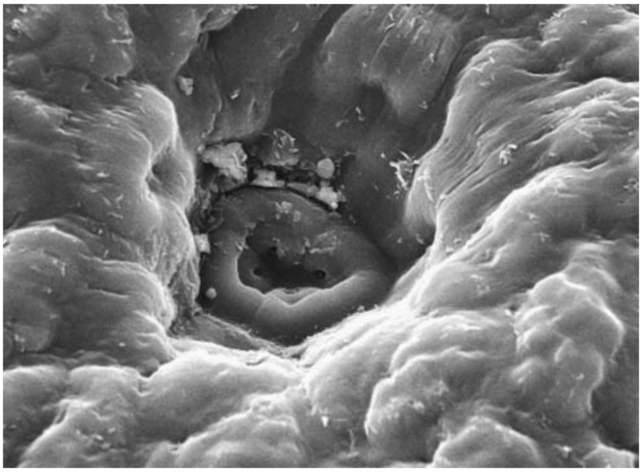

Fig. 8 A salt gland with four holes and some salt crystals in *Limonium axillare*. Magnification ×1,250

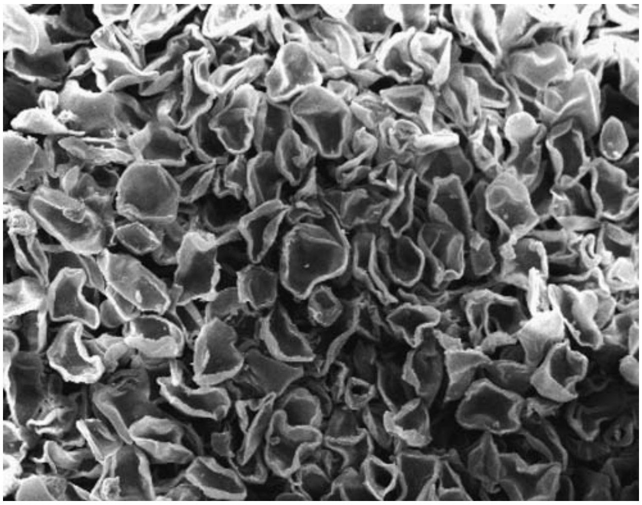

Fig. 9 The lower surface of *Avicennia marina* showing the hairy surface. Magnification ×250

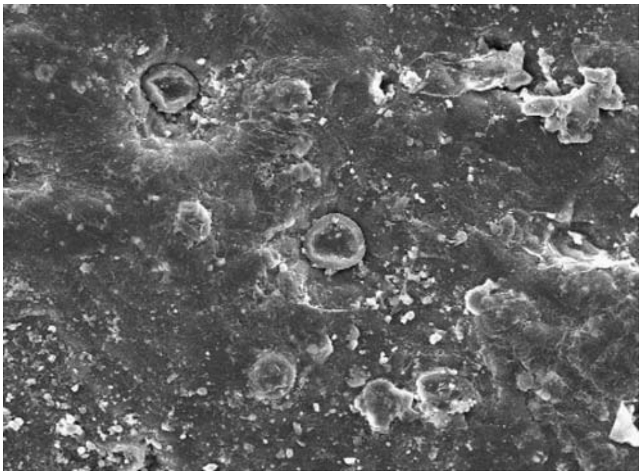

Fig. 10 The upper surface of *Avicennia marina* leaf showing some salt glands. Magnification ×250

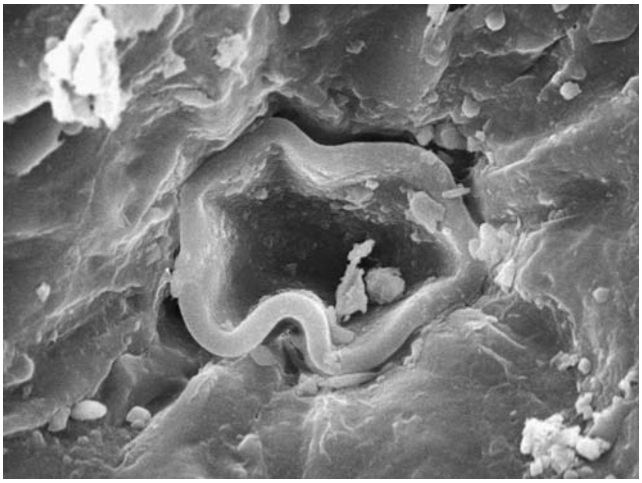

Fig. 11 A salt gland from the upper surface of *Avicennia marina* leaf. Magnification ×1,350

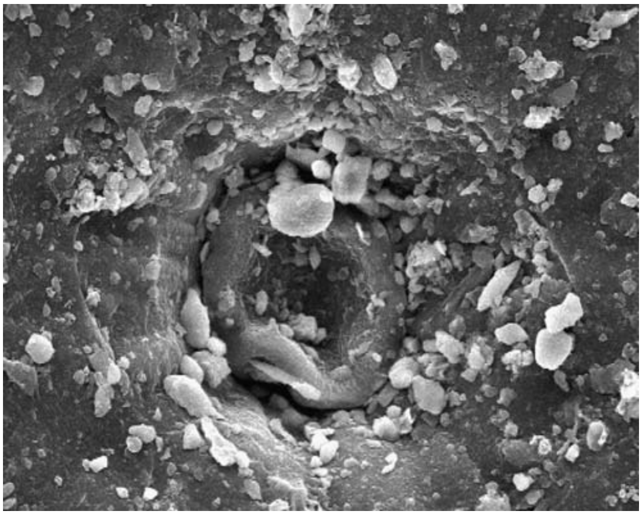

Fig. 12 A salt gland from the upper surface of *Avicennia marina* leaf, showing salt crystals around the gland. Magnification ×1,200

It is reasonable to accept that these plants have adaptive characteristics, which contribute to their avoidance of high concentrations of ions in their leaves. Salt glands are the main features of *Limonium* and *Avicennia* that play vital role in ion regulation and homeostasis (Abdel-Bari et al. 2007).

Figures 5 and 11 showed the structure of these salt glands; playing significant role in extrusion of Na^+ and Cl^- ions which are appeared as crystals in the mouth of the salt glands. It has been reported that salt glands found in *Limonium* and in other halophytes, that the transition to active Cl^- transport was accompanied by the displacement of vacuoles toward the cell periphery and by the establishment of plasmalemma contact sites with tonoplast which appeared similar to gap junctions in animal epithelial cells. It is suggested that gland vacuoles may have a primary role in Cl^- secretion and that the tonoplast may be functionally asymmetrical, so that the free part facing the hyaloplasm bears ion pumps, whereas highly permeable ion channels are active along the zone of contact with the plasmalemma (Vassilyev and Stepanova 1990).

Finally, these halophytes deserve special attention for several reasons; in addition to their economic and medical importance (Saenger 2002; Al-Easa et al. 2003), they could offer unique genetic pools to be used for gene technology programs (Flowers 2004), and keeping in mind they are the sole representatives of the families they belong in the flora of Qatar (Abdel-Bari et al. 2007). Thus, it would be very useful to provide educational material to improve the awareness about their habitats, ecophysiology, importance as well as threats facing them and conservation and management strategies (Saenger 2002). Moreover, recent reports (Yasseen and Al-Thani 2007; Richer 2008) have stated that the industrial development in the state of Qatar is putting the environment at risk, threatening ecosystem services and biological diversity. Therefore, it is urgently required that exact vegetation maps, and monitoring exercises will be conducted, in order to document exactly the state of the vegetation in Qatar. Only this will allow the environment authorities to bring forward suggestions for vegetation and ecosystem management to the decision makers (Abdel-Bari et al. 2007).

Acknowledgment The authors would like to thank Environmental Studies Center, Qatar University, for the financial support, and Al-al-Bayt University for facilitating publishing this work.

References

Abdel-Bari EM Yasseen BT (2004) Restoration of a coastline vegetation. Ras Laffan Company, sponsored by SARC, University of Qatar, Doha, Qatar

Abdel-Bari EM Yasseen BT, Al-Thani RF (2007) Halophytes in the State of Qatar. Environmental Studies Center. University of Qatar, Doha, Qatar

Abulfatih HA, Abdel-Bari EM, Alsubaey A, Ibrahim YM (2001) Vegetation of Qatar. Scientific and Applied Research Center (SARC), University of Qatar, Doha, Qatar

Adam P (1993) Saltmarsh ecology. Cambridge University Press

Al-Easa HS, Rizk, AM, Abdel-Bari EM (2003) Chemical constituents and nutritive values of range plants in Qatar. Scientific and Applied research Center, University of Qatar, Doha, Qatar

Alhadi FA, Yasseen BT, Al-Dubaie AS (1997) Changes in carbohydrate and nitrogen fractions during germination of fenugreek (*Trigonella foenum-graecum* L.) seeds presoaked in GA_3, growing under different osmotic potentials. Qatar Univ Sci J 17:271–279

Alhadi FA, Yasseen BT, Jabr M (1999) Water stress and gibberellic acid effects on growth of fenugreek plants. Irrig Sci 18:185–190, doi:10.1007/s002710050061

Ashore MM (1991) Sabkhas in the peninsula of Qatar – geomorphologic and geological and biological studies – University of Qatar. Centre of Documentation and Humanitarian Studies, Doha, Qatar

Ashraf M, Foolad MR (2007) Roles of glycinebetaine and proline in improving plant abiotic resistance. Environ Exp Bot 59(2):206–216, doi:10.1016/j.envexpbot.2005.12.006

Bates LS, Wadleigh RP, Teare ID (1973) Rapid determination of free proline for water-stress studies. Plant Soil 39:205–207, doi:10.1007/BF00018060

Bengtson C, Klockare B, Klockare R, Larsson S, Sundqvist C (1987) The after-effect of water stress on chlorophyll formation during greening and the level of abscisic acid and proline in dark grown wheat seedlings. Physiol Plant 43:205–212, doi:10.1111/j.1399-3054.1978.tb02565.x

Böer B, Al-Hajiri S (2002) The coastal and sabkha flora of Qatar: An introduction. In: Barth HJ, Böer B (eds) Sabkha ecosystems vol.: the Arabian peninsula and adjacent countries. Tasks for vegetation science 36. Kluwer Academic Publishers, The Netherlands, pp 63–70

Chapman HD, Pratt PF (1961) Methods of analysis for soils, plants and waters. Div Agr Sci Univ Calif Berkeley, California

Chen TH, Murata N (2002) Enhancement of tolerance of abiotic stress by metabolic engineering of betaines and other compatible solutes. Curr Opin Plant Biol 5(3):250–257, doi:10.1016/S1369-5266(02)00255-8

Downton WJS (1982) Growth and osmotic relations of the mangrove (*Avicennia marina*) as influenced by salinity. Aust J Plant Physiol 9(5):519–528

Flowers TJ (1985) Physiology of halophytes. Plant Soil 89:41–56, doi:10.1007/BF02182232

Flowers TJ (2004) Improving crop salt tolerance. J Exp Bot 55 (396):307–319, doi:10.1093/jxb/erh003

Flowers TJ, Yeo AR (1986) Ion relations of plants under drought and salinity. Aust J Plant Physiol 13:75–91

Fukuda A, Yazaki YT, Ishikawa S, Koike T, Tanaka Y (1998) Na^+/H^+ antiporter in tonoplast vesicles from rice roots. Plant Cell Physiol 39:196–201

Glenn EP (1997) Mechanisms of salt tolerance in higher plants. In: Basra AS, Nasra RK (eds) Mechanisms of environmental stress resistance in plants. Harwood Academic Publishers, Amsterdam, pp 83–110

Greenway H, Munns R (1980) Mechanisms of salt tolerance in nonhalophytes. Annu Rev Plant Physiol 31:149–190, doi:10.1146/annurev.pp.31.060180.001053

Hasegawa PM, Bressan RA, Zhu J-K, Bohnert HJ (2000) Plant cellular and molecular responses to high salinity. Annu Rev Plant Physiol Plant Mol Biol 51:463–499, doi:10.1146/annurev.arplant.51.1.463

Hogarth PJ (1999) The biology of mangroves. Oxford University Press, New York

Kavi Kishor PB, Sangam S, Amrutha RN, Sri Laxmi P, Naidu KR, Rao KRSS, Rao S, Reddy KJ, Theriappan P, Sreenivasulu N (2005) Regulation of proline biosynthesis, degradation, uptake and transport in higher plants: its implications in plant growth and abiotic stress tolerance. Curr Sci 88(3):424–438

Larcher W (2003) Physiological plant ecology. Eco-physiology and stress physiology of functional groups, 4th edn. Springer, Berlin

Levitt J (1980) Responses of plants to environmental stresses. II. Water, radiation, salt and other stresses, 2nd edn. Academic, New York

Metzner H, Rau H, Senger H (1965) Untersuchungen zur synchronisierbarkeit einzelner pigmentmangel – mutanten von *Chlorella*. Planta 65:186–194, doi:10.1007/BF00384998

Nilsen ET, Orcutt DM (1996) Physiology of plants under stress. Abiotic factors. Wiley, New York

Orcutt DM, Nilsen ET (2000) Physiology of plants under stress. Soil and biotic factors. Wiley, New York

Paleg LG, Aspinall D (1981) The physiology and biochemistry of drought resistance in plants. Academic, Sydney

Rhodes D, Hanson AD (1993) Quaternary ammonium and tertiary sulfonium compounds in higher plants. Annu Rev Plant Physiol Plant Mol Biol 44:357–384, doi:10.1146/annurev.pp.44.060193. 002041

Richer R (2008) Conservation in Qatar: Impacts of Increasing Industrialization. Center for International and Regional Studies (CIRS). Georgetown University, School of Foreign Service in Qatar

Saenger P (2002) Mangrove Ecology, Silverculture and Conservation. Kluwer Academic Publishers, Dordrecht

Taiz L, Zeiger E (2006) Plant physiology. Redwood City, California

Tomlinson PB (1986) The botany of mangrove. Cambridge University Press, New York

Vassilyev AE, Stepanova AA (1990) The ultrastructure of ion-secreting and non-secreting salt glands of *Limonium platyphyllum*. J Exp Bot 41:41–46, doi:10.1093/jxb/41.1.41

Yasseen BT (2001) Preliminary assessment of the effect of saline water used in irrigation on the growth of the local barley cultivar (Harma). Qatar Univ Sci J 21:55–64

Yasseen BT, Al-Thani RF (2007) Halophytes and associated properties of natural soils in the Doha area, Qatar. AEHMS 10:320–326

Yeo AR (1983) Salinity resistance: physiologies and prices. Physiol Plant 58:214–222, doi:10.1111/j.1399-3054.1983.tb04172.x

Youssef AM, Hassanein RA, Hassanein AA, Morsy AA (2003) Changes in quaternary ammonium compounds, proline and protein profiles of certain halophytic plants under different habitat conditions. Pak J Biol Sci 6(10):867–882
