# Supplementary material for: Methods Using Marine Aquatic Photoautotrophs along the Qatari Coastline to Remediate Oil and Gas Industrial Water
Source: Toxics. 2024 Aug 24;12(9):625. doi: 10.3390/toxics12090625 (PMC11435476; doi:10.3390/toxics12090625)
Supplement: Supplementary file 1 [file toxics-12-00625-s001.zip › Table S1.pdf]

Supplementary Tabe 1A. Analysis of heavy metals in industrial wastewater. These compounds were determined during a joint project between Qatar University and Exxonmobil company (2012-2014).

Element	Concentration ±S.D.	Element	Concentration ±S.D.
Essential elements		Non-essential elements	
K	20.97±1.52	As	00.00±00.00
Mg	11.98±0.60	Ba	36.36±3.41
P	0.123±6.43	Cd	0.65±0.04
Co	0.51±0.07	Cr	8.51±4.00
Cu	119.80±13.25	Hg	No data
Fe	3937.0±247.6	Li	448.83±31.08
Mn	158.73±9.25	Pb	00.00±00.00
Mo	1.86±0.32	Se	No data
Na	263400.0±17367.5	V	00.00±00.00
Ni	2.32±0.11	Note: Some important heavy metals such as Hg and Se that normally found in oil and gas components were not measured.	
Zn	2898.33±177.16		

Four replicates for each treatment, K, P, and Mg (PPM), all the remaining elements PPB. More data can be obtained from Exxonmobil company, Research Qatar Limited.

.

Supplementary Table 1B. Analysis of heavy metals in wastewater of anthropogenic origin in a untreated pond near Doha, the capital of Qatar. These data are among the report of Sweileh (2002) [see reference [21] These figures might change depending on the source and time.

Trace element	Range of concentrations over one year (µg/L),
Cr	1.55-7.62
Cu	Trace -10.90
Mn	32.2-169.0
Ni	No detected-2.02
Pb	No detected-6.67
