# Supplementary material for: Methods Using Marine Aquatic Photoautotrophs along the Qatari Coastline to Remediate Oil and Gas Industrial Water
Source: Toxics. 2024 Aug 24;12(9):625. doi: 10.3390/toxics12090625 (PMC11435476; doi:10.3390/toxics12090625)
Supplement: Supplementary file 1 [file toxics-12-00625-s001.zip › Table S2.pdf]

Supplementary Tabel 2A. Analysis of petroleum hydrocarbons in industrial wastewater (mean of four replicates ± standard deviation). These compounds were determined during a joint project between Qatar University and Exxonmobil company (2014). Anthropogenic compounds were determined by Al-Naimi (2002) [see reference 21]. These figures might change depending on the source and time.

Petroleum hydrocarbons (oil and gas)		
TPH (PPM*)	PAH (PPB*)	Oil & Grease (PPM)
11.35 ±3.83	3.83±1.04	42.75±20.17

Anthropogenic compounds*	
PCBs (ppm)	TOM (μg/L)
Ranged from ND - 111.57(the figures covered 12 months)	Ranged from 0.90-70.80<br>16.27±19.63 (Average of 12 months)

PPM: Part per Million, PPB: Part per Billion, TPH: Total Petroleum Hydrocarbons, a large group of chemical compounds that come from crude oil, PAH: Polycyclic Aromatic Hydrocarbons that are naturally present in crude oil, gas, and other energy components. Anthropogenic compounds: substances created by humans for industrial agriculture, and other commercial purposes. PCB: Polychlorinated-Biphenyls, TOM: Total Organic Matter. ND: Not detected.

Supplementary Tabel 2B. The physical properties of the wastewater as compared to distilled and tap water.

Type of water	Color	pH	EC ($mScm^{-1}$)	TDS (g/ l)	Salinity (ppt)
Distilled water	Colorless	6.2	0.14	0.1216	0.1
Tap water	Colorless	7.2	0.27	0.2165	0.1
Wastewater	Dark grey	5.3	4.31	3.2600	2.2

N.B. Wastewater was brought in March 2013 from Ras Laffan, Qatar. ppt; part per thousand.

Documents of ESC, Qatar University.
