# Supplementary material for: Methods Using Marine Aquatic Photoautotrophs along the Qatari Coastline to Remediate Oil and Gas Industrial Water
Source: Toxics. 2024 Aug 24;12(9):625. doi: 10.3390/toxics12090625 (PMC11435476; doi:10.3390/toxics12090625)
Supplement: Supplementary file 1 [file toxics-12-00625-s001.zip › Table S3,.pdf]

Journal of the Association of Arab Universities for Basic and Applied Sciences (2015) **17**, 90–100

University of Bahrain
Journal of the Association of Arab Universities for Basic and Applied Sciences
www.elsevier.com/locate/jaaubas
www.sciencedirect.com

REVIEW ARTICLE

Heavy metal, trace element and petroleum hydrocarbon pollution in the Arabian Gulf: Review

Afnan Mahmood Freije *

Department of Biology, College of Science, University of Bahrain, Sakhir Campus, P.O. Box 32038, Bahrain

Received 30 June 2013; revised 30 December 2013; accepted 11 February 2014
Available online 12 March 2014

KEYWORDS
Air;
Arabian Gulf;
Heavy metals;
Hydrocarbons;
Marine;
Soil;
Pollution

Abstract The Arabian Gulf environmental status was assessed based on studies conducted in Bahrain, Kuwait, Oman, Saudi Arabia, Qatar, and United Arab Emirates (UAE) during 1983–2011. This review examines all sorts of pollutions in the Arabian Gulf area over the last three decades. Approximately 50 published studies were reviewed in order to determine the pollution status in the Arabian Gulf regarding heavy metals and organic substances. Three types of environmental pollutions including marine and coastal, soil, and air were addressed in this review as well as sources of pollutants and their effect on biological systems, marine organisms, and human health. Emphasis is placed on marine pollution, particularly toxic metal, and petroleum hydrocarbon contaminations. Major parts of this review discuss the consequences of the 1991 Gulf War on the environment, and the substantial changes associated with the marine habitats. The effects of oil field fires in Kuwait following the 1991 Gulf War were evaluated through studies that investigated hydrocarbons concentration and trace metals in samples of near shore sediments, bivalves, and fish collected from Kuwait, Saudi Arabia, Bahrain, UAE, and Oman. Total petroleum hydrocarbons (TPH) and polycyclic aromatic hydrocarbons (PAHs) were discussed in biota (fish and various bivalves) and coastal sediments from six countries in the Gulf. The review has revealed different concentrations of pollutants, low, moderately, and chronically contaminated areas from oil and metals. It has also outlined effective sustainable management measures and goals as a first step in the evaluation of coastal, marine, soil, and air environment in the Arabian Gulf area.
© 2014 Production and hosting by Elsevier B.V. on behalf of University of Bahrain.

1. Introduction

Many species function in the Arabian Gulf close to physiological limits (Sheppard, 1993), and thus, added stress imposed by diverse input of pollutants into the marine environment (e.g. terminals, tanker accidents and spills due to wars, offshore oil exploration and land based industrial and urban sources, recreational and agricultural development) is likely to have severe consequences (Madany et al., 1995; Al-Saleh et al., 1999; Sheppard et al., 2010). It is well known that seafood in the Arabian Gulf including fish and shrimp is of value for both local consumption and export revenue. Therefore, maintaining good marine environmental quality is crucial for several socio-economic reasons (Price et al., 1993; Sadiq et al., 2002; Sheppard et al., 2010). Furthermore, sea water quality issues are of extreme importance due to the fact that many of the

* Tel.: +973 17437418; fax: +973 17449158.
E-mail address: afreije@uob.edu.bh
Peer review under responsibility of University of Bahrain.

1815-3852 © 2014 Production and hosting by Elsevier B.V. on behalf of University of Bahrain.
http://dx.doi.org/10.1016/j.jaubas.2014.02.001

Gulf countries depend on desalinated seawater as a source of potable water for domestic and industrial use.

Water, air, and soil contamination by organic and inorganic pollutants is a major issue in the countries of the Arabian Gulf. The Arabian Gulf is an extension of the Indian Ocean located in the southwest of Asia between the Islamic Republic of Iran and the Arabian Peninsula (UN, 2006). The peninsula is made up of seven countries; Bahrain, Kuwait, Oman, Saudi Arabia, Qatar, United Arab Emirates (UAE), and Yemen. Six countries in the list above in addition to Iraq and Iran accounts for around one-fourth of the world's oil production (Khan, 2002). The Arabian Gulf is relatively shallow with an average depth of 35 m; rarely exceeding 100 m. It is a semi-enclosed body of water that only connects to the open waters through the Strait of Hormuz (Reynolds, 1993; Sheppard, 1993; Massoud et al., 1996; Khan, 2002). The Arabian Gulf contains a variety of relatively fragile ecosystems that are associated with an environment that is naturally highly stressful with very high evaporation rates, poor flushing characteristics, elevated temperatures, salinity and UV exposure. Therefore, contaminants are more likely to undergo limited dilution, slower dispersion, and residing in the area for a longer time (Madany et al., 1996a; de Mora et al., 2004).

The Arabian Gulf was the scene of three wars during the last three decades; the Iraq–Iran War in 1980–1988, the first and second Gulf War in 1991and 2003 respectively. As a result, the Arabian Gulf was subjected to a massive oil spill in 1991 in which 6–8 million barrels of Kuwait crude oil were released in the Arabian Gulf as well as various spills from normal oil operation and tanker-related spills (Kureishy, 1993; Literathy, 1993; Madany et al., 1996a; Sheppard et al., 2010). The oil spill associated with the 1991 Gulf War was considered the largest oil spill in the history. Therefore, large number of studies focused on the fate of this spill and provided evidence that the oil spill effect was limited to 400 km from the spillage point to Saudi Arabian coastline and that the main contaminants were rapidly degraded (de Mora et al., 2010).

Although, the division of pollution into categories such as air, water, land etc. has not been favored by some scientists who believe that every pollutant tends to end up in the ocean (William, 1996), the following review is divided into three sections: (1) marine and coastal pollution, (2) soil pollution, and (3) air pollution. Marine, soil and air contaminations will be the focus of this review which aims at identifying all sorts of pollutants, their sources, levels, and distributions in sediments, soil, organisms, and air during the last three decades. It is important to find out the degree of contaminations and its effect on marine life, fisheries, and human health. The present work examines 30 years of monitoring data in the Arabian Gulf for metals and petroleum hydrocarbons providing invaluable baseline information for future assessment.

2. Marine and coastal pollution

Heavy metal contamination in the marine environment of the Arabian Gulf was investigated in wide varieties of marine organisms. The effect of the Gulf War oil spill in 1991 on the concentration of heavy metals in marine organisms was the highlights of several studies. The concentrations of Cu, Pb, Zn, Cd, Fe, Mn, and Ni were determined in the tissues of the grouper fish *Epinephelus coioides* from different areas in the Arabian Gulf including UAE, Oman, Kuwait, and Bahrain from 1991 to 1996 (Fowler et al., 1993; Habashi et al., 1993; Al-Sayed et al., 1996; Madany et al., 1996a). Levels of all heavy metals investigated were within internationally accepted levels except for Pb and Zn that were in the higher side of these limits in Al-Sayed et al. study (1996) (Tables 1 and 2). Kureishy (1993) also investigated the contribution of the 1991 oil spill to heavy metal contamination in marine organisms around Qatar. This study concluded that no significant increase in the concentrations of Hg, Cd, Pb, Cu, Co or Ni was recorded in the muscle tissue of various benthic and semipelagic species studied.

The effects of oil field fires in Kuwait following the 1991 Gulf War were investigated by determining hydrocarbons concentration and trace metals in samples of near shore sediments, bivalves, and fish collected from Kuwait, Saudi Arabia, Bahrain, UAE, and Oman (Fowler et al., 1993). This survey revealed that hydrocarbon contamination was restricted to ~400 km from Kuwait with the highest contamination levels along the coast of Saudi Arabia between Ras Al Khafji and Ras Al Ghar. However, the study also indicated that much of the oil in the intertidal zone had degraded within few months of the spill and that the concentrations of petroleum hydrocarbons and trace metals in sediments and bivalves outside the immediate area of impact were as low as or even lower than those levels in samples collected from the same site before the war.

The levels of Cu, Zn, Pb, Mn, Ni, Cd, and Fe in seawater and the pearl oyster *Pincatada radiata* from two locations around Bahrain were determined by Al-Sayed et al. (1994) between March 1991 and March 1992. Metal concentrations were higher in oysters in comparison to seawater; however, they were within the World Health Organization (WHO) limits except for Pb and Cd (Tables 1 and 2).

Marine pollution in the territorial water of the kingdom of Bahrain was assessed by analyzing heavy metals As, Cd, Cu, Fe, Mn, Ni, Pb, V, Zn, and Hg in seawaters from 23 different sites known as fishing areas in the year 2007 (Juma and Al-Madany, 2008). The authors concluded that the concentrations of the elements studied were within the United Kingdom Quality Standards (UK standards) (As 25 $\mu g\,l^{-1}$, Cd 5 $\mu g\,l^{-1}$, Cu 5 $\mu g\,l^{-1}$, Fe 1000 $\mu g\,l^{-1}$, Ni 30 $\mu g\,l^{-1}$, Pb 25 $\mu g\,l^{-1}$, V 100 $\mu g\,l^{-1}$, Zn 40 $\mu g\,l^{-1}$, and Hg 0.3 $\mu g\,l^{-1}$) and the United States Environmental Protection Agency (USEPA) (As 36 $\mu g\,l^{-1}$, Cd 8.8 $\mu g\,l^{-1}$, Cu 3.1 $\mu g\,l^{-1}$, Fe 300 $\mu g\,l^{-1}$, Mn 50 $\mu g\,l^{-1}$, Ni 610 $\mu g\,l^{-1}$, Pb 8.1 $\mu g\,l^{-1}$, Zn 81 $\mu g\,l^{-1}$, and Hg 0.94 $\mu g\,l^{-1}$) recommended water quality criteria except for copper in all sites (4.53–119 $\mu g\,l^{-1}$) and mercury (0.38 $\mu g\,l^{-1}$) in one site and that the Kingdom of Bahrain's marine waters is of good water quality (Tables 1 and 2).

The metals (Cd, Co, Cr, Mo, Ni, Pb, V, and Zn) were measured in the clam (*Meretrix meretrix*), seawater, and sediments were collected from five stations on the Saudi coast during the period April–May 1991, after the 1991 Gulf war (Sadiq and McCain, 1993). The researchers compared metal concentrations in their study with those collected from the same stations in the same period in 1985. They concluded that metal concentrations in clams collected during 1985 and 1991 were similar in some stations but reduced or increased in others. However, the magnitude of increase was much greater in clams collected in 1991 from stations located toward the north (i.e. toward

Table 1 Concentrations of selected pollutants with elevated levels in relation to coastal and marine pollution.

Country	Media	Concentrations reported	Source
Saudi Arabia	Coastal beaches	Tar (1–10 kg m^{-1})	Coles and Gunay (1989)
Saudi Arabia	Sediments	TPH (62–1400 μg g^{-1} dry wt.)	Fowler et al. (1993)
	Clams	TPH (570–2600 μg g^{-1} dry wt.)	
	Fish	TPH (9.6–31 μg g^{-1} dry wt.)	
Bahrain	Pearl oyster	Pb (1.25–14.0 μg g^{-1} wet wt.),<br>Cd (0.25–3.8 μg g^{-1} wet wt.)	Al-Sayed et al. (1994)
Arabian Gulf	Bottom sediments	Fe (353–32,150 μg /g dry wt.)	Al-Abdali et al. (1996)
Bahrain	Fish	Pb (4.3–15.2 μg g^{-1}wet wt.),<br>Zn (223–1253 wet wt.)	Al-Sayed et al. (1996)
Bahrain, Qatar, UAE	Bottom sediments	TPH (50–1122 μg g^{-1} dry wt.)	Massoud et al. (1996)
Bahrain	Costal sediments	Pb (mean, 111 mg/kg dry wt.)	Akhter and Al-Jowder (1997)
Kuwait	Fish	T-Hg (⩾0.5 mg/kg dry wt.)	Al-Majed and Preston (2000)
Bahrain	Sediments	TBT (mean, 40 ng Sng^{-1} dry wt.),<br>DBT (mean, 30 ng Sng^{-1} dry wt.),<br>MBT (mean, 10 ng Sng^{-1} dry wt.)	de Mora et al. (2003)
Qatar	Sediments	TBT (mean, 1.7 ng Sng^{-1} dry wt.),<br>MBT (mean, 3.1 ng Sng^{-1} dry wt.)	
Oman	Sediments	TBT (mean, 60 ng Sng^{-1} dry wt.),<br>MBT (mean, 9.7 ng Sng^{-1} dry wt.)	
Bahrain	Pearl oyster	Zn (mean, 4290 μg g^{-1} dry wt.),<br>V (mean, 7.3 μg g^{-1} dry wt.),<br>Pb (mean, 3.92 μg g^{-1} dry wt.)	de Mora et al. (2004)
	Bivalves	As (meam, 153–156 μg g^{-1} dry wt.)	
	Sediments	Cu (mean, 48.3 μg g^{-1} dry wt.),<br>Hg (mean, 0.22 μg g^{-1} dry wt.),<br>Pb (mean, 99 μg g^{-1} dry wt.),<br>Zn (mean, 52.2 μg g^{-1} dry wt.)<br>Cd (109–195 μg g^{-1}dry wt.)	
Oman	Fish liver	As (mean, 9.6 μg g^{-1} dry wt.),	
UAE	Sediments	Co (mean, 45.2 μg g^{-1} dry wt.),<br>Cr (mean, 303 μg g^{-1} dry wt.),<br>Ni (mean, 1010 μg g^{-1} dry wt.)	
Bahrain	Sediments	TPH equiv. (mean, 779 μg g^{-1} dry wt.),<br>∑PAHs (mean, 6.6 μg g^{-1} dry wt.)	Tolosa et al. (2005)
Oman	Rock oyster	TPH equiv. (mean, 99 μg g^{-1} dry wt.),<br>∑PAHs (mean, 173 μg g^{-1} dry wt.)	
UAE	Sediments	TPH equiv. (73–100 μg g^{-1} dry wt.),<br>∑PAHs (6.5–9.4 μg g^{-1} dry wt.)	
UAE	Rock oyster	TPH equiv. (mean, 290 μg g^{-1} dry wt.),<br>∑PAHs (846 μg g^{-1} dry wt.)	
UAE	Pearl oyster	TPH equiv. (mean, 35 μg g^{-1} dry wt.),<br>∑PAHs (mean, 251 μg g^{-1} dry wt.)	
Oman	Abalone	Cd (11–30 mg kg^{-1} dry wt.)	Fowler et al. (2007)
Bahrain	Water	Cu (4.53–119 μg l^{-1}),<br>Hg (mean, 0.38 μg l^{-1})	Juma and Al-Madany (2008)
Bahrain	Sediments	TOC (mean, 4.78%),<br>TPH (mean, 1850 μg g^{-1} dry wt.)	de Mora et al. (2010)
Oman	Oyster	TPH (mean, 572 μg g^{-1} dry wt.)	
Bahrain	Sediments	Cd (mean, 19.14 mg kg^{-1})	Naser (2010)

TPH: total petroleum hydrocarbon, TOC: total organic carbon, TBT: tributyltin, DBT: dibutyltin, MBT: monobutyltin, PAHs: polcyclic aromatic hydrocarbons.

Kuwait) suggesting that oil spill probably affected metal accumulation in northern stations in comparison with other stations.

The effect of the 1991 Gulf war on the Saudi prawn (*Penaeus semisulcatus*) stocks was preliminary assessed by Mathews et al. (1993). Saudi prawn landings fell markedly from nearly 4000 *t* in 1989 to ~25 *t* in the first half of 1992. In addition egg/larval abundance on the spawning grounds was about an order of magnitude lower in 1992 in comparison to earlier years (Price et al., 1993). However, it was impossible to identify the exact cause of prawn stock collapse since the collapse could have been caused by several factors one of which was the Gulf war which appeared to be the most likely cause of the stock failure (Mathews et al., 1993).

The concentration of heavy metals (Cd, Pb, Ni, V, and As) in four fish species: Emperors (*Lethrinus miniatus*), Greasy-grouper (*Epinephelus tauvina*), Rabbitfish (*Siganus canaliculatus*), and Doublebar-bream (*Acanthopagrus bifasciatus*) from

Table 2 Number of studies reviewed reporting elevated levels of metals and petroleum hydrocarbons in relation to coastal and marine, soil and air pollution from 1983 to 2010 by country.

Country	Coastal and marine pollution	Soil pollution	Air pollution
Bahrain	10	3	7
Kuwait	1	–	–
Oman	5	–	–
Qatar	2	–	–
Saudi Arabia	1	1	1
UAE	3	–	–
Arabian Gulf	1	–	–

three selected sites with agricultural, municipal, and industrial activities along the coast of the Eastern Province of Saudi Arabia were determined (Al-Saleh and Shinwari, 2002). The levels of studied metals: cadmium 0.07 μg/day, lead 0.13 μg/day, and arsenic 0.12 μg/day were below the maximum fish human consumption permissible level allowed by international legislations for cadmium, lead, and arsenic of 0.06, 0.21, and 0.12 mg/day respectively for 60 kg adult and by Saudi legislation for cadmium and lead of 0.0067–0.0083 mg/kg body weight respectively, and for arsenic daily intake of 0.002 mg/kg body weight. However, nickel and vanadium levels were close or below the detection limits ($<$ 0.5 and 0.11 μg/g wet weight) (WHO, 1993; Al-Saleh and Shinwari, 2002).

An assessment study of heavy metal concentration in marine biota (fish and various bivalves) from the Gulf and Gulf of Oman during 2000–2001 was conducted by de Mora et al. (2004). The authors investigated heavy metals in two economically important fish species, the orange spotted grouper (*E. coioides*, hamour) and the spangled emperor (*Lethrinus nebulosus*, sheiry) in which very high Cd concentration was found in their liver and was attributed to food-chain bioaccumulation due to upwelling in the region. On the hand, certain bivalve species (*P. radiata* and *Saccostrea cucullata*) had very high concentrations of As in which the authors attributed to natural origins rather than anthropogenic contamination (Tables 1 and 2).

Petroleum hydrocarbon, organochlorinated compound and heavy metal concentration in benthic marine organisms were surveyed in 1983 and 20 years later in 2002 at the same location in Dhofar, southern Oman in order to assess major changes in contaminant levels (Fowler et al., 2007). The authors concluded that levels of petroleum residues in 2002 in several species indicated low to moderate chronic oil contamination but not higher than levels measured in 1983. Total Petroleum hydrocarbons (PHs), chlorinated hydrocarbons, and the sum of polychlorinated biphenyl ($\sum$-PCB), and heavy metal (Hg, Cu, Cd, Pb, and V) levels were low in both surveys, however *p,p′*-dichlorodiphenyldichloroethylene (pp′-DDE) increased in 2002 most likely due to the prior usage of dichloro diphenyl trichloroethane (DDT). Nevertheless Cd levels in commercially valuable abalone (*Haliotis mariae*) (11–30 mg kg^{-1} dry weight) exceeded the recommended limits for shellfish (1 mg kg^{-1} dry weight) (FAO, 1983) and 4 mg kg^{-1} dry weight for mollusks (EC, 2001) (Tables 1 and 2). Toxic metals were also measured in 10 commercial marine fish species collected from seafood factories in Oman and proven to be within safety limits recommended by various organizations such as the European Union for hazardous metals (EC), WHO, and the United States Food and drug administration (EC, 2001; USFDA, 1993a,b,c; WHO, 1993) and do not pose health risk (Al-Busaidi et al., 2011).

Total petroleum hydrocarbons (TPH) and polcyclic aromatic hydrocarbons (PAHs) were investigated in biota and coastal sediments from Bahrain, Qatar, UAE, and Oman (Tolosa et al., 2005). In comparison to worldwide reports such as those reported by the National Status and Trends (NS&T) sites in Alaska, and the United Nations Environment Programme (UNEP) (UNEP, 1991), TPH and PAHs were relatively low in sediments and biota studied except for sediments collected near the BAPCO oil refinery in Bahrain in which TPH concentrations were 779 μg g^{-1} equivalents and 6.6 μg g^{-1} $\sum$PAHs that can be categorized as chronically contaminated. Sediments and bivalves around Akkah Head and Abu Dhabi in the UAE and near Mirbat in Oman showed some evidence of oil contamination (Tables 1 and 2).

de Mora et al. (2010) also studied various petroleum hydrocarbons (PHs) and organochlorinated contaminations in marine biota (fish and various bivalves) and coastal sediments from seven countries in the Gulf and Gulf of Oman. Apart from all sites studied, BAPCO oil refinery site (Bahrain) was the only one considered as chronically contaminated (Tables 1 and 2). Total organic carbon (TOC) measured in surface sediments was low and ranged from 0.05% to 1% in all areas studied except the sediment from Dukhan, Qatar (2.34%) and BAPCO refinery area (4.78%). The concentration of total petroleum hydrocarbon (TPH) was extremely high (1850 μg g^{-1}) in sediments collected offshore from BAPCO refinery as they were in 2000 (Tolosa et al., 2005) indicating a chronically contaminated site. The TPH concentrations in bivalves ranged from 0.9 to 572 μg g^{-1} dry weight being highest (572 μg g^{-1}) in rock oysters from Mina Al Fahal in Oman reflecting chronic contamination attributed by the authors to oil terminal and refinery plant at that site.

Arsenic levels were measured by Bu-Olayan and Thomas (2001) in seawater, microplankton (diatoms and dinoflagellates), shrimp (*Penaeus semisulcatus*), mollusk (*Cerithium scabridum*), and five types of fish Maid (*Liza macrolepis*), Nakroor (*Pomadasys argenteus*), Nuwaiby (*Otolithes argenteus*), Suboor (*Hilsha ilisha*), and Sheim (*Acanthopagrus latus*) in five sampling stations off Kuwait coast during the period from 1995 to 1999. In their study the authors concluded that most of the marine organisms studied have low arsenic levels in comparison to other parts of the globe.

Mercury content was analyzed in shrimp (*Penaeus semisulcatus*) and five types of fish Emperors (*L. miniatus*), Greasy-grouper (*E. tauvina*), Rabbitfish (*S. canaliculatus*),

and Doublebar-bream (*A. bifasciatus*) from three locations along the coast of the Eastern Province of Saudi Arabia in 1998 (Al-Saleh and Doush, 2002). The mercury levels they reported were lower than the limits proposed by Saudi legislation and the United States Food and Drug Administration (FDA or USFDA) in fish and shellfish of 1 μg/g (USFDA, 1979; SASO, 1997) and lower than the Japanese and Brazilian legislation maximum permissible limit of 04 and 0.5 μg/g wet wt. respectively (Nakagawa et al., 1997; Kehrig et al., 1998).

The heavy metals Cr, Cu, Zn, Ni, Cd, and Pb were determined in the edible part (mantle) of the cuttlefish *Sepia pharaonis*. Although this marine species is favored among Saudi Arabia population, it is captured from the Arabian Gulf water which is a well known area of anthropogenic and industrial pollution that receives urban effluents (Al Farraj et al., 2011). However, this study provided evidence regarding the levels of heavy metals studied being within the safe limits for human consumption based on the world and the legal standards that are set by international legalizations. The edible portions including skin of 20 popular fish species in Qatar were examined for Cu, Zn, Pb and Hg and proven to be safe for human consumption (Al-Jedah and Robinson, 2001). The levels of Pb, Zn, Cu, Cr, Cd and Ni were measured in the mantle of the common cuttlefish *S. pharaonis* from the Arabian Gulf and also found to be within safe limits and can be eaten with confidence (Al Farraj et al., 2011).

Total mercury (Hg) and methyl mercury (MeHg) content in fish tissue from the Arabian Gulf were assessed by several investigators from1983 till 2009 (Anderlini et al., 1983; Al-Hashimi and Al-Zorba, 1991; Khordagui and Al-Ajmi, 1991; Habashi et al., 1993; Madany et al., 1995; Al-Majed and Preston, 2000; de Mora et al., 2004; Freije and Awadh, 2009). In 1991 Khordagui and Al-Ajmi (1991) assessed the risk of mercury in Kuwaiti fish consumers and they reported that all of the fish and shrimp consumed in Kuwait were below the mercury acceptable daily intake limit. In addition, Al-Hashimi and Al-Zorba (1991) also determined Hg concentrations in the muscle of eight commercial fish from Kuwait which also were below ($\leqslant$0.91 mg/kg wet weight) the limit of 1 mg/kg (wet weight) set by USFDA (USFDA, 1979). Madany et al. (1995) reported that the overall mean of T–Hg in some fish from Bahrain was 0.084 mg/kg (wet weight) and the provisional tolerable weekly intake of total mercury was 0.45 μg/kg, also below the limit of 1 mg/kg (wet weight) set by USFDA (USFDA, 1979).

More recently, an assessment of the total mercury and methyl mercury content of zooplankton and fish tissue collected from Kuwait territorial waters was done by Al-Majed and Preston (2000). They have reported that 20.6% of the fish analyzed had $\geqslant$0.5 mg/kg total mercury, and exceeded the WHO limit which is 0.5 mg/kg for T–Hg and 0.3 mg/kg for MeHg, while 20.6% had $\geqslant$0.3 mg/kg of MeHg (Tables 1 and 2). The study estimated that 3.2 kg of Hg and 1.9 kg of MeHg are being removed yearly by fish landings from Kuwait territorial waters and introduced to the local food supply. de Mora et al. (2004) determined the mercury levels in fish of the Gulf and Gulf of Oman and found that total mercury levels in top predator fish commonly consumed in the region were < 0.5 mg/kg and posed no threat to public health. The latest study on Hg and MeHg concentrations in four fish types from Bahrain conducted by Freije and Awadh (2009) showed that concentrations for both T–Hg (0.022–0.117 mg/kg wet weight) and MeHg (0.028–0.123 mg/kg wet weight) were the lowest in the Arabian Gulf region and much lower than the action level of Hg and MeHg in fish (0.5–1.0 mg/kg wet weight) (Al-Hashimi and Al-Zorba, 1991; USFDA, 2006).

Saudi Arabia Gulf beaches were surveyed for tar pollution over a period of 20 months from May 1985 to October 1986 (Coles and Gunay, 1989). Tar concentrations were commonly greater than 10 kg m^{-1} of shoreline while its frequently ranged from 1 to 10 kg m^{-1} (Tables 1 and 2). Tar values reported were 10 times higher than values reported elsewhere in the Arabian Gulf such as Bahrain (0.014–0.858 kg m^{-1}), Oman (0.001–0.906 kg m^{-1}) (Fowler, 1985), and Kuwait (0.005–2.33 kg m^{-1}) (Burns et al., 1982) and 100 times upper than values reported in other regions of the world such as Australia (0–0.055 kg m^{-1}), West Coast of India (0.005–0.033 kg m^{-1}), Kenya (0.019–0.09 kg m^{-1}), Netherland (0.002 kg m^{-1}), Newzealand (0 kg m^{-1}), and the United States (0.016–0.048 kg m^{-1}) (Oostdam, 1984). Author's attributed tar abundance to recent oil spills at that time rather than seasonal changes in oceanographic conditions.

Seventy-one core samples were collected in 1992 from the bottom sediments of the Arabian Gulf and their trace metal contents (Fe, V, Ni, Pb, and Cu) were determined and used as indicators of pollution levels in relation to the Kuwait oil slick occurred in 1991 (Al-Abdali et al., 1996). All trace metals studied were within permissible natural background levels (Zn 30–60, Pb 15–30, Cd 1.2–2.0, Ni 70–80, Mn, 300–600, Fe 10,000–20,000, V 20–30, and Cu 15–30 μg/g) in the western offshore area off Bahrain, Qatar, and the UAE with the exception of Fe contamination (Tables 1 and 2) in the northwest area off Kuwait which occurred due to major river transport on Shatt Al-Arab according to the researchers. This study concluded that Kuwait oil slick was deposited in limited coastal area between Kuwait and Bahrain and that Kuwait oil slick had minimal effect on trace metal pollution in the Arabian Gulf. In 1997, Akhter and Al-Jowder measured lead in the sediments of 19 coastal stations in Bahrain. They reported high levels of lead (111 mg/kg) (Tables 1 and 2) which attributed to pollution from land-based industrial and urban sources, namely automobiles that contribute to the overall pollution in the coastal area. Madany et al. (1996a) have also reported high lead levels in some local fish and shellfish in Bahrain (Tables 1 and 2).

Oil pollution in the Arabian Gulf in relation to the Kuwait oil slick was investigated in 1992 by measuring total petroleum hydrocarbon (TPH) and total organic carbon (TOC) in 77 core samples from the bottom sediments of the Arabian Gulf (Massoud et al., 1996). The investigators identified seven chronic moderately and heavily polluted areas in the region which they attributed to natural oil seepage, accidental pipelines damage, accidental tanker spillage, Nowruz oil slick, and deballasting tankers. Massoud et al. (1996) also identified 10 areas with intermediate and high pollution levels most probably affected by the Kuwait oil slick (Tables 1 and 2).

Organotin contamination in marine sediment and biota from the Gulf and adjacent region was assessed in 2003 by de Mora et al. Three regions in Qatar, Bahrain, and Oman were classified as contaminated areas regarding tributyltin (TBT) concentrations in sediments (Tables 1 and 2). However, organotin species were considered relatively low in fish and bivalves from the Gulf in comparison to global standards

(TBT $\leqslant$ 30 ng Sng^{-1} dry weight) and did not pose any immediate problems to public health (de Mora et al., 2003).

Heavy metals were also measured in coastal sediments in the Gulf and Gulf of Oman during 2000–2001 (de Mora et al., 2004). Concentrations of heavy metals were generally as low as they were in 1993 (Fowler et al., 1993) except for two hot spots; in Bahrain off the BAPCO (Bahrain Petroleum Company) refinery in which the levels of Cu, Hg, Pb, and Zn were elevated signaling localized anthropogenic inputs. The other hot spot was at Akkah Beach on the east coast of UAE where the concentrations of As, Co, Cr, and Ni were elevated reflecting the metal-rich mineralogy of the region (Tables 1 and 2) (de Mora et al., 2004). Elevated Cd levels (19.14 mg kg^{-1}) were also reported near a desalination plant off the eastern coastline of Bahrain and attributed to petroleum industries as well as effluents from a variety of factories and industrial facilities (Naser, 2010).

3. Soil pollution

The heavy metals Pb, Zn, Cd, Cr, and Ni were measured in both indoor house dust and outdoor street dust from different sites in Bahrain (Akhter and Madany, 1993; Madany et al., 1994). The levels of those toxic metals were elevated in all samples studied and were attributed mainly to automobile exhaust. In general, the higher levels of lead contamination were reported in the north and northeastern part of Bahrain where traffic is concentrated (Tables 2 and 3). The contents of street dust from different sites in Bahrain were analyzed for the elements Pb, Zn, Ni, Cu, and Cd. The authors attributed the major source of metals in street to automobile dust with high levels of Pb contamination specifically in traffic concentrated areas (Tables 2 and 3) (Madany et al., 1996b).

4. Air pollution

Air quality in Bahrain was monitored during the period November 1986–May 1987 from a station located in an industrial area located in North Sitra in the proximity of power and desalination plants as well as a petrochemical industry. The parameters H_2S, NO_x, NO, SO_2, O_3, CO, CH_4, and nonmethylated hydrocarbon (NMHC) were analyzed and compared to standards accepted by different countries according to the authors (Madany and Danish, 1988). The authors also concluded that the results of their study have shown that most pollutants studied were lower than world standard levels except for CH_4 and NMHC (Tables 2 and 4). However, the authors did neither mention in their study the world standard levels at that time, nor did they refer to acceptable levels in different countries.

Heavy metal air contamination in Bahrain was also investigated by Madany et al. (1990, 1994). Lead levels were measured in the leaves of five species of trees at 18 locations in Bahrain (Madany et al., 1990). Although, lead levels were higher in leaves from high traffic density areas than those from low traffic density (9–420 μg/g); they were low in comparison to reports on leaves of trees along busy roads in developed countries such as Canada (100–3000 μg/g) (Cannon and Bowles, 1962), France (50–400 μg/g) (Quinche et al., 1969), and Britain (100–700 μg/g) (Barness et al., 1976).

Lead was also measured in the air of one rural and one urban area in Bahrain and was correlated to human blood levels by Vreeland and Ekarath (1990). The highest concentration of lead was detected in the air of the urban area which correlated with the volume of traffic; however those levels did not exceed the internationally acceptable limits (< 25 μg/dl). On the other hand, the average blood lead concentrations in Bahrainis

Table 3 Concentrations of selected pollutants with elevated levels in relation to soil pollution.

Country	Media	Concentrations reported	Source
Bahrain	House dust	Pb, Zn, Cd, Ni, Cr (mean, 697.2, 151.8, 72, 125.6, 144.4 μg g^{-1})	Akhter and Madany (1993)
	Street dust	Pb, Zn, Cd, Ni, Cr (mean, 360, 64.4, 37, 110.2, 144.7 μg g^{-1})	
Bahrain	Indoor dust	Zn (mean, 202 mg kg^{-1})	Madany et al. (1994)
	Outdoor dust	Pb (mean, 742 mg kg^{-1})	
Bahrain	Street dust	Pb (8–2550 mg kg^{-1}), Zn (10–243 mg kg^{-1}, Cu (3–80 mg kg^{-1}), Ni (1–122 mg kg^{-1})	Madany et al. (1996)

Table 4 Concentrations of selected pollutants with elevated levels in relation to air pollution.

Country	Media	Concentrations reported	Source
Bahrain	Air	CH4 (mean, 3.7 μL L^{-1}),<br>NMHC (mean, 8.79 μL L^{-1})	Madany and Danish (1988)
Bahrain	Blood	Pb (mean, 15.38 μg dl^{-1})	Vreeland and Ekarath (1990)
Bahrain	Air	NO_2 (13–76 μg m^{-3})	Danish and Madany (1992)
Saudi Arabia	Air	Pb (282 ± 144 ng m^{-3})	Sadiq and Mian (1994a)
Bahrain	Deciduous teeth	Pb (0.1–60.8 mg g^{-1} dry wt.)	Al-Mahroos and Al-Saleh (1997)
Bahrain	Umbilical cord blood	Pb (> 15.00 μg dl^{-1})	Al-Mahroos and Al-Saleh (2000)
Bahrain	Air	PM_{10} (181.6 ± 168.7 μg m^{-3}),<br>$PM_{2.5}$(51.3 ± 38.0 μg m^{-3}),<br>NO_2(23.8 ± 10.6 ppb),<br>O3(42.5 ± 22.4 ppm)	Khamdan and Al Madany (2009)
Bahrain	Blood	Pb (15.3 ± 5.7 μg dl^{-1})	Freije and Dairi (2009)

(15.38 μg/dl) (Tables 2 and 4) were higher in comparison with the levels found in other countries.

In Bahrain also, a study on umbilical cord blood levels of newborns has shown high lead levels in comparison to nearby regions in which unleaded gasoline is used, such as Saudi Arabia (Tables 2 and 4) (Al-Mahroos and Al-Saleh, 2000). Lead levels in deciduous teeth of children of Bahrain also showed toxic concentrations in 35% of those studied (Tables 2 and 4) (Al-Mahroos and Al-Saleh, 1997). Madany et al. (1996b) have also reported high lead levels in ambient air in Bahrain (Tables 2 and 4). However, the results of the most recent study on blood lead levels in Bahrain have shown a significant difference in blood lead levels in adults between the years 2000 (15.3 ± 5.7 μg/dl) and 2008 (2.09 ± 1.27 μg/dl), mainly due to the use of unleaded gasoline (Freije, 2009; Freije and Dairi, 2009).

The quality of ambient air in the Kingdom of Bahrain during 2007 as well as the temporal and spatial variations was also investigated in five different sites (Tables 2 and 4) (Khamdan and Al Madany, 2009). The air pollutants toluene, NH_3, xylene, benzene, Particulate Matter 10 (PM_{10}), Particulate Matter 2.5 ($PM_{2.5}$), SO_2, H_2S, CO, O3 and NO_2 were monitored and showed clear intrinsic variation among sites in the following descending order toluene, NH_3, PM_{10}, xylene, benzene, $PM_{2.5}$, SO_2, H_2S, O_3, CO, and NO_2. $PM_{2.5}$ showed higher mean ranks in Oil Refinery, washing plants and brick factories, and congested traffic areas. PM_{10} and $PM_{2.5}$ had higher values in areas exposed and close to the Bahrain desert and to the Arabia mainland due to dust and sand storms; an important weather phenomena in the Arabian Gulf region. NO_2 showed higher mean rank in two sites where Oil Refinery, power station, and aluminum smelter are located. In addition, CO also showed higher mean rank in areas where power station was located as well as high traffic area. The study of Khamdan and Al Madany (2009) also concluded that the air pollutants NO_2, O_3, and SO_2 exceeded the standard and guideline values and O_3 levels were the highest in an area near the airport due to aircraft emission, while NO_2 the main precursor of O_3 showed the lowest concentration in this area. On the other hand, the elevated NH_3 concentrations were spotted in the monitoring stations near the sewage treatment plants, the sheep farms, and the municipal waste container areas. Benzene concentrations had the highest mean rank in the capital area which could be attributed to the intensity of traffic. The authors also conclude that the nonparametric Kruskal–Wallis (KW) statistical test has shown a higher mean rank for toluene and xylene in areas nearby industrial sources which coincided with the lowest benzene KW rank and therefore suggested industrial sources rather than car exhaust (Khamdan and Al Madany, 2009). On the other hand, high H_2S concentrations near oil refinery were attributed to the natural gas exploration or gas leakage from main pipeline in that area. NO_2 concentrations were also examined throughout Bahrain by Danish and Madany (1992) in which they concluded that NO_2 values were greater in areas that had high traffic in comparison with airport and industrial areas (Table 3).

The concentrations of Pb, Ti, and Zn in air particulates at Dhahran, Saudi Arabia were determined during and after Kuwait oil fires in 1991. No statistical differences in Pb, Ti, and Zn concentrations were found between 1991 and 1992 suggesting limited effect from the Kuwait oil fires on air particulate specifically Pb, Ti, and Zn in Dhahran (Sadiq and Mian, 1994a). However, the reason behind elevated lead levels (282 ± 144 ng/m^{-3} per year) (Table 3) was attributed by the authors to automobile emissions. Sadiq and Mian (1994b) also measured Ni, and V in air particulates at the same period and concluded that Kuwait oil fires may have contributed to the elevated levels of Ni, and V during April–July 1991 at Dhahran. However, those levels would not be considered as a health concern at Dhahran during Kuwait oil fires.

The effect of Kuwaiti oil fires in 1991 on air quality in the Eastern Province of Saudi Arabia was also investigated by Amin and Husain (1994). Various air pollutants such as SO_2, CO, H_2S, and NO_2 measured in ambient air during the period of study (March–November 1991) were much lower (SO_2 (100–458 $\mu g/m^3/h$), CO (3335–3795 $\mu g/m^3/h$), H_2S (21–195 $\mu g/m^3/h$), and NO_2 (71–222 $\mu g/m^3/h$)) than the permissible limits set by the Meteorology and Environmental Protection Agency (MEPA): SO_2 (730 $\mu g/m^3/h$), CO (40,000 $\mu g/m^3/h$), H_2S (195 $\mu g/m^3/h$), and NO_2 (660 $\mu g/m^3/h$) (MEPA 1402H. Those values, however, compared with the values of previous years increased persistently during the period of oil well fires but still without exceeding the MEPA permissible limits.

5. Major pollutants and potential impacts

5.1. Heavy metals and trace elements

Heavy metals and trace elements are present in coastal seas in limited amounts as non-degradable non-toxic naturally occurring free elements. They enter the marine and coastal environment in large quantities as concentrated but not particularly toxic free elements through different sources including domestic and industrial sewage effluent (Goldberg, 1995; Islam and Tanaka, 2004). However, their cationic forms are dangerous to living organisms because of their capacity to bind with short carbon chains. Once in the system, they bioaccumulate in protein-rich tissues such as liver and muscles of marine organisms and eventually human year by year, interfere with metabolic process and cause harmful effects (Davies, 1978).

The present review has reported nine studies with elevated levels of 11 heavy metals and trace elements with reference to international standards mainly in the coastal and marine areas (Table 1). These include 8 metals that are considered toxic and most poisonous to marine life, in the order of decreasing toxicity (Davies, 1978): mercury, cadmium, nickel, lead, copper, chromium, arsenic and zinc. However, most studies reviewed in the present paper including the de Mora et al. (2004) paper which examined marine pollution in the Gulf and Gulf of Oman based on sediment quality and quality of local seafood with respect to metal contamination have concluded that metal concentrations in different environmental media are in general lower or fell within the ranges reported previously for these metals by Fowler et al. (1993) except for some localized areas of chronic contamination which require continuous intensive monitoring strategies. Nevertheless, reporting normal concentrations should not hinder or cease monitoring and protection of marine environment in a potentially fragile area prone to a variety of contamination in addition to a delicate situation posed by high temperature, salinity and UV exposure.

Furthermore, this review has clearly indicated that the number of studies dealing with soil pollution is limited in this part of the world most probably due to the fact that most environmental studies were concentrated on the marine and coastal environment due to its socio-economic importance.

In addition, industrial activities in the Arabian Gulf are widely scattered along the coasts where major urban centers involving oil refineries, desalination plants, power stations, and port loading facilities are located (Sheppard et al., 2010). Therefore, since every pollutant whether originated on land or in the air tends to end up in the ocean (William, 1996); it seems that most research studies in the region have dealt with the effect of pollution on the marine environment rather than the inland environment.

Nevertheless, scarce environmental studies regarding soil pollution in the region can also be due to the facts that areas surrounding heavy industrial plants are not publically accessible and therefore considered as restricted zones that require official permission to access. Furthermore, substantial monitoring and assessment data are confined to the governmental sectors in addition to legislation limitations that hinder research projects. Therefore, monitoring and ongoing assessments of the environment surrounding industrial areas in the Gulf countries, soil contamination due to emissions and discharges from heavy industrial plants in the surrounding area, the control of toxic metal exposure in workplaces, toxic metal levels in industrial plants workers, if ever conducted, are never published and always considered as company's private information. In addition, communities located near industrial areas have never been screened and damage to public health has never been addressed. Therefore, such sensitive and important information which are confined to government reports may never be published and used for future temporal monitoring, environmental assessments, restoration, and remediation of contaminated areas as well as the establishment of baseline work (Freije, 2009; Freije and Dairi, 2009).

This review has also revealed that the trace elements lead and zinc were the main pollutants detected in the soil and air of the studied countries due to automobile emissions. This can be attributed to the fact that those studies were conducted prior to the introduction of unleaded gasoline in the region (Freije, 2009; Freije and Dairi, 2009).

Despite the fact that there are many environmental studies dealing with heavy metals and trace elements in the region, this review has clearly indicated that most studies conducted in the Arabian Gulf countries over the years considered that pollution due to heavy metals and trace element is the least problem facing the area in relation to others such as stresses from massive coastal developments that convert productive coastal areas into lands for homes, recreation and industrial facilities as well as ambitious projects including causeways and artificial islands (Erdelen, 2007; Sheppard et al., 2010). Discharges from desalination plants are another major problem this region is facing. Desalination plants discharge heated hyper-saline water in the sea which results in an adverse impact on marine environment (Gladstone et al., 1999). The highest capacity desalination plants are located in the Arabian Gulf countries and 70–90% of the population depends on them as the main source of fresh water. Therefore, appropriate distillation system technology should be implemented in order to prevent polluted discharges from reaching the sea area (Nadim et al., 2008).

5.2. Petroleum hydrocarbons

Many organic chemicals such as total petroleum hydrocarbon (TPH), total organic carbon (TOC), tributyltin (TBT), dibutyltin (DBT), monobutyltin (MBT), and polcyclic aromatic hydrocarbons (PAHs) were reported as high organic contaminants in five main studies in the present review in limited number of marine organisms and sediments (Table 1) (Fowler et al., 1993; Massoud et al., 1996; de Mora et al., 2003, 2010; Tolosa et al., 2005). However, most studies conducted in the area over the years using high quality monitoring techniques have observed considerable improvement in the area with petroleum hydrocarbon concentrations returning to baseline levels prior to 1991 oil spills in which more than 6 million barrels of crude oil were released into the marine environment (Kureishy, 1993; Literathy, 1993; Madany et al., 1996a; Sheppard et al., 2010).

The latest of these is de Mora et al. study (2010) which examined more than 14 years of accumulated data in a variety of environmental media and concluded that levels of petroleum hydrocarbons were comparable to those observed in relatively unpolluted areas elsewhere in the world such as the northwestern Mediterranean Sea (Tolosa et al., 1996), the eastern Mediterranean (Gogou et al., 2000), the Ukrainian coastline in the Black Sea (Readman et al., 2002), and Alaska (Page et al., 2005). Data collected from the reviewed papers which covers a period of nearly three decades can be considered as a comprehensive review of the existing status of the world's most vulnerable area in relation to oil pollution incidence. Such review would allow an efficient use of compiled information for future assessment of these contaminants in an area that produces some 25% of the world's oil (Khan, 2002).

6. Health risk assessment

Despite the obvious importance of the linkage between pollution and human health, enough baseline information regarding the specific effects of environmental contaminants on human health in the Arabian Gulf are not available. The present review has clearly shown that there is insufficient data regarding this matter, few direct examples of pollution effects on human health, as well as insufficient use of existing data (Vreeland and Ekarath, 1990; Al-Mahroos and Al-Saleh, 1997, 2000; Freije and Dairi, 2009). It is essential to identify goals as a first step in the evaluation of health risk associated with pollution in the Arabian Gulf. In general, these goals should involve the following general points:

- Identify major health problems that result from all sorts of pollution.
- Develop monitoring and surveillance programs for affected communities.
- Establish regional management plans in corporation with international authorities.
- Adopt effective control measures that involve local and international cooperation.
- Utilize available research knowledge in formulating legislation, and regulation.
- Gather extensive and effective research and baseline information.
- Identify objectives, classify issues and formulate management plans.

It is now believed that the least developed and developing nations are more likely to produce high levels of environmental

pollution due to the fact that they are characterized by their poor capacity to treat or recycle waste, poor legislation and regulation, poor management and protective measures in comparison to developed countries in which most waste treatment, safest disposal facilities and environmental management systems are adopted (Islam and Tanaka, 2004). Therefore, least developed and developing nations that comprise the major part of the world should be effectively engaged in global environmental protection and management programs that include long time periods of monitoring that include measurements of pollutants in different media which most probably will threaten human health through consumption of contaminated food and drinking water, breathing contaminated air and being exposed to contaminated soil as well as monitoring pollutants in human samples.

7. Conservation issues, needs, and strategic actions

The Arabian Gulf is a semi-enclosed sea with unique ecosystems that are susceptible to disturbance from human activities, the sharing of coasts by several countries, and therefore increased challenges to environmental management and conservation efforts. It is also characterized by limited exchange with the oceans which comprises increased chances of environmental damages from pollution, coastal and catchment habitat destruction, loss of biodiversity, and unsustainable use of resources (Madany et al., 1995; de Mora et al., 2004). The costs of rehabilitation are excessive, and usually beyond developing countries capacity (Kinder and Lintner, 1993). Therefore, protection from pollution is the first step and an essential goal for efficient and effective conservation and management of the Arabian Gulf.

Protection strategies will depend on regional preventive and curative agreements, strategic action program for sustainable use and conservation of the region's resources in coordination with international institutions such as the United Nation Development Program (UNDP), and the United Nations Environment Program (UNEP), as well as donor organizations including the Islamic Development Bank, and the World Bank. At the present time, the Regional Organization for Protection of Marine Environment (ROPME) is the only Sea Forum in the Gulf region that has made significant contribution toward the management and protection of the Gulf area (Nadim et al., 2008). ROPME includes the following eight member states: Bahrain, Iran, Iraq, Kuwait, Oman, Qatar, Saudi Arabia, and UAE; whereas the ROPME SEA Area (RSA) includes the Arabian Gulf, the Gulf of Oman and the southeastern coasts of Oman located in the Arabian Sea. Therefore, ROPME can be one of the most suitable candidates for future cooperation in the region (Nadim et al., 2008). Regional reviews of pollution issues in the Arabian Gulf including symptoms and impacts, immediate causes, root causes, scale and severity based on a series of country reports, analysis of regional environmental problems, assessment of threats are the key process in identifying priority regional actions with significant short and long term consequences for sustainable use and conservation.

8. Conclusion

This review has clearly indicated that heavy metal, trace element and petroleum hydrocarbon pollution in the coastal and marine area of the Arabian Gulf comprise most studies in the region in comparison to soil and air pollution. Despite the fact that the Arabian Gulf has been through substantial environmental stress regarding metal and petroleum hydrocarbon contamination due to the fact that it has been the scene of three wars over the past three decades; most studies reviewed here have indicated relatively low levels of heavy metal and petroleum residue contaminations in a variety of different habitats and organisms and that considerable improvement in the area with petroleum hydrocarbon concentrations returning to baseline levels prior to 1991 oil spills. In general, few studies have shown elevated concentrations of some pollutants indicating relatively low to moderately chronic contamination from oil and metals in certain areas, whereas chronically contaminated areas were limited to certain hot spots such as BAPCO site in Bahrain. Nevertheless, effective sustainable management and protective measures of coastal, marine, soil, and air environment in the Arabian Gulf area are of great importance and are essential for effective resource management and environmental decision making.

Acknowledgements

The author wishes to express her gratitude to the University of Bahrain for providing the literature required for the completion of this review. Many thanks are due to Dr. Humood A. Naser an assistant professor of Environmental Biology for proofreading this work.

References

Akhter, M.S., Al-Jowder, O., 1997. Heavy metal concentrations in sediments from the coast of Bahrain. Inter. J. Environ. Health Res. 7, 85–93.

Akhter, M.S., Madany, I.S., 1993. Heavy metals in street and house dust in Bahrain. Water, Air, Soil Pollut. 66, 111–119.

Al Farraj, S., El-Gendy, A.H., Alyahya, H., El-Hedeny, M., 2011. Heavy metals accumulation in the mantle of the common cuttlefish *Sepia pharaonis* from the Arabian Gulf. Aust. J. Basic. Appl. Sci. 5 (6), 897–905.

Al-Abdali, F., Massoud, M.S., Al-Ghadban, A.N., 1996. Bottom sediments of the Arabian Gulf-III. Trace metal contents as indicators of pollution and implications for the effect and fate of the Kuwait oil slick. Environ. Pollut. 93 (3), 285–301.

Al-Busaidi, M., Yesudhason, P., Al-Mughairi, S., Al-Rahbi, W.A.K., Al-Harthy, K.S., Al-Mazrooei, N.A., 2011. Toxic metals in commercial marine fish in Oman with reference to national and international standards. Chemosphere 84, 67–73.

Al-Hashimi, A.H., Al-Zorba, M.A., 1991. Mercury in some commercial fish from Kuwait: a pilot study. Sci. Total. Environ. 106, 71–82.

Al-Jedah, J.H., Robinson, R.K., 2001. Aspects of the safety of fish caught off the coast of Qatar. Food. Control. 12, 549–552.

Al-Mahroos, F., Al-Saleh, F., 1997. Lead levels in deciduous teeth of children in Bahrain. Ann. Trop. Paediatr. 17 (2), 147–154.

Al-Mahroos, F., Al-Saleh, F., 2000. Umbilical cord blood lead level of newborns in Bahrain. Curr. Pediatr. Res. 4 (1), 7–12.

Al-Majed, N.B., Preston, M.R., 2000. An assessment of the total and methyl mercury content of zooplankton and fish tissue collected from Kuwait territorial waters. Mar. Pollut. Bull. 40, 298–307.

Al-Saleh, I., Doush, I., 2002. Mercury contents in shrimp and fish species from the Gulf Coast of Saudi Arabia. Bull. Environ. Contam. Tox. 68, 576–583.

Al-Saleh, I., Shinwari, N., 2002. Preliminary report on the levels of elements in four fish species from the Arabian Gulf of Saudi Arabia. Chemosphere 48, 749–755.

Al-Saleh, I., Al-Doush, I., Echeverria-Quevedo, A., 1999. Residue of pesticides in grains locally grown in Saudi Arabia. Bull. Environ. Contam. Tox. 63, 451–459.

Al-Sayed, H.A., Mahasneh, A.M., Al-Saad, J., 1994. Variations of trace metal concentrations in seawater and pearl oyster *Pinctada radiate* from Bahrain (Arabian Gulf). Mar. Pollut. Bull. 28 (6), 370–374.

Al-Sayed, H., Al-Saad, J., Madany, I.M., Al-Hooti, D., 1996. Heavy metals in the grouper fish *Epinephelus coioides* from the cast of Bahrain: an assessment of monthly and spatial trends. Int. J. Environ. Stud. 50, 237–246.

Amin, M.B., Husain, T., 1994. Kuwaiti oil fires-Air quality monitoring. Atmos. Environ. 28 (13), 2261–2276.

Anderlini, V., Samhan, O., and Awayes, R. (1983). Detection and assessment of trace metal pollution in Kuwait's marine environment. Kuwait Institute for Scientific Research, State of Kuwait, Vol. 1, KISR 605.

Barness, D., Hamadah, M.A., Ottaway, J.M., 1976. The lead, copper and zinc contents of tree rings and bark. A measurement of local metallic pollution. Sci. Total Environ. 5, 63–67.

Bu-Olayan, A.H., Thomas, B.V., 2001. Arsenic levels in the marine ecosystem off the Kuwait coast. Arabian Gulf Environ. 21, 71–75.

Burns, K.A., Villeneuve, J.P., Anderlinl, V.C., Fowler, S.W., 1982. Survey of tar, hydrocarbon and metal pollution in the coastal waters of Oman. Mar. Pollut. Bull. 7, 240–247.

Cannon, H.L., Bowles, J.M., 1962. Contamination of vegetation by tetraethyl lead. Sci. 137, 765–766.

Coles, S.L., Gunay, N., 1989. Tar pollution on Saudi Arabian Gulf beaches. Mar. Pollut. Bull. 20 (5), 214–218.

Danish, S., Madany, I.M., 1992. Concentrations of nitrogen dioxide throughout the state of Bahrain. Environ. Pollut. (Barking, Essex: 1987) 77, 71–78.

Davies, A.G., 1978. Pollution studies with marine plankton; Part II, Heavy metals. Adv. Mar. Biol. 15, 381–508.

de Mora, S.J., Fowler, S.W., Cassi, R., Tolosa, I., 2003. Assessment of organotin contamination in marine sediments and biota from the Gulf and adjacent region. Mar. Pollut. Bull. 46, 401–409.

de Mora, S., Fowler, S.W., Wyse, E., Azemard, S., 2004. Distribution of heavy metals in marine bivalves, fish and coastal sediments in the Gulf and Gulf of Oman. Mar. Pollut. Bull. 49 (5–6), 410–424.

de Mora, S., Tolosa, I., Fowler, S.W., Villeneuve, J.-P., Cassi, R., Cattini, C., 2010. Distribution of petroleum hydrocarbons and organochlorinated contaminants in marine biota and coastal sediments from the ROPME Sea Area during 2005. Mar. Pollut. Bull. 60 (12), 2323–2349.

EC – European Communities (2001). Commission regulation (EC) No. 466/2001 of 8 March 2001 setting maximum levels for certain contaminants in foodstuff. *Official J. Eur. Commun.*, 1. 77/1.

Erdelen, W.R., 2007. Foreward. Aquat. Ecosyst. Health. Manage 103, 255.

FAO – Food and Agricultural Organization (1983). Compilation of legal limits for hazardous substances in fish and fishery products. Compiled by C.E. Nauen, FAO, Rome.

Fowler, S.W. (1985). Coastal baseline studies of pollutants in Bahrain, UAE and Oman ROPME Symposium on Regional Marine Monitoring and Research Programs UAE University, Al-Ain, UAE.

Fowler, S.W., Readman, J., Oregioni, B., Villeneuve, J., McKay, K., 1993. Petroleum hydrocarbons and trace metals in nearshore gulf sediments and biota before and after the 1991 war. Mar. Pollut. Bull. 27, 171–182.

Fowler, S.W., Villeneuve, J.-P., Wyse, E., Jupp, B., de Mora, S., 2007. Temporal survey of petroleum hydrocarbons, organochlorinated compounds and heavy metals in benthic marine organisms from Dhofar, southern Oman. Baseline 1. Mar. Pollut. Bull. 54, 339–367.

Freije, A., 2009. Blood Lead Level Determination in 2008 after Removal of Lead from Gasoline in 2000. JUBBAS 8, 50–60.

Freije, A., Awadh, M., 2009. Total and Methylmercury Intake Associated with Fish Consumption in Bahrain. Water. Environ. J. 23 (2), 155–164.

Freije, A., Dairi, M., 2009. Determination of Blood Lead Levels in Adult Bahraini Citizens Prior to the Introduction of Unleaded Gasoline and the Possible Effect of Elevated levels on the Serum Immunoglobulin IgG. Bahrain. Med. Bull. 31 (1), 17–20.

Gladstone, W., Tawfiq, N., Nasr, D., Andersen, I., Cheung, C., Drammeh, H., Krupp, F., Lintner, S., 1999. Sustainable use of renewable resources and conservation in the Red Sea and Gulf of Aden: issues, needs and strategic actions. Ocean. Coast. Manage. 42, 671–697.

Gogou, A., Bouloubassi, I., Stephanou, E.G., 2000. Marine organic geochemistry of the Eastern Mediterranean: 1. Aliphatic and polyaromatic hydrocarbons in Cretan Sea surficial sediments. Mar. Chem. 68, 265–282.

Goldberg, E.D., 1995. Emerging problems in the coastal zone for the twenty-first century. Mar. *Pollut*. Bull. 31 (4–12), 152–158.

Habashi, B., Al-Majed, N., Borhama, A., 1993. "Levels of trace metals in ROPME sea area". Final report of the scientific workshop on results of the R/V Mt. Mitchell Cruise in ROPME Sea Area 1, 73–86.

Islam, Md.S., Tanaka, M., 2004. Impact of pollution on coastal and marine ecosystems including coastal and marine fisheries and approach for management: a review and synthesis. Mar. *Pollut*. Bull. 48, 624–649.

Juma, H.A., Al-Madany, I.S., 2008. Concentration of heavy metals in the territorial sea water of the Kingdom of Bahrain, Arabian Gulf, Arab Gulf. J. Sci. Res. 26 (1/2), 19–32.

Kehrig, H.A., Malm, O., Moreira, I., 1998. Mercury in a widely consumed fish *Micropogonias furnieri* (Demarest, 1823) from four main Brazilian estuaries. Sci. Total Environ. 213, 263–271.

Khamdan, S.A.A., Al Madany, I.M., 2009. Temporal and spatial variations of the quality of ambient air in the Kingdom of Bahrain during 2007. Environ. Monit. Assess. 154, 241–252.

Khan, N.Y. (2002). Physical and human geography. In: Khan, N. Y., Munawar, M., Price, A. R. G. (Eds.), The Gulf Ecosystem: Health and Sustainability. Backhuys Publishers, Leiden, pp. 3–21.

Khordagui, H., Al-Ajmi, D., 1991. Mercury in seafood: A preliminary risk assessment for Kuwaiti consumers. Environ. Int. 17, 429–435.

Kinder, J., Lintner, S.F., 1993. An action plan to clean up the Baltic. Environment 35, 7–31.

Kureishy, T.W., 1993. Concentration of heavy metals in marine organisms around Qatar before and after the Gulf War oil spill. Mar. Pollut. Bull. 27, 183–186.

Literathy, P., 1993. Considerations for assessment of environmental consequences of the 1991 Gulf War. Mar. Pollut. Bull. 27, 349–356.

Madany, I.M., Danish, S., 1988. Measurement of air pollution in Bahrain. Environ. Int. 14 (1), 49–58.

Madany, I.M., Ali, S.M., Akhter, M.S., 1990. Assessment of lead in roadside vegetation in Bahrain. Environ. Inter. 16 (2), 123–126.

Madany, I.M., Akhter, M.S., Al Jowder, O.A., 1994. The correlations between heavy metals in residential indoor dust and outdoor street dust in Bahrain. Environ. Inter. 20 (4), 483–492.

Madany, I.M., Abbas, Z., Al-Alawi, A., 1995. Trace metals concentrations in marine organisms from the coastal areas of Bahrain, Arabian Gulf. Water. Air. Soil. Pollut. 91 (3–4), 233–248.

Madany, I.M., Al-Aawi, Z., Abdul Wahab, A., 1996a. Trace metal concentrations in marine organisms from the coastal area of Bahrain. Water. Air. Soil. Pollut. 91, 233–248.

Madany, I.M., Akhter, M.S., Raveendran, E., 1996b. Spatial and temporal patterns in heavy metals concentration in street dust in a hot desert region. Int. J. Environ. Health. Res. 6, 93–101.

Massoud, M.S., Al-Abdali, F., Al-ghadban, A.N., Al-Sarawi, M., 1996. Bottom sediments of the Arabian Gulf-II. TPH and TOC contents as indicators of oil pollution and implications for the effect and fate of the Kuwait oil slick. Environ. Pollut. 93 (3), 271–284.

Mathews, C.P., Kedidi, S., Fita, N.I., Al-Yahya, A., Al-Rasheed, K., 1993. Preliminary assessment of the effects of the 1991 Gulf War on Saudi Arabian prawn stocks. Mar. Pollut. Bull. 27, 251–271.

Nadim, F., Bagtzoglou, A.C., Iranmahboob, J., 2008. Costal management in the Persian Gulf region within the framework of the ROPME programme of action. Ocean. Coast. Manage. 51, 556–565.

Nakagawa, R., Yumita, Y., Hiromoto, M., 1997. Total mercury intake from fish and shellfish by Japanese people. Chemosphere 35, 2909–2913.

Naser, H.A., 2010. Testing taxonomic resolution levels for detecting environmental impacts using macrobenthic assemblages in tropical waters. Environ. Monit. Assess. 170, 435–444.

Oostdam, B.L., 1984. Tar pollution of beaches in the Indian Ocean, the South China Sea and the Pacific Ocean. Mar. Pollut. Bull. 15, 267–270.

Page, D.S., Boehm, P.D., Brown, J.S., Neff, J.M., Burns, W.A., Bence, A.E., 2005. Mussels document loss of bioavailable polycyclic aromatic hydrocarbons and the return to baseline conditions for oiled shorelines in Prince William Sounds, Alaska. Mar. Environ. Res. 60, 422–436.

Price, A.R.G., Sheppard, C.R.C., Roberts, C.M., 1993. The Gulf: its biological setting. Mar. Pollut. Bull. 27, 5–15.

Quinche, J.P., Zuber, R., Boray, E., 1969. Lead pollution of highway roadside vegetation. Phytopathol. Z. 66, 259–274.

Readman, J.W., Fillmann, G., Tolosa, I., Bartocci, J., Villeneuve, J.-P., Catinni, C., Mee, L.D., 2002. Petroleum and PAH contamination of the Black Sea. Mar. Pollut. Bull. 44, 48–62.

Reynolds, R.M., 1993. Physical oceanography of the Gulf, Straits of Hormuz and the Gulf of Oman. Results from the Mt Mitchell expedition. Mar. Pollut. Bull. 27, 35–59.

Sadiq, M., McCain, J.C., 1993. Effect of the 1991Gulf war on metal bioaccumulation by the clam (*Meretrix meretrix*). Mar. Pollut. Bull. 27, 163–170.

Sadiq, M., Mian, A.A., 1994a. Lead, titanium and zinc in air particulate at Dhahran, Saudi Arabia, during and after Kuwait oil fires. Sci. Total. Environ. 152 (2), 113–118.

Sadiq, M., Mian, A.A., 1994b. Nickel and vanadium in air particulate at Dhahran (Saudi Arabia) during and after the Kuwait oil fires. Atmos. Environ. 28 (13), 2249–2253.

Sadiq, M., Saeed, T., Fowler, S.W., 2002. Seafood contamination. In: Khan, N.Y., Munawar, M., Price, A.R.G. (Eds.), The Gulf Ecosystem: Health and Sustainability. Backhuys Publishers, Leiden, pp. 327–351.

SASO, Saudi Arabian Standards Organization (1997). Maximum limits of contaminating metallic elements in foods, Riyadh, Saudi Arabia.

Sheppard, C.R., 1993. Physical environment of the Gulf relevant to marine pollution: an overview. Mar. Pollut. Bull. 27, 3–8.

Sheppard, C., Al-Husiani, M., Al-Jamali, F., Al-Yamani, F., Baldwin, R., et al, 2010. The Gulf: a young sea in decline. Mar. Pollut. Bull. 60, 13–38.

Tolosa, I., Bayona, J.M., Albaiges, J., 1996. Aliphatic and aromatic hydrocarbons and sulfur/oxygen derivatives in northwestern Mediterranean sediments: spatial and temporal variability, fluxes, and budgets. Environ. Sci. Tech. 30, 2495–2503.

Tolosa, I., de Mora, S.J., Fowler, S.W., Villeneuve, J.-P., Bartocci, J., Cattini, C., 2005. Aliphatic and aromatic hydrocarbons in marone biota and coastal sediments from the Gulf and the Gulf of Oman. Mar. Pollut. Bull. 50, 1619–1633.

UN, United Nations Group of Experts on Geographical Names (2006). Working Paper No. 61, 23rd Session, Vienna, 28 March – 4 April 2006. <http://unstats.un.org/unsd/geoinfo/ungegn/docs/23gegn/wp/gegn23wp61.pdf>.

UNEP – United Nations Environment Programme (1991). Sampling of selected marine organisms and sample preparation for the analysis of chlorinated hydrocarbons. Reference methods for marine pollution studies No. 12, Rev. 2, UNEP, Nairobi, p. 17.

USFDA – US Food and Drug Administration (1979). Action level for mercury in fish, shellfish, crustaceans, and other aquatic animals. Fed. Reg., 44(14): 3990-4012.

USFDA Food and Drug Administration (1993a). Guidance document for chromium in shellfish, DHHA/PHS/FDA/CFSAN/Office of seafood, Washington, DC.

USFDA Food and Drug Administration (1993B). Guidance document for arsenic in shellfish, DHHA/PHS/FDA/CFSAN/Office of seafood, Washington, DC.

USFDA Food and Drug Administration (1993c). Guidance document for nickel in shellfish, DHHA/PHS/FDA/CFSAN/Office of seafood, Washington, DC.

USFDA Food and Drug Administration (2006). Mercury levels in commercial fish and shellfish. Available: www.cfsan.fda.gov/-frf/sea-mehg.

Vreeland, W.J., Ekarath, R., 1990. Lead in air and blood in the state of Bahrain. Environ. Tech. 11, 491–498.

WHO, World Health Organization 1993. Evaluation of certain food additives and contaminants. Forty-first report of the joint FAO/WHO Expert Committee in Food Additives. WHO Technical Report Series No. 837. World Health Organization, Geneva.

William, C., 1996. Combating marine pollution from land-based activities: Australian initiatives. Ocean. Coast. Manage. 33 (1–3), 87–112.
