# Supplementary material for: Methods Using Marine Aquatic Photoautotrophs along the Qatari Coastline to Remediate Oil and Gas Industrial Water
Source: Toxics. 2024 Aug 24;12(9):625. doi: 10.3390/toxics12090625 (PMC11435476; doi:10.3390/toxics12090625)
Supplement: Supplementary file 1 [file toxics-12-00625-s001.zip › Table S4.pdf]

Egyptian Journal of Aquatic Research 48 (2022) 21–27

HOSTED BY

Contents lists available at ScienceDirect

Egyptian Journal of Aquatic Research

journal homepage: www.sciencedirect.com/locate/ejar

Full length article

Pollution indices of heavy metals in the Western Arabian Gulf coastal area

Sameh A. Amin, Hanan Almahasheer *

Department of Biology, College of Science, Imam Abdulrahman Bin Faisal University (IAU), Dammam 31441-1982, Saudi Arabia

ARTICLE INFO

Article history:
Received 14 July 2021
Revised 7 October 2021
Accepted 10 October 2021
Available online 17 November 2021

Keywords:
Pollution
Arab Gulf
Water
Soil sediment
Trace metals
Pollution index PI
Sediments pollution index SPI

ABSTRACT

An environmental assessment of the Western Arabian Gulf Coastal regions was done by assessing heavy metals in sediments and seawater, as well as other environmental parameters. A total of 94 sediment samples and 94 water samples were collected from 22 locations to estimate 12 heavy metals in each sample. This was achieved by using Inductively Coupled Plasma-Optical Emission Spectrometer -ICP-OES and Direct Mercury Analyzer. In general, metals were significantly higher in sediment samples compared with water samples. The pollution index PI and sediments pollution index SPI proved that the study areas were unpolluted to slightly polluted with regard to sediments and water except for one location. Cadmium showed polluting levels in five sites of the studied area, thus it was considered as the most polluting heavy metal among the other studied metals. Zinc was contaminating two sites, while each of Mn, Cu, Pb, and Cr reached pollution levels in one site. On the other hand, Fe, Ni and Hg had non-polluting levels. The study revealed that about 82% of the Arabian Gulf western coasts are non-polluted to slightly polluted.

© 2021 National Institute of Oceanography and Fisheries. Hosting by Elsevier B.V. This is an open access article under the CC BY-NC-ND license (http://creativecommons.org/licenses/by-nc-nd/4.0/).

Introduction

The accretion of heavy metals in the ecosystem causes a sequence of possible hazards in the biological systems. The health status of the marine environment of the coastal regions is of global and local concern as it is fundamental to humankind (Siddiquee et al. 2012). The Saudi Arabian coast, which extends along the Arabian Gulf receives a variety of contamination sources that could pollute the marine environment and is affected by anthropogenic activities. Such activities encompass the growing infrastructure at the Eastern Saudi coast, and the several industrial campuses that were established during the last 40 years. Activities such as fisheries, maritime cultivation, transport, and tourism, together with a deficiency in wastewater treatments, are a threat to the marine life (Böhlmark 2003). The effluents from these activities discharged into the shallow semi-closed water body of the Gulf have caused major disturbance to the coastal environment (Khan and Al-Homaid 2003; Alyahya et al. 2011). High concentrations of heavy metals in the marine ecosystems are an important pollutant as they can be toxic and have a potential to enter the food chain. These metals bio-accumulating in marine organisms may exceed the threshold limits and are then considered as a danger to the environment (Rainbow, 2002; Alyahia et al., 2011). Nour (2020) reported that some metals in the coastline are naturally detected due to the weathering processes. In addition, they are present due to anthropogenic activities such as drilling, fisheries, and delivery activities. The use of marine sediments as a tool to monitor metals pollution could be better than using seawater (Nour and Nouh, 2020a). Freije (2015) reviewed sources of pollutants in the Arabian Gulf ecosystem, which have conspicuous effects on the ecosystem and the human well-being. Emphasis is placed on marine pollution, particularly toxic metal, and petroleum hydrocarbon contaminations. Without any doubt, the 1991 Gulf War was hostile to the environment, and incurred adverse changes associated with the marine habitats (Jones et al., 2018). The aim of this study was to evaluate the pollution levels in the coastal sediments and water of the Western Arabian Gulf coast using heavy metals analysis. This investigation will help as a standard for pollution studies in the area, and will be considered as indicative of the current ecosystem health.

Peer review under responsibility of National Institute of Oceanography and Fisheries.

* Corresponding author.
E-mail address: halmahasheer@iau.edu.sa (H. Almahasheer).

https://doi.org/10.1016/j.ejar.2021.10.002
1687-4285/© 2021 National Institute of Oceanography and Fisheries. Hosting by Elsevier B.V.
This is an open access article under the CC BY-NC-ND license (http://creativecommons.org/licenses/by-nc-nd/4.0/).

Materials and methods

Study area

The Arabian Gulf is considered as an extremely important aquatic ecosystem, it is known for its oil–gas production (Hamza and Munawar 2009). However, the Gulf suffers from natural and anthropogenic stresses (Naser 2013) such as extreme harsh conditions of elevated levels of temperature and salinity (Sheppard et al. 2010), a history of oil spills (Jones et al. 2008), and coastal human pressure, which resulted in the deterioration and loss of important habitat of the gulf ecosystem (Vaughan et al. 2019).

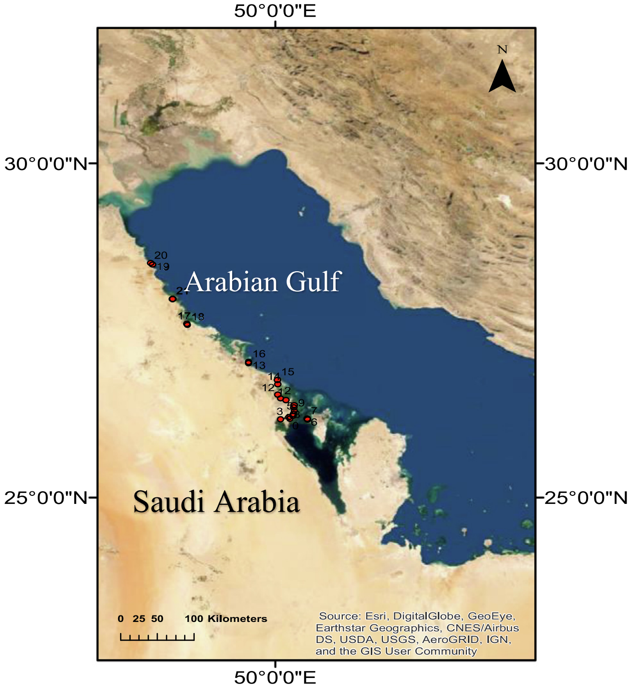

Fig. 1. Location points sampled in Saudi Arabia and Bahrain, Western Arabian Gulf. The map was produced by the researchers using World Imagery layer with ArcMap Version 10.2.

Sampling of sediment and water

Twenty-two sites were randomly sampled along the Western Arabian Gulf coastal area between September and November 2020. The latitudes and longitudes of each location are provided in Fig. 1 and Table 1. Three to five replicates of the top 5 cm of sediment (Mudroch and Azcue 1995), and surface water samples were acquired from these sites with a total number of n = 94 sediment samples and n = 94 water samples using Polypropylene bottles. Parameters as pH, salinity, and electrical conductivity (EC) were measured for water and sediment samples directly in the lab using a Seven Go Duo pro (Mettler Toledo).

Chemical analysis and data quality

Sediment samples were first dried at 60℃, and ground with a granite mortar, then sieved to remove particles > 2 mm. Afterwards, 100 mg of soil were digested in a microwave mineralization (microwave Milestone Ethos one) using a 3:1 mixture of HCl 37% and HNO3 65% following the EPA method 3052 (Gaudino et al., 2007). Water samples were taken in a polyethylene container and acidified by the addition of 2 mL of HNO3 in 1 L of sample. The samples were kept in the lab at 4 °C. Then the heavy metals were analyzed on the same day. The heavy metals in both sediment and water samples were; Arsenic (As), cadmium (Cd), cobalt (Co), chromium (Cr), copper (Cu), iron (Fe), manganese (Mn), molybdenum (Mo), nickel (Ni), lead (Pb), zinc (Zn) and Mercury (Hg). They were determined by using the Inductively Coupled Plasma-Optical Emission Spectrometer-ICP-OES; Varian 720-ES-(Clesceri et al., 1998). Mercury (Hg) of both water and sediment samples was analyzed using the Direct Mercury Analyzer (DMA-80, Milestone) according to the Application Note: HG/EN-05 and Application Note: HG / EN-09 in the equipment manual. Metals analysis was carried out in the lab of the Minstry of Enviroment, Water, and Agriculture – Eastren Province – Saudi Arabia. Data Accuracy was according to the quality control certificate ISO 17025, which is provided in (Table 2).

The pollution index (PI) of the investigated elements was evaluated by using the following equation of Jorfi et al. (2017): PI = Element concentration (Cn) / Reference value of element (warning standard Bn). Whereas sediments pollution index (SPI) was calculated according to the equation modified by Jorfi et al. (2017).

Table 1
Latitudes and longitudes of different sampled locations in Saudi Arabia and Bahrain in the Western Arabian Gulf.

Number	Country	Location	Latitude	Longitude
1	Bahrain	Galali	26°16′00″	50°39′44″
2	KSA	Half Moon	26°16′44″	50°06′77″
3	Bahrain	Amwaj Island	26°17′16″	50°39′27″
4	KSA	Al Aziziyah	26°17′42″	50°18′21″
5	KSA	Al Buhairah An Nawras	26°19′49″	50°16′65″
6	KSA	Ishbilia	26°21′61″	50°21′81″
7	KSA	Alkhobar Corniche	26°24′37″	50°22′03″
8	KSA	New Alkhobar Corniche	26°28′89″	50°23′77″
9	KSA	Al Bahar	26°34′46″	50°22′55″
10	KSA	As Sadafah	26°37′57″	50°23′02″
11	KSA	Dammam Corniche	26°45′36″	50°13′24″
12	KSA	Saihat	26°47′50″	50°06′71″
13	KSA	Al-Qatif	26°53′68″	50°03′12″
14	KSA	Ras Tanoura Sea Port	26°69′05″	50°02′91″
15	KSA	Ras Tanoura Corniche	26°75′50″	50°02′70″
16	KSA	Al Jubail	27°00′57.11″	49°67′32.72″
17	KSA	Al Jubail sea port	27°02′50″	49°67′50″
18	KSA	Manifa (2)	27°57′67629	48°92′60.31″
19	KSA	Manifa (1)	27°59′4818	48°91′70.65″
20	KSA	Al Saffaniyah	27°96′975	48°74′25″
21	KSA	Corniche Al Khafji	28°48′22778	48°49′683.33″
22	KSA	Al Khafji (2)	28°50′1209	48°47′48.19″

Table 2
Data Accuracy according to the quality control certificate ISO 17025.

Element	Uncertainty	Expected error	Accuracy
Co	5.05 %	± 0.0505 ppm	94.95 %
Cr	4.95 %	± 0.0495 ppm	95.05%
Fe	4.2 %	± 0.0420 ppm	95.80%
Zn	5.41 %	± 0.0541 ppm	94.59%
Mo	4.7 %	± 0.0470 ppm	95.30%
Ni	6.53 %	± 0.0653 ppm	93.47%
As	6.14 %	± 0.0614 ppm	93.86%
Cd	7.44 %	± 0.0744 ppm	92.56%
Pb	6.57 %	± 0.0657 ppm	93.43%
Hg	8.15 %	± 0.0815 ppm	91.85%
Cu	3.51 %	± 0.0351 ppm	96.49%
Mn	3.92 %	± 0.0392 ppm	96.08%

$$SPI = \frac{\sum \frac{Heavy\ metal\ concentration\ in\ soil}{Soil\ contamination\ warning\ standard}}{Number\ of\ heavy\ metals}$$

And the warning standard was calculated from Youssef et al. (2015) and Alharbi et al. (2017).

Statistical analysis

Descriptive statistics and Pearson correlation analysis were used to test for significant relationships among detected elements in the studied sediments and water samples. All statistics were computed using SPSS 26. All detected elements are provided for the reader in the Supplementary Material (Table S1 for sediment samples and Table S2 for water samples).

Results

The tested parameters in the studied locations revealed comparable results for the pH and salinity values for both water and sediment samples. The pH values of the examined sediment samples ranged from 6.13 to 8.84 with an average of 7.52 ± 0.53, whereas the corresponding values of the Gulfs' water samples were between 6.73 and 8.54 with an average of 7.92 ± 0.41 (Table 3 and 4). In contrast, salinity exhibited a significant increase in water samples compared to sediment samples, with an average of 43 PSU in water samples compared to 9 PSU for the sediment. Similarly, the EC followed the same pattern with an average of 4 ms/cm for sediments compared to 15 ms/cm for water samples. Further information of the pH, salinity and EC averages in both water and sediment along the twenty-two sampled locations in the Western Arabian Gulf coast are provided in Table 5 and 6. However, unexpectedly, one value was very low in Al-Qatif with 12.9 PSU, and this could be because the sampled location was near a discharge area (Table 6).

In general, metals were significantly higher in sediment samples than water samples (Table 3 and 4). Overall, three metals, arsenic, cobalt, and molybdenum, were not detected (ND) in sediment samples, while two metals, cadmium and chromium, were not detected (ND) in water samples (Table 3 and 4). In addition,

Table 3
Descriptive statistics of sediment samples.

Parameters tested and elements		% Detected samples#	Minimum	Maximum	Mean	Std. Deviation
pH		100 (94)	6.13	8.84	7.52	0.533
Salinity (PSU)		100 (94)	2.20	17.70	9.089	2.711
Conductivity (ms/cm)		100 (94)	4.23	28.90	15.610	4.325
Elements (PPM)	Iron	100 (94)	239.0	3231.0	1460.0	75.60
	Manganese	96.81 (91)	0.378	113.865	34.300	26.458
	Zinc	84.04 (79)	0.117	76.317	6.939	10.255
	Chromium	91.49 (86)	0.291	37.695	6.844	7.174
	Nickel	100 (94)	0.485	9.423	3.932	2.249
	Copper	100 (94)	0.394	9.467	2.294	1.991
	Lead	93.62 (88)	0.230	3.409	0.919	0.578
	Cadmium	94.68 (89)	0.016	1.166	0.168	0.168
	Mercury	100 (94)	0.001	0.088	0.013	0.017
	Arsenic	ND				
	Cobalt	ND				
	Molybdenum	ND				

(ND) not detected.

Table 4
Descriptive statistics water samples.

Parameter		% Detected samples#	Minimum	Maximum	Mean	Std. Deviation
pH		100 (94)	6.727	8.542	7.917	0.4048
Salinity (PSU)		100 (94)	12.20	80.10	43.052	11.7209
Conductivity (ms/cm)		100 (94)	20.60	110.10	63.926	15.3174
Elements (PPM)	Iron	100 (93)	0.001	0.844	0.046	0.1173
	Zinc	19.15 (18)	0.000	0.141	0.025	0.0351
	Nickel	100 (94)	0.015	0.022	0.018	0.0013
	Lead	97.88 (92)	0.002	0.020	0.010	0.0036
	Arsenic	40.43 (38)	0.000	0.027	0.010	0.0078
	Molybdenum	100 (94)	0.002	0.017	0.010	0.0026
	Manganese	96.81 (91)	0.000	0.076	0.004	0.0093
	Copper	86.17 (81)	0.000	0.017	0.003	0.0026
	Mercury	100 (94)	0.400	5.400	0.001	0.6652
	Cobalt	4.26 (4)	0.000	0.002	0.001	0.0008
	Cadmium	ND				
	Chromium	ND				

(ND) not detected.

Table 5
Mean Values of Physical and chemical features and heavy metals in the studied Locations of the Arabian Gulf Shore Sediments.

No.	Location	pH	Salinity psu	EC ms/cm	Fe ppm	Mn ppm	Zn ppm	Mo ppm	Ni ppm	Cu ppm	Pb ppm	Hg ppm	As ppm	Co ppm	Cr ppm	Cd ppm
1	Galali	7.38	8.74	15.12	2826	98.443	13.964	ND	6.879	7.774	1.077	0.007	ND	ND	23.352	0.292
2	Half Moon	7.85	11.86	20.06	1625	40.676	7.542	ND	4.524	1.491	1.074	0.004	ND	ND	7.279	0.233
3	Amwaj Island	7.53	7.12	12.28	1391.2	33.104	1.116	ND	3.292	1.326	0.345	0.003	ND	ND	4.763	0.122
4	Al Aziziyah	7.76	8.52	14.74	2077	58.272	5.660	ND	4.439	1.471	0.401	0.006	ND	ND	15.548	0.173
5	Al buhairah An Nawras	7.34	16.64	27.32	626.4	5.374	2.113	ND	1.508	0.718	0.788	0.045	ND	ND	1.253	0.128
6	Ishbilia	8.09	8.34	14.44	1027.0	21.471	0.643	ND	1.712	0.772	0.427	0.002	ND	ND	10.143	0.075
7	Alkhobar Corniche	7.65	11.4	19.32	1052.6	19.068	6.715	ND	2.943	2.827	0.888	0.016	ND	ND	2.386	0.099
8	New Alkhobar Corniche	7.68	9.76	16.7	1641.6	41.748	3.840	ND	5.185	1.983	0.876	0.022	ND	ND	5.684	0.343
9	Al Bahar	7.70	9.76	16.86	1409	30.512	4.614	ND	4.326	1.706	0.621	0.008	ND	ND	5.785	0.308
10	As Sadafah	7.65	9.2	15.98	1547.4	28.432	8.449	ND	5.003	3.061	1.154	0.011	ND	ND	5.290	0.165
11	Dammam Corniche	8.34	8.74	15.22	2065.2	53.037	5.160	ND	5.451	1.851	1.694	0.061	ND	ND	5.572	0.265
12	Saihat	8.31	7.64	13.36	1362.2	34.605	31.765	ND	5.51	2.605	1.694	0.017	ND	ND	8.139	0.210
13	Al Qatif	6.91	9.18	15.58	2770.2	71.755	27.897	ND	9.141	7.162	1.379	0.021	ND	ND	13.150	0.298
14	Ras Tanoura Sea Port	6.16	9.967	17.1	2310.67	67.849	6.033	ND	7.395	2.440	0.934	0.013	ND	ND	13.012	0.209
15	Ras Tanoura Corniche	7.48	7.234	12.767	297.67	3.631	0.435	ND	0.765	0.594	0.435	0.002	ND	ND	0.393	0.018
16	Al Jubail	8.03	2.52	4.78	1111.4	20.050	4.961	ND	3.530	1.514	0.781	0.003	ND	ND	4.345	0.072
17	Al Jubail sea port	7.54	6.4	11.267	1484.67	34.109	4.863	ND	4.077	1.883	1.577	0.007	ND	ND	4.907	0.135
18	Manifa (1)	6.74	9.767	16.747	691.67	10.238	0.117	ND	1.647	0.729	0.493	00.3	ND	ND	4.709	0.051
19	Manifa (2)	6.65	10.70	18.20	926.33	11.197	0.709	ND	2.282	1.165	2.380	0.005	ND	ND	2.195	0.062
20	Al Saffaniyah	6.69	8.867	15.3	1631	28.184	1.868	ND	2.622	2.262	0.769	0.013	ND	ND	4.089	0.153
21	Corniche Al Khafji	7.09	8.134	14.10	369	1.274	ND	ND	1.339	1.226	0.519	0.002	ND	ND	ND	0.023
22	Al Khafji (2)	7.12	7.934	13.80	494.67	6.663	ND	ND	1.546	1.156	0.415	0.004	ND	ND	2.417	0.029

(ND) not detected.

Table 6
Mean Values of Physical and chemical features and heavy metals in the studied locations of the Arabian Gulf shore water.

No.	Location	pH	Salinity psu	EC ms/cm	Fe ppm	Mn ppm	Zn ppm	Mo ppm	Ni ppm	Cu ppm	Pb ppm	Hg ppm	As ppm	Co ppm	Cr ppm	Cd ppm
1	Galali	8.09	41.3	61.88	0.061	0.005	0.029	0.010	0.005	0.003	0.011	0.0008	0.009	ND	ND	ND
2	Half Moon	8.10	58.8	83.84	0.010	ND	ND	0.012	0.017	0.002	0.013	0.0008	0.001	ND	ND	ND
3	Amwaj Island	8.03	40.4	60.56	0.030	0.005	ND	0.010	0.017	0.003	0.011	0.0015	0.007	ND	ND	ND
4	Al Aziziyah	7.96	50.9	74.3	0.022	0.005	0.002	0.010	0.017	0.002	0.010	0.0008	0.007	ND	ND	ND
5	Al buhairah An Nawras	7.74	77.4	106.45	0.071	0.004	0.011	0.015	0.018	0.004	0.008	0.0027	0.005	ND	ND	ND
6	Ishbilia	7.29	48.6	71.46	0.007	ND	ND	0.012	0.017	0.002	0.011	0.0009	0.010	ND	ND	ND
7	Alkhobar Corniche	8.19	43.7	65.22	0.017	ND	ND	0.012	0.017	0.001	0.013	0.0010	0.007	ND	ND	ND
8	New Alkhobar Corniche	8.02	42.5	63.44	0.020	0.005	ND	0.010	0.017	0.002	0.011	0.0008	0.006	ND	ND	ND
9	AL Bahar	7.96	41.6	62.38	0.017	ND	0.050	0.010	0.018	0.002	0.010	0.0007	0.005	ND	ND	ND
10	As Sadafah	8.03	42.6	64.1	0.013	ND	ND	0.009	0.017	0.001	0.007	0.0008	0.004	ND	ND	ND
11	Dammam Corniche	8.38	39.1	59.02	0.220	0.010	0.003	0.011	0.020	0.002	0.013	0.0016	ND	0.002	ND	ND
12	Saihat	8.42	37.8	57.38	0.082	0.013	0.011	0.013	0.018	0.001	0.013	0.0010	0.005	ND	ND	ND
13	Al Qatif	7.09	12.9	21.84	0.345	0.023	0.063	0.007	0.017	0.011	0.011	0.0006	0.006	ND	ND	ND
14	Ras Tanoura Sea Port	7.97	43.8	65.23	0.022	0.003	ND	0.009	0.017	0.002	0.009	0.0008	ND	ND	ND	ND
15	Ras Tanoura Corniche	7.74	38.6	58.37	0.026	0.002	ND	0.008	0.016	0.017	0.006	0.0009	ND	ND	ND	ND
16	Al Jubail	7.65	37.8	57.44	0.012	0.001	ND	0.009	0.017	0.001	0.009	0.0005	0.008	ND	ND	ND
17	Al Jubail sea port	7.44	37.4	56.87	0.056	0.002	0.004	0.006	0.017	0.003	0.007	0.0011	0.005	ND	ND	ND
18	Manifa (1)	8.52	41.4	62.8	0.035	0.001	0.022	0.006	0.017	0.004	0.008	0.0008	0.002	ND	ND	ND
19	Manifa (2)	7.90	42.8	63.7	ND	ND	ND	0.007	0.018	0.002	0.006	0.0008	0.010	ND	ND	ND
20	Al Saffaniyah	7.75	41.9	62.63	ND	ND	ND	0.005	0.010	ND	0.006	0.0011	ND	0.001	ND	ND
21	Corniche Al Khafji	7.85	38.8	59.8	ND	ND	0.010	0.006	0.017	0.002	0.017	0.0010	0.026	0.001	ND	ND
22	Al Khafji (2)	7.28	39.6	59.43	ND	ND	ND	0.006	0.018	0.005	0.018	0.0010	0.024	ND	ND	ND

(ND) not detected.

other elements were not detected in some of the samples' locations. Further details are shown in Table 5 and 6. As mentioned earlier, all of the detected metals were higher in the sediments compared to the water, especially iron that was 31739-fold higher, followed by manganese 8575-fold higher, Copper with 765-fold higher, zinc and nickel with about 200-fold higher, and finally lead and mercury with 92 and 13-fold higher respectively (Fig. 2).

Whereas most parameters did not correlate with metals in the sediments, salinity correlated negatively with both zinc and cobalt, and positively with molybdenum in water samples (Table 7 and 8). Moreover, most of metals significantly correlated together, while some did not show any significant relation; for example iron, manganese, chromium, and nickel in sediment, and iron manganese, zinc, cobalt, and molybdenum in water samples (Table 7 and 8).

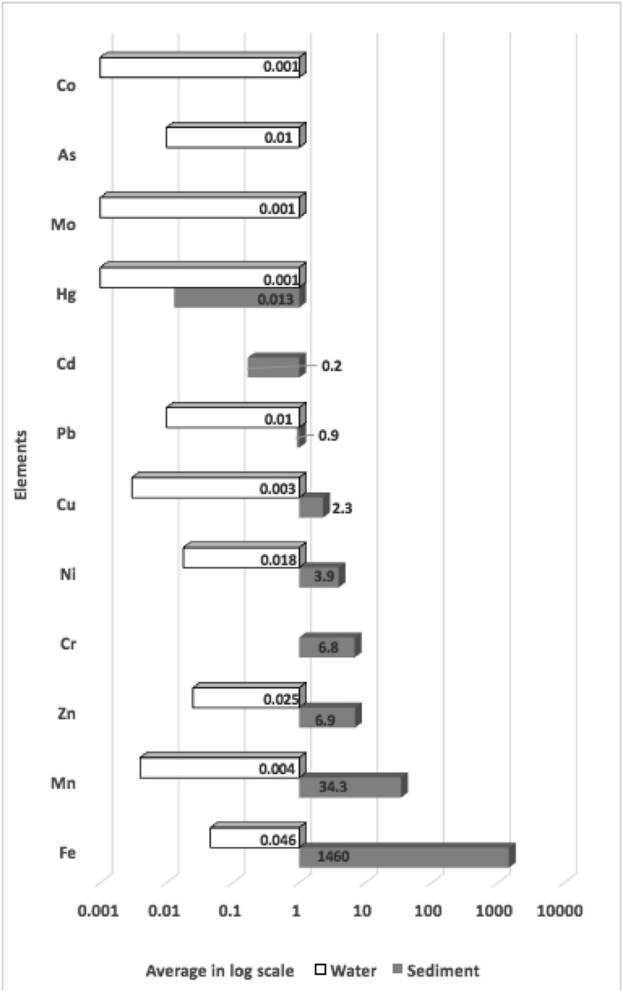

Fig. 2. Comparison in log scale of metals average values in water and sediment samples along the Western Arabian Gulf.

Finally, no correlation was found between latitudes and metals or parameters.

Data presented in Table 9 are showing the pollution index PI values of the investigated heavy metals and sediments pollution index (SPI) for the studied locations. In general, most of the locations were non– to slightly polluted, except three locations with moderate pollution (Al Aziziyah, Al-Qatif, and Ras Tanoura sea port), and one location that seemed to be highly polluted (Galali).

Discussion

The pollution of marine environment by metals is a serious problem and is usually associated with industrialized wastes (Elshorbagy, 2015) because of the definitive accumulation of those pollutants in the marine sediment. The average metal levels were found in sediments with the order of: Fe > Mn > Zn > Cr > Ni > Cu > Pb > Cd > Hg. While in water they were at the following order: Fe > Zn > Ni > Pb > As > Mo > Mn > Cu > Hg > Co. Iron was the highest element of all analyzed elements in both the sediment and water samples compared to the other metals. Recently, Alshemmari and Talebi (2019) found in the surface sediments of the northwestern Arabian Gulf, Kuwait, that the average heavy metal abundance was in the sequence of Pb> Co> Cu> Zn> Cr> Ni. The heavy metal accumulation in sediments (Table 3 and 5) was much higher than that in water samples (Table 4 and 6). This may be explained by the scarce solubility of heavy metals in general. Higher levels of the heavy elements are associated with fine-grained materials that indicate the origin of the Earth (Nour & El-Sorogy, 2020). Strongly positive correlations were recorded between all investigated heavy metals in sediment and water samples in general. The correlation matrix of sediments revealed strong correlations between Fe and Mn with Zn, Ni, Hg, Cu, Co, Pb and Cd indicating a relationship in their geochemical origin. Moreover, significant positive correlations were found between several heavy metals; Hg correlated with Cu and Zn concentrations (r = 0.842, 0.716 and 0.722, respectively), but it had no correlation with Cr (r value of −0.083), and Cu correlated with Cr, Cd, Ni, Pb, Zn, and Hg (r = 0.592, 0.331, 0.730, 0.313, 0.730 and 0.051,

Table 7
Correlations matrix among detected elements in the studied sediments. Person correlation ranging between −1 and 1. The negative values indicate indirect irreversible correlation, while the positive ones indicate direct relationships.

Elements		pH	Sal. psu	EC ms/cm	Fe g/kg	Mn ppm	Cu ppm	Cr ppm	Cd ppm	Ni ppm	Pb ppm	Zn ppm	Hg ppm
pH	P.C	1											
	Sig												
Salinity psu	P.C	−0.243	1										
	Sig	0.018*											
EC ms/cm	P.C	−0.240	0.999	1									
	Sig	0.020*	0.000**										
Iron g/kg	P.C	−0.039	−0.048	−0.045	1								
	Sig	0.709^{N}	0.647^{N}	0.669^{N}									
Manganese ppm	P.C	−0.038	−0.084	−0.079	0.956	1							
	Sig	0.719^{N}	0.421^{N}	0.448^{N}	0.000**								
Copper ppm	P.C	−0.188	−0.037	−0.036	0.706	0.735	1						
	Sig	0.050*	0.726^{N}	0.729^{N}	0.000**	0.000**							
Chromium ppm	P.C	−0.017	−0.092	−0.087	0.781	0.848	0.592	1					
	Sig	0.870^{N}	0.379^{N}	0.404^{N}	0.000**	0.000**	0.000**						
Cadmium ppm	P.C	0.106	0.116	0.119	0.488	0.452	0.331	0.317	1				
	Sig	0.310^{N}	0.266^{N}	0.253^{N}	0.000**	0.000**	0.001**	0.002**					
Nickel ppm	P.C	−0.108	−0.035	−0.032	0.903	0.855	0.730	0.602	0.482	1			
	Sig	0.299^{N}	0.740^{N}	0.756^{N}	0.000**	0.000**	0.000**	0.000**	0.000**				
Lead ppm	P.C	0.061	0.081	0.081	0.318	0.258	0.313	0.083	0.238	0.333	1		
	Sig	0.558^{N}	0.435^{N}	0.435^{N}	0.002**	0.012*	0.002**	0.429^{N}	0.021*	0.001**			
Zinc ppm	P.C	0.077	−0.091	−0.091	0.435	0.420	0.622	0.360	0.272	0.441	0.476	1	
	Sig	0.462^{N}	0.382^{N}	0.382^{N}	0.000**	0.000**	0.000**	0.000**	0.008**	0.000**	0.000**		
Mercury ppm	P.C	0.187	0.381	0.382	0.137	0.102	0.051	−0.083	0.223	0.196	0.344	0.177	1
	Sig	0.050*	0.000**	0.000**	0.187^{N}	0.329^{N}	0.626^{N}	0.428^{N}	0.031*	0.050*	0.001**	0.050*	

P.C. Pearson Correlation, *Significant at 0.05, **Significant at 0.01, ^{N} non-significant.

Table 8
Correlations matrix among detected elements in studied Water Samples. Person correlation ranging between −1 and 1. The negative values mean indirect irreversible correlation, while the positive one indicate direct relationships.

The parameter		pH	Salinity psu	EC ms/ cm	Fe ppm	Mn ppm	Cu ppm	Ni ppm	Pb ppm	Zn ppm	Hg ppb	Co ppm	As ppm	Mo ppm
pH	N	94	94	94	93	91	81	94	92	18	94	4	38	94
	P.C.	1												
	Sig.													
Salinity psu	P.C.	0.275	1											
	Sig.	0.007**												
Conductivity ms/cm	P.C.	0.306	0.998	1										
	Sig.	0.003**	0.00**											
Iron ppm	P.C.	-0.012	-0.220	-0.240	1									
	Sig.	0.912 ^{N}	0.034*	0.020*										
Manganese ppm	P.C.	-0.110	-0.365	-0.388	0.843	1								
	Sig.	0.297^{N}	.000**	0.00**	0.00**									
Copper ppm	P.C.	-0.394	-0.251	-0.287	0.532	0.615	1							
	Sig.	0.000**	0.024*	0.009**	0.00**	.000**								
Nickel ppm	P.C.	-0.080	0.046	0.039	0.335	0.249	0.080	1						
	Sig.	0.445^{N}	0.658^{N}	0.712^{N}	0.001**	0.017*	0.476^{N}							
Lead ppm	P.C.	0.188	-0.095	-0.094	0.198	0.138	-0.005	0.061	1					
	Sig.	0.073^{N}	0.368^{N}	0.372^{N}	0.050*	0.198^{N}	0.963^{N}	0.561^{N}						
Zinc ppm	P.C.	-0.434	-0.493	-0.510	0.417	0.735	0.732	0.201	-0.072	1				
	Sig.	0.050*	0.038*	0.030*	.085N	.001**	0.001**	0.424^{N}	0.777^{N}					
Mercury ppb (**ug/L**)	P.C.	0.022	0.402	0.386	0.011	-0.054	0.015	0.171	-0.088	-0.236	1			
	Sig.	0.830^{N}	0.00**	0.00**	0.916 ^{N}	0.612^{N}	0.893^{N}	0.050*	0.406^{N}	0.345^{N}				
Cobalt ppm	P.C.	0.386	-0.837	-0.836	0.803	0.799	-0.216	0.707	0.621	0.292	-0.784	1		
	Sig.	0.614^{N}	0.163^{N}	0.164^{N}	0.197^{N}	0.201^{N}	0.784^{N}	0.293^{N}	0.379^{N}	0.811^{N}	0.216^{N}			
Arsenic ppm	P.C.	-0.349	-0.213	-0.215	-0.267	-0.141	0.272	0.236	0.432	0.142	-0.152	1.000	1	
	Sig.	0.032*	0.200^{N}	0.195^{N}	0.050*	0.400^{N}	0.119^{N}	0.153^{N}	0.007**	0.762^{N}	0.362^{N}	0.000**		
Molybdenum ppm	P.C.	0.440	0.533	0.519	0.049	-0.003	-0.195	-0.023	0.192	-0.456	0.364	-0.445	-0.479	1
	Sig.	0.000**	0.00**	0.00**	0.640^{N}	0.979^{N}	0.081^{N}	0.823^{N}	0.050*	0.050*	0.000**	0.555^{N}	0.002**	

*Significant (Sig.) at 0.05 - **Significant at 0.01 - ^{N} non-significant - P.C. Pearson Correlation.

Table 9
Pollution Index (PI) and Sediments Pollution index (SPI) of heavy metals in the investigated sediments.

No.	Location	Fe ppm	Mn ppm	Zn ppm	Mo ppm	Ni ppm	Cu ppm	Pb ppm	Hg ppm	As ppm	Co ppm	Cr ppm	Cd ppm	SPI	P. Level
1	Galali	0.819	1.31	0.795	ND	0.536	1.363	0.222	0.007	ND	ND	1.040	1.292	4	H
2	Half Moon	0.472	0.541	0.429	ND	0.353	0.262	0.221	0.004	ND	ND	0.330	1.031	1.32	S
3	Amwaj Island	0.404	0.440	0.064	ND	0.257	0.233	0.071	0.003	ND	ND	0.211	0.539	0.86	N
4	Al Aziziyah	0.603	0.780	0.322	ND	0.346	0.258	0.083	0.006	ND	ND	0.689	0.765	2.18	M
5	Al buhairah An Nawras	0.182	0.071	0.120	ND	0.118	0.126	0.162	0.045	ND	ND	0.056	0.566	0.38	N
6	Ishbilia	0.298	0.286	0.037	ND	0.133	0.135	0.088	0.002	ND	ND	0.450	0.332	1.35	S
7	Alkhobar Corniche	0.305	0.254	0.382	ND	0.229	0.496	0.183	0.016	ND	ND	0.106	0.438	0.82	N
8	New Alkhobar Corniche	0.477	0.556	0.219	ND	0.404	0.348	0.181	0.022	ND	ND	0.252	1.518	1.17	S
9	Al Bahar	0.409	0.406	0.263	ND	0.337	0.299	0.128	0.008	ND	ND	0.256	1.363	1.09	S
10	As Sadafah	0.449	0.378	0.481	ND	0.389	0.537	0.238	0.011	ND	ND	0.234	0.73	1.26	S
11	Dammam Corniche	0.599	0.706	0.294	ND	0.425	0.325	0.349	0.061	ND	ND	0.247	1.173	1.27	S
12	Saihat	0.395	0.460	1.810	ND	0.429	0.457	0.349	0.017	ND	ND	0.361	0.929	1.75	S
13	Al Qatif	0.804	0.955	1.590	ND	0.712	1.250	0.284	0.021	ND	ND	0.583	1.319	2.89	M
14	Ras Tanoura Sea Port	0.670	0.903	0.343	ND	0.576	0.428	0.193	0.013	ND	ND	0.577	0.923	2.13	M
15	Ras Tanoura Corniche	0.086	0.048	0.025	ND	0.059	0.104	0.090	0.002	ND	ND	0.017	0.080	0.18	N
16	Al Jubail	0.323	0.267	0.282	ND	0.275	0.266	0.161	0.003	ND	ND	0.193	0.319	0.87	N
17	Al Jubail sea port	0.431	0.454	0.277	ND	0.318	0.330	0.325	0.007	ND	ND	0.218	0.597	1.11	S
18	Manifa (1)	0.021	0.136	0.007	ND	0.128	0.128	0.102	00.3	ND	ND	0.209	0.226	0.73	N
19	Manifa (2)	0.269	0.149	0.040	ND	0.178	0.204	1.107	0.005	ND	ND	0.097	0.274	0.72	N
20	Al Saffaniyah	0.473	0.375	0.196	ND	0.204	0.397	0.159	0.013	ND	ND	0.181	0.677	0.95	N
21	Corniche Al Khafji	0.107	0.017	ND	ND	0.104	0.215	0.107	0.002	ND	ND	ND	0.102	0.29	N
22	Al Khafji (2)	0.144	0.089	ND	ND	0.121	0.203	0.086	0.004	ND	ND	0.107	0.128	0.55	N

Pollution class/level (P level): N = non-polluted, S = slightly polluted, M = moderately polluted and H = highly polluted, (ND) not detected.

respectively). On the other hand, there was no correlation between the physical properties (pH, salinity & EC) and the majority of heavy metals concentrations in sediments. The only correlation was found with Hg, Pb and Cd.

Generally, a locally elevated concentration for one metal does not indicate elevated values for the others. This also applies to sources of biogeochemical pollution (Fahmy et al. 1997; Alharbi et al. 2017).

Pollution index PI indicated that Cd showed polluted levels in five sites of the studied area, thus it was considered the most polluting metal among the other studied metals. Zn was recorded as having polluted levels in two sites (Saihat & Al-Qatif). Each of

Mn, Cu, Pb, and Cr reached pollution levels in one site. On the other hand, wherever Fe, Ni and Hg were recorded, they showed unpolluted levels. Similarly, Almasoud et al. (2015) found that Cr concentrations in the Arabian Gulf sediment were in heavy and moderate levels of pollution. Furthermore, El-Taher et al. (2018) found high values of Cd in Ras Tanoura sea port samples.

With regard to the sediments pollution index SPI, one site (Galali) recorded a high pollution level with SPI 4, which could be a result of heavy transportation activities (Numbeo, 2021). Three locations namely Aziziah, Al-Qatif, and Ras Tanoura sea port recorded SPI 2:3 with moderate pollution levels. The majority of the studied sites exhibited non-pollution to slightly pollution levels with a percentage of 81.9% of the total studied sites. And the mean values of both water and sediment samples were within the ambient marine water/ sediment quality limit of the Abu Dhabi standard produced by Suleiman et al. (2020).

Conclusion

Many people depend upon the Arabian Gulf resources, which are called nowadays the blue economy. Therefore, pollution should be avoided, and such important marine habitats should be protected. The present results showed that heavy metals concentrations varied widly. Anthropogenic activities are mostly considered the highest source of pollution in the Arabian Gulf. The study revealed that about 82% of the Western Arabian Gulf coasts are showing non-polluted to slightly polluted levels.

Funding

Authors did not get financial support.

HYPERLINK "SPS:role::contributing" Author contribution

Equal contribution.

Animal research (Ethics)

Not applicable.

Declaration of Competing Interest

The authors declare that they have no known competing financial interests or personal relationships that could have appeared to influence the work reported in this paper.

Acknowledgment

We are thankful to the Science College Research Units at Imam Abdulrahman Bin Faisal University (IAU), for providing the chemicals, and to the lab of the Minstry of Enviroment, Water, and Agriculture – Eastren Province – Saudi Arabia for analyzing the metals. Also, we would like to thank Dr. Ahsan Shemsi and Dr. Mohamed Guitouni for their help.

Appendix A. Supplementary data

Supplementary data to this article can be found online at https://doi.org/10.1016/j.ejar.2021.10.002.

References

Alharbi, T., Alfaifi, H., El-Sorogy, A., 2017. Metal pollution in Al-Khobar seawater, Arabian Gulf, Saudi Arabia. Marine Pollution Bulletin 119 (1), 407–415.

Almasoud, F.I., Usman, A.R., Al-Farraj, A.S., 2015. Heavy metals in the soils of the Arabian Gulf coast affected by industrial activities: Analysis and assessment using enrichment factor and multivariate analysis. Arabian Journal of Geosciences 8 (3), 1691–1703.

Alshemmari, H., Talebi, L., 2019. Heavy metal concentrations in the surface sediments of the northwestern Arabian Gulf, Kuwait. Arabian Journal of Geosciences 12 (18), 1–9.

Alyahya, H.; El-Gendy, A.; Al Farraj, S. & El-Hedeny, M. (2011). Evaluation of Heavy Metal Pollution in the Arabian Gulf Using the Clam *Meretrix meretrix* Linnaeus, 1758. *Water, Air, & Soil Pollution*, V. 214, P: 499–507.

Böhlmark J. (2003). *Meretrix meretrix* as an indicator of heavy metal contamination in Maputo Bay. A Thesis Work at Uppsala University School of Engineering Program for Aquatic and Environmental Engineering. Department of Earth Sciences, Uppsala University, Sweden.

El-Taher, A., Alshahri, F., Elsaman, R., 2018. Environmental impacts of heavy metals, rare earth elements and natural radionuclides in marine sediment from Ras Tanura, Saudi Arabia along the Arabian Gulf. Applied Radiation and Isotopes 132, 95–104.

Elshorbagy, Walid (2015). Overview of marine pollution in the Arabian Gulf with emphasis on pollutant transport modeling. The First International Conference on Coastal Zone Management and Engineering in the Middle East held at Dubai, United Arab Emirates, on 27th–29th November, 1-20.

Freije, A.M., 2015. Heavy metal, trace element and petroleum hydrocarbon pollution in the Arabian Gulf: Review. Journal of the Association of Arab Universities for Basic and Applied Sciences 17 (1), 90–100. https://doi.org/10.1016/j.jaubas.2014.02.001.

Hamza, W., Munawar, M., 2009. Protecting and managing the Arabian Gulf: Past, present and future. Aquatic Ecosystem Health & Management 12 (4), 429–439.

Jones, D. A., Hayes, M., Krupp, F., Sabatini, G., Watt, I., & Weishar, L. (2008). The impact of the Gulf War (1990–91) oil release upon the intertidal Gulf coast line of Saudi Arabia and subsequent recovery. In Protecting the Gulf's Marine Ecosystems from Pollution (pp. 237-254): Springer.

Khan, M.A., Al-Homaid, N.A., 2003. Remote sensing study on mangrove depletion, Tarut Bay, Saudi Arabia. In: Alsharhan, A.S. (Ed.), Desertification in the third millennium. A. A. Balkema, Rotterdam, pp. 227–234.

Mudroch, A., Azcue, J.M., 1995. Manual of aquatic sediment sampling. CRC Press.

Naser, H.A., 2013. Assessment and management of heavy metal pollution in the marine environment of the Arabian Gulf: A review. Marine pollution bulletin 72 (1), 6–13.

Numbeo (2021). https://www.numbeo.com/pollution/country_result.jsp?country=Bahrain.

Nour, H.E., El-Sorogy, A.S., 2020. Heavy metals contamination in seawater, sediments and seashells of the Gulf of Suez, Egypt. Environmental Earth Sciences 79 (11).

Nour, H. E., Nouh, E., (2020a) Comprehensive pollution monitoring of the Egyptian Red Sea Coast by using the environmental indicators. Environ Sci. Pollution Res (ISSN 0944-1344). 10.1007/s11356-020-09079-3.

Rainbow, Philip S, 2002. Trace metal concentrations in aquatic invertebrates: Why and so what? Environmental Pollution 120 (3), 497–507.

Siddiquee, N.A.; Parween1, S.; Assers, M.A. and Barua, P. (2012). Heavy Metal Pollution in Sediments at Ship Breaking Area of Bangladesh. In: Coastal Environments: Focus on Asian Regions. Ed: V. Subramanian. Springer.

Sheppard, C., Al-Husiani, M., Al-Jamali, F., Al-Yamani, F., Baldwin, R., Bishop, J., et al., 2010. The Gulf: A young sea in decline. Marine Pollution Bulletin 60 (1), 13–38. https://doi.org/10.1016/j.marpolbul.2009.10.017.

Suleiman, W., Anbiah, R., Al Raisi, A., Al Hosani, S., Barber, M., Whaley, G., 2020. Development of ambient marine standards using long-term data: An example from Abu Dhabi. Aquatic Ecosystem Health & Management 23 (2), 154–165.

Vaughan, G.O., Al-Mansoori, N., Burt, J.A., 2019. The arabian gulf. In: World seas: An environmental evaluation. Elsevier, pp. 1–23.
